# The effect of overweight/obesity on diastolic function in children and adolescents: A meta‐analysis

**DOI:** 10.1111/cob.12476

**Published:** 2021-07-18

**Authors:** Samuel Burden, Benjamin Weedon, Luke Whaymand, Josefien Rademaker, Helen Dawes, Alexander Jones

**Affiliations:** ^1^ Centre for Movement, Occupational and Rehabilitation Sciences Oxford Institute of Nursing, Midwifery and Allied Health Research, Oxford Brookes University Oxford UK; ^2^ Department of Paediatrics University of Oxford Oxford UK; ^3^ NIHR Oxford Health Biomedical Research Centre Oxford Health NHS Foundation Trust Oxford UK

**Keywords:** children, diastolic function, obesity

## Abstract

Left ventricular diastolic function (LVDF) is an important marker of early cardiovascular remodelling, which has not been well summarized in young people with overweight/obesity. Weighted, random‐effects regression was used to determine the strength of associations of both body mass index (BMI) and homeostatic model assessment of insulin resistance (HOMA‐IR) with LVDF measures, adjusting for age and sex. Six databases were searched after PROSPERO registration (CRD42020177470) from inception to July 2020 for studies that compared LVDF between overweight/obesity and control groups aged ≤24 years, yielding 70 studies (9983 individuals). Quality and risk of bias were assessed using NHLBI tools, with scores of good, fair, and poor for 6, 48, and 16 studies, respectively. Increased BMI was associated with worse LVDF in all measures except early mitral inflow deceleration time, with septal early diastolic tissue peak velocity to late diastolic tissue peak velocity ratio having the strongest association (*n* = 13 studies, 1824 individuals; *r* = −0.69; *P* < 0.001). Elevated HOMA‐IR was also associated with worse LVDF. Although we could not determine the causality of reduced LVDF in young people, our findings should aid the development of paediatric guidelines for the assessment of LVDF and support further work to address the longitudinal consequences of childhood obesity and IR on LVDF.

**Abbreviations**
A wave: late mitral inflow peak velocity
*a*′: late diastolic tissue peak velocity
BMI: body mass index
BMIz: body mass index z‐score
CMRFs: cardiometabolic risk factors
CVD: cardiovascular disease
DT: early mitral inflow peak velocity deceleration time
E wave: early mitral inflow peak velocity
*e*′: early diastolic tissue peak velocity
*E*/*A*
: early mitral inflow peak velocity/late mitral inflow peak velocity ratio
*E*/*e*′: early mitral inflow peak velocity/early diastolic tissue peak velocity ratio
*e*′/*a*′: early diastolic tissue peak velocity to late diastolic tissue peak velocity ratio
HF: heart failure
HOMA‐IR: homeostatic model assessment of insulin resistance
IR: insulin resistance
IVRT: isovolumic relaxation time
LA: left atrium
LAP: left atrial pressure
LV: left ventricle
LVDF: left ventricular diastolic function
LVDD: left ventricular diastolic dysfunction
OW/Ob: overweight and obesity
SD: standard deviation
SE: standard error
TDI: tissue Doppler imaging
T1D: Type 1 diabetes

## 1 INTRODUCTION

Overweight and obesity (OW/Ob) are globally important health disorders that affect all age‐groups. Together, their prevalence has increased by 47.1% in children, compared to 27.5% in adults, between 1980 and 2013, resulting in approximately 23% of children in developed countries being classified as OW/Ob.^1^ The early‐onset diseases that result from childhood OW/Ob place a costly burden on economies, estimated at $14 billion a year for America alone.^2^ Cardiovascular disease (CVD) contributes most to this, accounting for 68.6% of all obesity‐related deaths.^3^ Abnormalities of left ventricular diastolic function (LVDF) are major contributors to such CVD, with >80% of diastolic heart failure (HF) patients being overweight or obese.^4^

**FIGURE 1:**
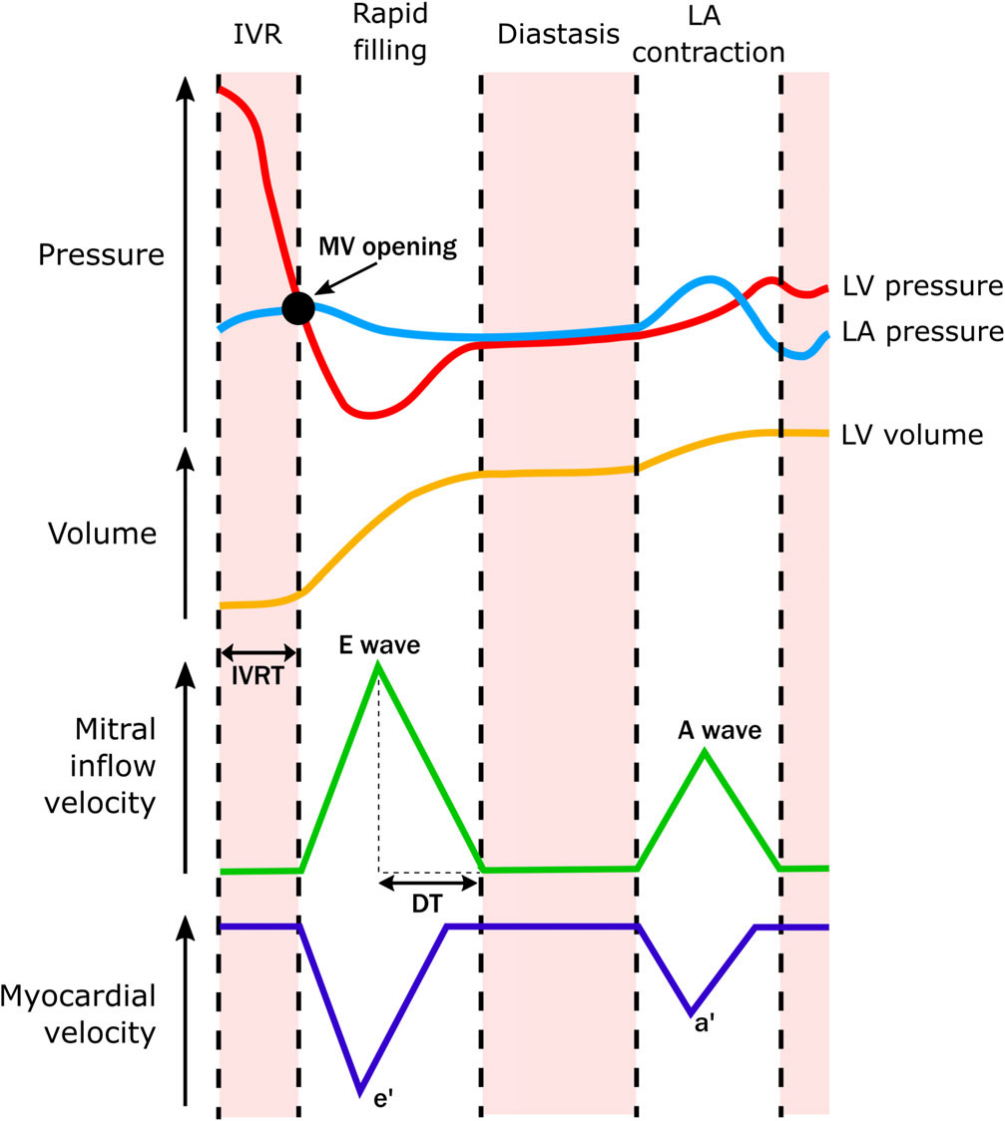
Stages of diastole and echocardiography measures of diastolic function. Stage I—isovolumic relaxation (IVR) which occurs after aortic valve closure and before mitral valve opening, as left ventricle (LV) pressure falls rapidly until it reaches left atrial pressure, prompting mitral valve (MV) opening; Stage II—rapid filling, where the MV is open and blood is suctioned towards the apex of the LV from the left atrium (LA), which occurs as the myocardium lengthens during falling LV pressure; Stage III—diastasis, after initial filling where LA and LV pressures equalize and flow ceases; Stage IV—LA contraction, which generates an additional pressure gradient that drives more blood into the LV. A wave indicates late mitral inflow peak velocity; *a*′, late diastolic tissue peak velocity; DT, E wave deceleration time; E wave, early mitral inflow peak velocity; e′, early diastolic tissue peak velocity; IVRT, isovolumic relaxation time

LVDF describes the ability of the left ventricle (LV) to fill with blood during diastole, which completes in four stages: (I) isovolumic relaxation; (II) rapid filling; (III) diastasis; and (IV) left atrium (LA) contraction (Figure 1). Numerous physiological parameters influence LVDF, including the rate of early myocardial lengthening in diastole, and thus filling, which is determined by a combination of active (energy‐utilizing) and passive forces. Impaired LVDF includes a number of pathological processes, including impaired relaxation, increased myocardial stiffness, and elevated LA pressure (LAP).

An array of interrelated indices obtained by echocardiography or cardiovascular magnetic resonance (CMR) exist to indirectly assess LVDF. Although these are all subject to influence by the factors described above, there is evidence that some measures may be better at differentiating particular elements of LV diastolic dysfunction (LVDD) than others. For example, some measures are less influenced by LAP.

Tissue Doppler imaging (TDI) measures longitudinal myocardial motion at the basal septum or lateral ventricular wall in response to inflow. TDI measures differ from conventional Doppler ultrasound measures of transmitral blood flow velocity because they assess longitudinal rather than global compliance of the ventricle, and are less influenced by LV loading conditions.^5^, ^6^, ^7^ It has been suggested that these differences may improve their ability to detect early LVDD,^7^ particularly in conditions such as obesity where volume overload occurs.

The chronic volume overload and metabolic abnormalities of obesity are associated with progressive LVDD and eventual diastolic HF, through impairment of myocardial relaxation and passive LV properties.^8^ Detection methods for LVDD in adults are well‐established,^9^ but it is unclear which measures best detect the earliest stages of LVDD and would, therefore, be most suitable in adolescents and particularly in those with OW/Ob. Reliable early detection is important as LVDD reversibility is still potentially achievable and lifestyle habits may be less fixed than in adulthood.

Although there are many studies of LVDF in children with obesity, diverse methods and group definitions have made it difficult to adequately summarize findings by conventional meta‐analysis, with study heterogeneity being identified as the main limiting factor in earlier attempts.^10^, ^11^ Nevertheless, statistical synthesis should be possible, given that most studies share common measures of adiposity and LVDF. This would confirm whether or not OW/Ob is associated with impaired LVDF at a young age, which is yet to be clearly established.

Although most studies focus on measures of adiposity, a significant number have addressed other cardiometabolic risk factors (CMRFs), such as insulin resistance (IR),^12^, ^13^, ^14^ that often accompany OW/Ob and may directly impair LVDF. Where data allow, we aimed to assess the relationships of these other CMRFs with LVDF.

In this systematic review and meta‐analysis, our aims were to determine in a population of children and adolescents: (1) the extent to which OW/Ob is associated with various measures of LVDF; (2) measures of LVDF which are most strongly associated with OW/Ob and; (3) the associations of IR and other CMRFs with LVDF measures.

## 2 METHODS

The protocol for this review was registered with PROSPERO International Prospective Register of Systematic Reviews (identifier CRD42020177470). This review was completed in accordance with the PRISMA 2020 (Preferred Reporting Items for Systematic Reviews and Meta‐Analyses) guidelines.^15^

## 3 CRITERIA FOR CONSIDERING STUDIES FOR THIS REVIEW

### 3.1 Types of studies

Cross‐sectional studies, controlled intervention studies, and pre‐post studies that examined the association of childhood and adolescent OW/Ob with LVDF were included. Inclusion was limited to full‐text articles reported in English and published in peer‐reviewed journals. We excluded studies published in grey literature sources and conference or meeting abstracts without a full text.

### 3.2 Types of participants

Individuals aged <18 years were included in accordance with the international definition of childhood. Additionally, individuals aged 10–24 years were included and defined as adolescents in accordance with the widest accepted definition to ensure that articles that used this definition were not rejected.^16^ It has been suggested that this definition of adolescence corresponds best with contemporary features of adolescent growth and social role transitions.^16^ Almost every study used individual criteria to define their OW/Ob and control groups. Furthermore, the pathological group in some studies was obesity only while others included overweight in this group. In other studies, overweight was grouped with normal weight as a control group. Therefore, study‐specific group definitions are reported in the results, but were ignored in the meta‐analysis as this heterogeneity did not allow for meaningful group‐based comparisons.

### 3.3 Types of outcome measures

Primary outcomes were measures of LVDF (see Supporting Information for a comprehensive list of individual LVDF measures) (Figure 1). Where both septal and lateral TDI measures were reported without their commonly reported mean, this was calculated using the recommended Cochrane method (Supporting Information).^17^ TDI measurements were sometimes reported without mention of the site of measurement. These were assessed separately and are identified as “cannot determine” measures. There were insufficient data for some measures of LVDF, such as pulmonary vein peak velocities and diastolic strain rate, to be included in the meta‐analysis. To address this, a systematic review was completed to ensure that all measures of LVDF were summarized (Supporting Information).

## 4 SEARCH METHODS FOR IDENTIFICATION OF STUDIES

### 4.1 Electronic searches

Search terms were devised by one author and checked by another. Common terms and key words such as obesity, children and diastolic function were combined in search hedges (Supporting Information) and were applied in PubMed.gov (1958 to present), Cumulative Index to Nursing and Allied Health Literature (CINAHL; 1992 to present), Cochrane Central Register of Controlled Trials, and ClinicalTrials.gov (1997 to present), Embase (1974 to present), and Web of Science (1987 to present). The reference lists of included studies, as well as pertinent reviews,^10^, ^11^, ^18^ were also searched, yielding four further studies.^19^, ^20^, ^21^, ^22^ The final search was completed on 11th July 2020.

## 5 DATA COLLECTION AND ANALYSIS

### 5.1 Selection of studies

Four authors independently reviewed results of the search to include/exclude studies for full‐text screening. Inclusion and exclusion criteria for progression to the full‐text screening are documented in the Supporting Information. One author completed a preliminary screen of all potential papers for full‐text review to ensure that all included articles reported LVDF.

Two independent full‐text screens were completed to include/exclude studies for the review. Criteria for inclusion/exclusion to the full‐text screening are documented in the Supporting Information. When the same data were apparently reported in separate/duplicate publications, the article with the greatest number of subjects was selected and the other(s) excluded. However, if the article with fewer subjects reported additional LVDF measures, these measures were included as a separate study. Consensus on disagreements was achieved by discussion between reviewing authors or with the inclusion of a fifth author.

### 5.2 Data extraction and management

Data were extracted by one author using a pre‐defined form and verified for completeness and correctness by two other authors. The following data were extracted: (1) study characteristics and methods; (2) subject/group demographics; (3) homeostatic model assessment of insulin resistance (HOMA‐IR) results; (4) measures of LVDF and their results, and where applicable; (5) correlation statistics with adiposity and/or CMRF measures.

### 5.3 Assessment of risk of bias in included studies

Four authors independently executed quality assessment of the included studies and any discrepancies were resolved by discussion. Modified versions of the Study Quality Assessment Tools by the National Heart, Lung, and Blood Institute (NHLBI) were used to assess study quality and risk of bias.^23^ Scores of “good” (least risk of bias), “fair” (susceptible to some bias) and “poor” (significant risk of bias) were given to each study based on study design and implementation. Studies that were scored as “poor” overall but were otherwise methodologically sound (e.g. correctly reported LVDF measures and reported BMI, age, and sex) were included in the meta‐analysis. A sensitivity analysis was completed to ensure that these studies did not influence the results. Furthermore, details of how these tools assess quality and risk of bias are given in the Supporting Information.

### 5.4 Data synthesis

Measures of LVDF were transformed into standard units of measurement where necessary. Mean ± standard deviation (SD) were calculated from alternative descriptions of central tendency and dispersion e.g. median, using the recommended Cochrane tools (Supporting Information).^17^, ^24^

### 5.5 Statistical analysis

Analysis was completed using STATA (version 16.1, StataCorp, College Station, TX). Although group data are reported in study descriptions, the marked heterogeneity in the mean BMI of control and OW/Ob groups across studies limited our ability to do a conventional, group‐based meta‐analysis reliably. To overcome this, mean (SD) BMI values for all groups, regardless of how those groups were defined by authors, were used to assess continuous associations of BMI with LVDF measures, using weighted, random‐effects linear regression. These models were adjusted for age and sex to account for their known effect on BMI. These models also took account of the fact that some group means (e.g. a normal and an obesity group) were drawn from the same study. Each study was treated as a unique level in the random‐effects regression, allowing the pairwise differences within studies to be captured by the model without reliance on specific group definitions. This enabled estimation of the linear relationships of BMI with multiple measures of LVDF and their relative strength, giving insight into which measurements may be most useful for early detection of impaired LVDF.

HOMA‐IR values were similarly used to assess continuous associations with LVDF measures, using weighted, random‐effects linear regression. These models were also adjusted for age and sex.

To account for individual study size and measure variance, each group estimate in the random‐effects regression models was weighted using the inverse‐variance method (1/standard error [SE]^2^).^17^ The SE of each measure was calculated using the SD and N reported for each group.

Histogram plots were used to assess normality of variables. Any non‐normally distributed variables were transformed into normal distributions using the Tukey Ladder of Powers using the transformation with the smallest chi‐squared value. Measures were further transformed to their *z*‐scores (LVDFz, BMIz and HOMA‐IRz), to allow correlation coefficients (*r*) to be calculated. Fisher's *z*‐test was used to compare the strength of these correlations with the strongest association as a reference (Supporting Information). Robust *z*‐scores, which do not depend on parametrically distributed data, were also calculated (Supporting Information) and the analyses were repeated to check that non‐parametric distributions were not responsible for the findings. The brand of echocardiography machine was further included as a variable in repeat analyses to determine whether differences in technologies influenced the strength of relationship to LVDF measures. A sensitivity analysis was completed by repeating the analysis but excluding any studies reported as “poor”. A further sensitivity analysis was completed by excluding any study that included participants older than the American Academy of Paediatrics definition of adolescence (11–21 years).^25^

The *r* ^2^ and adjusted *r* ^2^ were reported for each model, and effect sizes, SE, 95% confidence intervals (CI), *z*‐statistic, and *P*‐value were reported for each independent variable in the models. *P* < 0.05 was considered statistically significant.

## 6 RESULTS

### 6.1 Study characteristics

**FIGURE 2:**
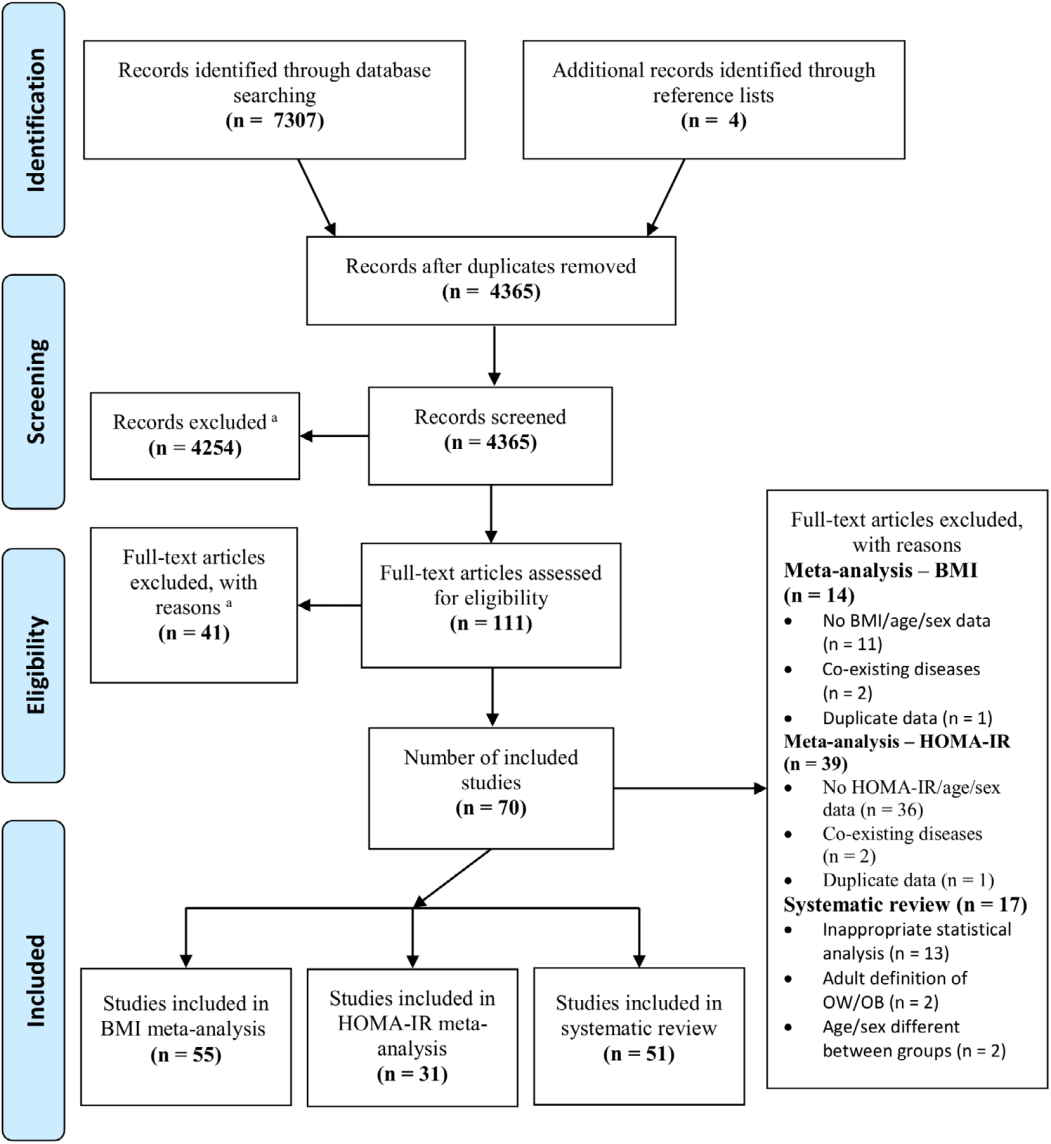
Flow diagram of study identification, screening, eligibility and inclusion/exclusion. Echo indicates echocardiography; HOMA‐IR, Homeostatic Model Assessment of Insulin Resistance; n, number of studies; OB, obese; OW, overweight. ^a^Exclusion criteria and reasons can be found in the Supporting Information

Searches identified 7311 studies. After duplicate removal, 4365 were screened and 4254 were excluded, leaving 111 full‐text articles to be assessed (Figure 2). A total of 70 studies (Table S1; sample size = 20–799; representing 9983 participants) were eligible, with 51 studies in the systematic review, 55 studies in the BMI meta‐analysis (sample size = 20–650; representing 6782 participants), and 31 studies in the HOMA‐IR meta‐analysis (Figure 2 and Table S1; sample size = 20–650; representing 3878 participants). All included studies assessed LVDF by echocardiography.

**FIGURE 3:**
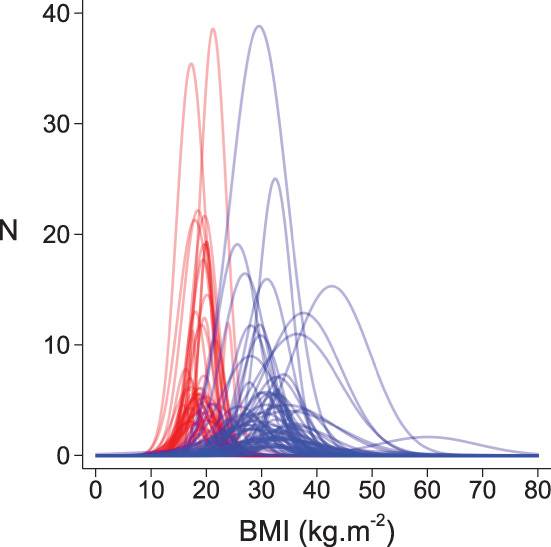
Distribution of body mass index (BMI) in control (red) and overweight/obese (blue) groups included in the meta‐analysis. Groups were defined as per the definitions in individual studies. A normal distribution curve was generated using the reported sample size (*N*), mean BMI, and BMI standard deviation. Significant overlap of BMI distributions between groups and marked variability of distributions within groups highlights that it was not possible to perform traditional group‐based meta‐analysis reliably

One study that assessed LVDF by CMR was identified but was excluded from the systematic review due to different ages of participants between groups. The number of studies and number of participants for each LVDF measure are reported in Table 1. Of these, 6 studies were scored as good, 48 as fair, and 16 as poor for quality and risk of bias (Table S1). Mean age, percentage of males, and mean BMI ranged from 8.9 to 18.4 years‐of‐age, 0–100%, and 15.8–60.0 kg/m^2^, respectively. There was marked heterogeneity in group‐definitions, with >20 definitions identified for groups with OW/Ob groups and > 20 for control groups (Table S1). Furthermore, there was marked overlap of BMI between control and OW/Ob groups and marked dispersion of BMI within groups across studies, presenting a major challenge to the reliable use of conventional group‐based meta‐analysis (Figure 3).

**TABLE 1:** Total number of studies and participants available for meta‐analysis with BMI for each LVDF measure

Measure	Total number of studies	Number of studies in meta‐analysis	Total number of participants	Number of participants in meta‐analysis
E wave	38	33	4056	3660
A wave	39	34	4200	3754
*E*/*A*	52	42	7795	5668
DT	19	14	2203	1383
IVRT	24	17	4190	1890
*e*′	41	33	4464	3491
*a*′	28	22	3308	2534
*E*/*e*′	38	30	5048	4075
*e*′/*a*′	23	18	3874	2782

Abbreviations: A wave, late mitral inflow peak velocity; *a*′, late diastolic tissue peak velocity; BMI, body mass index; DT, E wave deceleration time; E wave, early mitral inflow peak velocity; *e*′, early diastolic tissue peak velocity; *E*/*A*, E wave/A wave ratio; *E*/*e*′, E wave/*e*′ ratio; *e*′/*a*′, *e*′/*a*′ ratio; IVRT, isovolumic relaxation time.

### 6.2 Objective 1—The association of OW/Ob with measures of LVDF

Objective 1 was to determine the association of OW/Ob with LVDF. This was done by meta‐analysis and by systematic review. A small subset of papers addressed this question directly as a study outcome and the findings of these are summarized in Table S2.

#### 6.2.1 Meta‐analysis

The associations of BMI with measures of LVDF, after adjustment for age and sex, are given in Tables 2 and S3. There was evidence of reduced myocardial motion indicated primarily by strong associations of septal early diastolic tissue peak velocity/late diastolic tissue peak velocity (*e*′/*a*′) ratios and septal *a*′ peak velocities with BMI. For example, a typical child/adolescent with a BMI of 35 might have a septal *e*′/*a*′ ratio of 2.3 and a septal *a*′ peak velocity of 6.6 cm/s, compared to 2.0 and 5.5 cm/s, respectively, for controls with a BMI of 20. BMI was associated with all other measures of LVDF, apart from early mitral inflow peak velocity deceleration time (DT). Independent associations of LVDF with age are reported in Table S3. There were no independent effects of sex distribution in the studies.

**TABLE 2:** Associations of BMI with each left ventricular diastolic function measure, ranked by strength of association (*r*)

Measure (units per 10 point change in BMI)	Number of studies	References	Correlation coefficient (*r*)	*b*	95% CI	Fisher's *z*‐test
*e*′/*a*′ sep (1/kg/m^2^)	13	19, 22, 26, 27, 28, 29, 30, 31, 32, 33, 34, 35, 36	−0.689	−0.240	**−0.299, −0.180**	0.000
*a*′ sep ((cm/s)/kg/m^2^)	16	19, 22, 26, 27, 28, 29, 30, 31, 32, 33, 34, 35, 36, 37, 38, 39	0.621	0.743	**0.522, 0.965**	0.239
*e*′/*a*′ lat (1/kg/m^2^)	12	19, 27, 28, 29, 30, 31, 32, 33, 35, 40, 41, 42	−0.593	−0.366	**−0.525, −0.208**	0.318
*a*′ lat ((cm/s)/kg/m^2^)	14	19, 27, 28, 29, 30, 31, 32, 33, 35, 38, 39, 40, 41, 42	0.432	0.877	**0.558, 1.195**	0.883
*E*/*e*′ sep (1/kg/m^2^)	16	19, 22, 26, 30, 32, 33, 36, 37, 38, 39, 43, 44, 45, 46, 47, 48	0.431	0.814	**0.593, 1.035**	0.902
*e*′ sep ((cm/s)/kg/m^2^)	19	19, 22, 26, 27, 28, 29, 30, 31, 32, 33, 34, 35, 36, 37, 38, 39, 46, 48, 49	−0.413	−0.747	**−1.057, −0.437**	1.012
*E*/*e*′ average (1/kg/m^2^)	16	19, 30, 32, 33, 35, 38, 39, 43, 44, 46, 47, 50, 51, 52, 53, 54, 55	0.387	0.666	**0.552, 0.781**	1.046
*a*′ average ((cm/s)/kg/m^2^)	14	19, 21, 27, 28, 29, 30, 31, 32, 33, 35, 38, 39, 50, 51	0.343	0.589	**0.255, 0.924**	1.176
*e*′/*a*′ average (1/kg/m^2^)	11	19, 27, 28, 29, 30, 31, 32, 33, 35, 54, 56	−0.306	−0.155	**−0.262, −0.048**	1.208
*e*′ average ((cm/s)/kg/m^2^)	20	19, 21, 27, 28, 29, 30, 31, 32, 33, 35, 38, 39, 46, 49, 50, 51, 52, 53, 55, 56	−0.294	−0.912	**−1.302, −0.522**	1.463
*e*′ lat ((cm/s)/kg/m^2^)	20	19, 27, 28, 29, 30, 31, 32, 33, 35, 38, 39, 40, 41, 42, 46, 49, 57, 58, 59, 60	−0.247	−1.161	**−1.571, −0.752**	1.649
*E*/*e*′ lat (1/kg/m^2^)	18	19, 21, 29, 30, 31, 32, 33, 35, 38, 39, 42, 43, 44, 46, 47, 58, 59, 61	0.237	0.462	**0.336, 0.589**	1.645
IVRT (ms/kg/m^2^)	17	14, 24, 28, 29, 35, 40, 43, 46, 48, 52, 53, 57, 58, 59, 61, 62, 63	0.222	2.861	**0.961, 4.762**	1.718
A wave ((cm/s)/kg/m^2^)	34	12, 14, 15, 18, 19, 20, 21, 22, 23, 24, 25, 26, 27, 28, 29, 30, 31, 32, 33, 34, 35, 36, 37, 38, 39, 40, 41, 42, 43, 44, 45, 46, 47, 48	0.216	2.636	**1.660, 3.612**	1.933
E wave ((cm/s)/kg/m^2^)	33	12, 14, 15, 18, 19, 20, 21, 22, 23, 24, 25, 26, 27, 28, 29, 30, 31, 32, 33, 34, 35, 36, 37, 38, 39, 40, 41, 42, 43, 44, 45, 46, 47	0.178	1.774	**0.355, 3.193**	2.104^a^
*E*/*A* (1/kg/m^2^)	42	12, 14, 15, 18, 19, 20, 21, 22, 23, 24, 25, 26, 27, 28, 29, 30, 32, 34, 35, 36, 37, 38, 40, 41, 42, 43, 44, 45, 46, 47, 48, 49, 50, 51, 52, 53, 54, 55, 56, 57, 58, 59, 60	−0.147	−0.056	**−0.080, −0.032**	2.275^a^
DT (ms/kg/m^2^)	14	14, 15, 21, 24, 26, 29, 34, 35, 36, 40, 44, 53, 58, 61	−0.005	−0.220	−8.987, 8.546	2.408^a^

*Note*: Associations that were statistically significant (*P* < 0.05) are represented by bold 95% confidence intervals (CI). Fisher's *z*‐test, which accounts for sample size, was used to compare the strength of the correlation coefficients (*r*) with the strongest association, septal (sep) *e*′/*a*′, as a reference. Larger values of Fisher's *z* indicate that correlation coefficients are more likely to be statistically different (less strongly associated) with respect to the reference association. Those that were significantly different (*P* < 0.05) are marked with an ^a^. Tissue Doppler imaging (TDI) measures are reported as an average of recordings from the septal and lateral wall (lat) of the left ventricle, and individually as sep and lat.

Abbreviations: A wave, late mitral inflow peak velocity; *a*′, late diastolic tissue peak velocity; *b*, unstandardized regression coefficient; BMI, body mass index; DT, E wave deceleration time; E wave, early mitral inflow peak velocity; *e*′, early diastolic tissue peak velocity; *E*/*A*, E wave/A wave ratio; *E*/*e*′, E wave/*e*′ ratio; *e*′/*a*′, *e*′/*a*′ ratio; IVRT, isovolumic relaxation time.

Findings were not altered meaningfully by attempts to normalize non‐normal distributions, although there was a marginal improvement for associations of BMIz with *a*′ (*r* = 0.441) and with DT (*r* = 0.158). The latter did not become statistically significant after such normalization. Repetition of the analyses using robust *z*‐scores or with adjustment for the type of echocardiography machine used in each study made no meaningful difference to the results (not shown). Sensitivity analyses to exclude poor quality studies also made no meaningful difference to the results. Sensitivity analyses to exclude studies with adolescents aged >21 years made no overall meaningful difference to the results. However, averaged *e*′/*a*′ ratios were no longer significantly associated with BMI (Table S3).

#### 6.2.2 Systematic review

**FIGURE 4:**
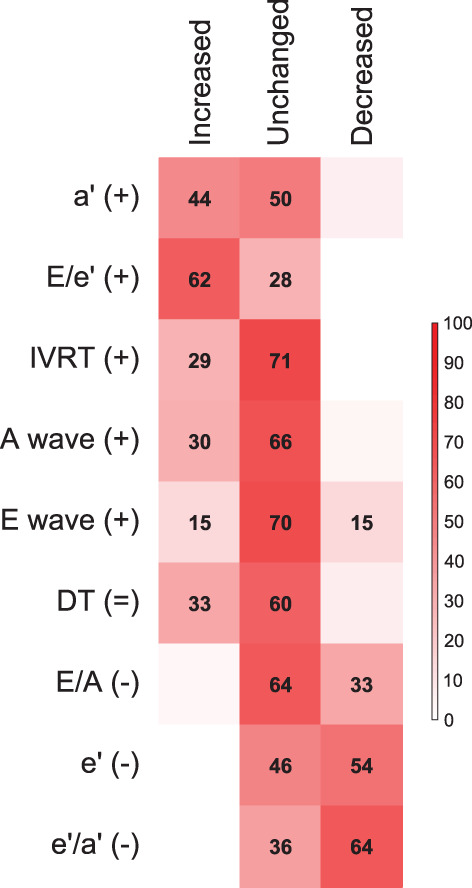
Percentage of studies included in the qualitative analysis reporting increased (+), unchanged (=), or decreased (−) measures of left ventricle diastolic function (LVDF) in children/adolescents with OW/Ob compared to controls. Darker red squares indicate a greater percentage of studies. Measures are ranked by the strength of association (*r*) from strongest positive to strongest negative as identified in the meta‐analysis. The directionality of greater percentages from top left to bottom right supports the meta‐analysis results. A wave indicates late mitral inflow peak velocity; *a*′, late diastolic tissue peak velocity; DT, E wave deceleration time; E wave, early mitral inflow peak velocity; *e*′, early diastolic tissue peak velocity; *E*/*A*, E wave/A wave ratio; *E*/*e*′, E wave/*e*′ ratio; *e*′/*a*′, *e*′/*a*′ ratio; IVRT, isovolumic relaxation time

Forty‐three studies eligible for systematic review reported matching LVDF measures to those included in the meta‐analysis (Figure 4).^19^, ^20^, ^21^, ^26^, ^28^, ^29^, ^33^, ^35^, ^37^, ^39^, ^40^, ^44^, ^46^, ^48^, ^49^, ^50^, ^52^, ^53^, ^54^, ^57^, ^58^, ^59^, ^61^, ^62^, ^63^, ^64^, ^65^, ^66^, ^67^, ^68^, ^69^, ^70^, ^71^, ^72^, ^73^, ^74^, ^75^, ^76^, ^77^, ^78^, ^79^, ^80^, ^81^ A full list of these results is given in Table S5.

### 6.3 Objective 2—Comparison of measures of LVDF

Standardized *r* coefficients are reported in Table 2 and S4. BMI was most strongly associated with septal *e*′/*a*′ ratio after adjustment for age and sex. Septal TDI measures were more strongly associated with BMI than lateral or averaged equivalents. Other TDI measures of LVDF, especially those including *a*′ peak velocities, were more strongly associated with BMI than conventional measures of mitral inflow peak velocities (e.g. early mitral inflow peak velocity [E wave]). Associations of BMI with E wave peak velocity, E wave/late mitral inflow peak velocity ratio (*E*/*A* ratio), and DT were significantly weaker than that of septal *e*′/*a*′ (Table 2), suggesting inferiority for early detection of reduced LVDF in childhood and adolescent OW/Ob.

### 6.4 Objective 3—The association of HOMA‐IR and other CMRFs with LVDF

#### 6.4.1 Meta‐analysis

A small subset of papers addressed the association between HOMA‐IR and LVDF directly as a study outcome and the findings of these are summarized in the Supporting Information.

Evidence of reduced LVDF with increasing levels of HOMA‐IR are reported in Table 3 and S6. The strongest association was with averaged E wave peak velocity/*e*′ peak velocity ratios (*E*/*e*′ ratio). Other TDI measures of LVDF and IVRT were more strongly associated with HOMA‐IR than conventional measures of mitral inflow velocities. E wave peak velocity, DT, septal *e*′ peak velocity, and lateral *a*′ peak velocity were not associated with HOMA‐IR. Associations of HOMA‐IR with measures of LVDF were not statistically different than that of averaged *E*/*e*′ ratios (Table 3). There were insufficient data on septal *E*/*e*′ ratios to be included in the meta‐analysis. Independent associations of LVDF with age and sex are reported in Table S6.

**TABLE 3:** Associations of HOMA‐IR with each left ventricular diastolic function measure, ranked by strength of association (*r*)

Measure (units per 1 point change in HOMA‐IR)	Number of studies	References	Correlation coefficient (*r*)	*b*	95% CI	Fisher's *z*‐test
*E*/*e*′ average	7	39, 46, 50, 51, 52, 78, 79	0.600	0.509	**0.296, 0.723**	0.000
IVRT (ms)	13	35, 41, 45, 46, 67, 68, 75, 77, 78, 79, 82, 83, 84	0.463	3.56	**1.310, 5.810**	0.376
*e*′/*a*′ sep	5	26, 28, 29, 31, 35	−0.412	−0.098	**−0.163, −0.033**	0.370
*a*′ sep (cm/s)	6	26, 28, 29, 31, 35, 39	0.402	0.387	**0.241, 0.534**	0.426
*e*′ average (cm/s)	12	28, 29, 31, 35, 39, 46, 49, 50, 51, 52, 78, 79	−0.332	−0.673	**−1.125, −0.220**	0.714
*e*′/*a*′ lat	7	28, 29, 31, 35, 41, 42, 83	−0.291	−0.094	**−0.132, −0.056**	0.725
*e*′ lat (cm/s)	12	28, 29, 31, 35, 39, 41, 42, 46, 49, 57, 59, 83	−0.247	−0.73	**−1.146, −0.313**	0.940
*a*′ average (cm/s)	8	28, 29, 31, 35, 39, 50, 51, 78	0.247	0.295	**0.187, 0.404**	0.828
*e*′/*a*′ average	5	28, 29, 31, 35, 78	−0.174	−0.056	**−0.103, −0.009**	0.839
*E*/*A*	24	26, 28, 29, 35, 39, 42, 45, 46, 49, 50, 52, 57, 59, 61, 62, 67, 68, 70, 71, 75, 77, 78, 79, 83, 84	−0.159	−0.035	**−0.059, −0.011**	1.308
A wave (cm/s)	16	26, 28, 29, 35, 39, 42, 49, 50, 52, 57, 59, 62, 68, 75, 78, 79	0.157	1.169	**0.605, 1.733**	1.238
*E*/*e*′ lat	9	29, 31, 35, 39, 42, 46, 59, 61, 83	0.156	0.161	**0.017, 0.306**	1.084
*a*′ lat (cm/s)	7	28, 29, 31, 35, 39, 41, 42	0.137	0.19	−0.044, 0.423	1.060
DT (ms)	14	39, 41, 42, 45, 46, 52, 59, 62, 68, 75, 78, 79, 83, 84	−0.103	−2.654	−7.007, 1.699	1.365
*e*′ sep (cm/s)	8	26, 28, 29, 31, 35, 39, 46, 49	−0.098	−0.132	−0.399, 0.136	1.203
E wave (cm/s)	16	26, 28, 29, 35, 39, 42, 49, 50, 52, 57, 59, 62, 68, 75, 78, 79	0.012	0.091	−1.004, 1.185	1.644
*E*/*e*′ sep^a^	^—^	^—^	^—^	^—^	^—^	^—^

*Note*: Associations that were statistically significant (*P* < 0.05) are represented by bold 95% confidence intervals (CI). Tissue Doppler imaging (TDI) measures are reported as an average of recordings from the septal and lateral wall (lat) of the left ventricle, and individually as sep and lat. Larger values of Fisher's *z* indicate that correlation coefficients are more likely to be statistically different (less strongly associated) with respect to the reference association.

Abbreviations: A wave, late mitral inflow peak velocity; *a*′, late diastolic tissue peak velocity; *b*, unstandardized regression coefficient; CI, confidence interval; DT, E wave deceleration time; E wave, early mitral inflow peak velocity; *e*′, early diastolic tissue peak velocity; *E*/*A*, E wave/A wave ratio; *E*/*e*′, E wave/*e*′ ratio; *e*′/*a*′, *e*′/*a*′ ratio; HOMA‐IR, homeostatic model assessment of insulin resistance; IVRT, isovolumic relaxation time.

^a^ There was an insufficient number of studies on septal *E*/*e*′ to be included in the analysis.

#### 6.4.2 Systematic review

Objective 3 was also to systematically review the association of other CMRFs with LVDF. Of the 51 studies included in the systematic review, all reported data on at least one CMRF but only 13 related these to LVDF.^26^, ^37^, ^39^, ^50^, ^51^, ^52^, ^54^, ^65^, ^66^, ^78^, ^79^, ^85^, ^86^ IR was the most common CMRF reported as associated with LVDF.^26^, ^50^, ^52^, ^65^, ^66^, ^79^, ^86^ A detailed description of the association between CMRFs and LVDF is provided in the Supporting Information.

## 7 DISCUSSION

We present the first meta‐analysis of studies that examined LVDF in children and adolescents with OW/Ob. We provide evidence that elevated BMI in the young is associated with reduced LVDF and show that the strongest associations are found when TDI indices of septal LV myocardial velocity are used. This could suggest that impaired LVDF in children and adolescents with OW/Ob begins in the septum. We also provide evidence that IR, as indicated by HOMA‐IR, is associated with reduced LVDF.

### 7.1 LVDF in childhood and adolescent OW/Ob

We conclude that elevated BMI in childhood and adolescence is adversely associated with all measures of LVDF, apart from DT, and support these findings with a systematic review. Primarily, we showed impaired longitudinal myocardial motion of the LV, identified by lower *e*′ peak velocities and *e*′/*a*′ ratios, and higher *a*′ peak velocities is associated with raised BMI.

LVDD begins with impaired relaxation and decreased ventricular “suction” in early filling, advancing to increased LV stiffness and elevated LAP, then high LAP and a non‐compliant LV, before becoming irreversible. In advanced LVDD, LA size increases markedly and symptoms of diastolic HF appear. In our analysis, we identified reduced myocardial motion in early diastole (*e*′) in those with greater BMI. As *e*′ is inversely related to the time constant of LV relaxation, tau (*τ*), our results likely represent a gradual reduction in myocardial relaxation with increasing adiposity.^87^ To overcome any abnormalities in early relaxation and maintain normal LV end diastolic volume, a more forceful ‘atrial kick’ is required, which can be identified by greater *a*′ peak velocities. Our results confirmed this, showing increased *a*′ peak velocities with greater BMI. Although these results do not represent large differences in LVDF, such LV motion abnormalities probably represent the early stage of LVDD and may, therefore, be useful for identifying those most at risk of future cardiac events. In support of this, young adults in the CARDIA study, aged 23–25 years at the time of echocardiography, with abnormal LVDF (defined by an *E*/*A* ratio < 1.3 and one marker of abnormal cardiac morphology) were 1.8 times more likely to have a clinical CVD event over 20 years of follow‐up.^88^

### 7.2 Measures with strongest relationship to BMI

We were able to compare the strength of the associations of BMI with different LVDF measures, yielding insight into which measurements are most useful for early detection of impaired LVDF in this context. Myocardial tissue peak velocities assessed by TDI were the LVDF measures that were most strongly associated with BMI. Of these, the strongest association was with the *e*′/*a*′ ratio, which is in accord with a study of children with Type 1 diabetes (T1D).^89^ These results also coincide with earlier studies of LVDF that identified the *e*′/*a*′ ratio as the best marker of early longitudinal compliance abnormalities.^7^

Of these two components of LVDF, *a*′ was more strongly associated with BMI than *e*′ in our analysis. The stronger relationship with *a*′ may be explained by this measure being less influenced by volume overload, which is typically seen in obesity.^5^ We therefore suggest that measures of both *a*′ and *e*′/*a*′ should be considered the best markers to identify early impairments of LVDF in children and adolescents with obesity, particularly in the septum.

We found, in general, that TDI peak velocities assessed at the septal mitral annulus were more strongly associated with BMI than the lateral equivalent or their average. Stronger associations of BMI with septal TDI measures may reflect preferential remodelling of the septum prior to similar changes in the lateral myocardium.^59^ As myocardial hypertrophy leads to reduced compliance of the myocardium and worse LVDF,^90^ earlier septal remodelling may explain our findings. It should also be noted that lateral TDI measures are technically more difficult to obtain reliably, particularly in children with obesity. We suggest that clinicians should focus on septal TDI measures when screening for impaired LVDF in children with obesity, while lateral and averaged measures can be used to supplement these, if necessary.

The *E*/*A* ratio has traditionally played a central role in paediatric clinical practice for the assessment of LVDF and we confirm this with 52 of the 70 studies reporting *E*/*A* ratios. However, this measure is more influenced by ventricular loading conditions (fluid volume status) than TDI equivalents and, unlike TDI measures, summarizes global ventricular compliance, rather than, for example, longitudinal septal compliance. This probably makes it less sensitive to the earliest pathological changes in LVDF, which may be localized, compensated for by other elements of diastolic function elsewhere in the ventricle, and preferentially affecting particular myocardial fibre groups / directions in the heart. In our analysis, the *E*/*A* ratio was only weakly associated with BMI compared to other LVDF measures, supporting this suggestion and the findings of earlier studies.^91^ Therefore, we recommend that the *E*/*A* ratio should not be used alone to assess LVDF in paediatric OW/Ob.

### 7.3 Cardiometabolic health and LVDF

IR is associated with cardiovascular events,^13^ and contributes to progressive declines in LVDF, independent of confounders.^14^ The pathogenesis of IR associated with obesity is described elsewhere in detail,^92^, ^93^ but can include abnormal adipokine/cytokine production, systemic inflammation, mitochondrial dysfunction, lipotoxicity, oxidative stress, hypoxia, and hyperinsulinemia, which likely contribute to reduced myocardial compliance and impaired LVDF. We identified that HOMA‐IR is associated with numerous measures of LVDF in young people with OW/Ob and confirm this with the findings in our systematic review. Averaged *E*/*e*′ ratios and other TDI measures were more strongly associated with HOMA‐IR than typical mitral inflow velocities, reflecting the pattern of associations with BMI, but Fisher's *z*‐test was unable to demonstrate statistically significant differences in the strength of these associations, probably due to the smaller dataset. Although relationships of LVDF measures with BMI and with HOMA‐IR were broadly similar, IVRT was more strongly related to HOMA‐IR than it was to BMI. It is unclear why this might be, but it could be due to the relative lack of data on HOMA‐IR.

The systematic review (Supporting Information) identified some studies reporting worse LVDF in children/adolescents with obesity and poor cardiometabolic health compared to normal cardiometabolic health counterparts. Although poor metabolic health is more likely in individuals with obesity, its adverse effects on LVDF can be demonstrated regardless of weight class in adults.^94^ Future work should examine the mechanisms and consequences of both obesity and cardiometabolic health on LVDF in younger people.

### 7.4 Standardization of group definitions

Previous studies do not report a standardized measure of obesity that can be compared between studies. We confirm this with >20 definitions identified for both groups with OW/Ob and control groups. When country‐specific BMI *z*‐scores are reported, authors should also report a standardized, global BMI *z*‐score using common, easily accessible tools such as the World Health Organization (WHO) 2007 BMI *z*‐scores.^95^ This would allow for the direct comparison between studies and aid future attempts to statistically synthesize data on childhood and adolescent OW/Ob.

### 7.5 Study strengths and limitations

This work has a number of strengths and potential limitations. Some studies were rejected from the meta‐analyses due to insufficient reporting of BMI, HOMA‐IR, age and sex. Nevertheless, the results of the systematic review, which was more inclusive, broadly reflected the findings of the meta‐analyses, supporting our conclusions. We limited the impact of group selection bias in our meta‐analyses by including the pairwise differences between groups within a study as a unique level in the multilevel model but, importantly, ignoring the authors' group definitions in favour of a continuous analysis, using the reported mean BMI instead. Linear regression analyses can be unduly influenced by non‐normal distributions, but our results did not differ meaningfully after the normalization of distributions and with repeat analysis using robust *z*‐scores.

Although some studies reported BMI *z*‐scores, it was not possible to construct an analysis using BMI *z*‐scores due to the heterogeneity in the normative data used to calculate BMI *z*‐scores. Any study that defined groups using BMI *z*‐scores and that was not included in the meta‐analyses was included in the systematic review.

The marked heterogeneity in definitions of normal weight and OW/Ob required an analytical approach that ignored these group definitions. As a consequence, it was also not possible to analyse whether individual study findings were distributed evenly around the mean effect size and thus less likely to be subject to publication bias. Thus, our study does not address possible publication bias statistically. However, it should be noted that only data on unpublished studies truly allow for publication bias to be determined and these are not available in this context.

## 8 CONCLUSIONS

This review provides the first evidence by meta‐analysis that childhood adiposity, as indexed by BMI, is associated with worse LVDF. We demonstrate that increased BMI is most strongly associated with septal *e*′/*a*′ ratios. These findings should aid the development of paediatric guidelines for the assessment of LVDF, by highlighting the most sensitive measures for early detection of LVDF in children with OW/Ob.

We also demonstrate that increased levels of HOMA‐IR are associated with LVDF, which may be particularly useful for understanding the early pathogenesis of LVDD. Further work should address the longitudinal consequences of childhood obesity and cardiometabolic dysfunction with LVDF.

## CONFLICT OF INTEREST

The authors have no conflicts of interest relevant to this article to disclose.

## Supporting information


**Appendix S1** Supporting information
