# Supplementary material for: The effect of overweight/obesity on diastolic function in children and adolescents: A meta‐analysis
Source: Clin Obes. 2021 Jul 18;11(5):e12476. doi: 10.1111/cob.12476 (PMC8767098; doi:10.1111/cob.12476)
Supplement: Supplementary file 1 — Appendix S1 Supporting information [file COB-11-e12476-s001.docx]

**SUPPORTING INFORMATION**

**The Effect of Overweight/Obesity on Diastolic Function in Children and Adolescents: A Meta-analysis**

*Samuel Burden^1,2^, Benjamin Weedon ^1,2^, Luke Whaymand ^1^, Josefien Rademaker^2,3^, Helen Dawes^1,2,4^, Alexander Jones^2^**

^1^Centre for Movement, Occupational and Rehabilitation Sciences, Oxford Institute of Nursing, Midwifery and Allied Health Research, Oxford Brookes University, UK

^2^Department of Paediatrics, University of Oxford, UK

^3^Leiden University Medical Centre, Netherlands

^4^Oxford Health NHS Foundation Trust, UK

*Corresponding Author: Dr. Alexander Jones

Department of Paediatrics, Level 2, Children’s Hospital, John Radcliffe, Headington, Oxford, Oxfordshire, OX3 9DU, UK

Email: alexander.jones@paediatrics.ox.ac.uk

**Contents**

[Supporting Methods 3](#_Toc70610384)

[Outcome Measures 3](#_Toc70610385)

[Combining Septal and Lateral Wall Tissue Doppler Imaging Measures 3](#_Toc70610386)

[Search Hedges 3](#_Toc70610387)

[Title and Abstract Inclusion/Exclusion 4](#_Toc70610388)

[Full-Text Inclusion/Exclusion Criteria 4](#_Toc70610389)

[Reporting of BMI, HOMA-IR, Age and Sex 5](#_Toc70610390)

[Transformation into Standard Units 5](#_Toc70610391)

[Robust z-score Calculation 6](#_Toc70610392)

[Fisher’s z-test 6](#_Toc70610393)

[Supporting Results 7](#_Toc70610394)

[Systematic review of pulmonary vein flow velocities and diastolic strain 7](#_Toc70610395)

[Systematic review of the association between CMRFs and LVDF 7](#_Toc70610396)

[Supporting Tables 9](#_Toc70610397)

[Study Quality Assessment Tools 49](#_Toc70610398)

[References 65](#_Toc70610399)

**Contents of Tables**

[**Table S1:** Study Characteristics 9](#_Toc70610400)

[**Table S2:** Study Specific Positive (↑), Negative (↓), or Non-Significant (↔) Associations of Measures Left Ventricular Diastolic Function with Measures of Adiposity 37](#_Toc70610401)

[**Table S3:** Association of Left Ventricular Diastolic Function Measures with BMI, Age and Sex 39](#_Toc70610402)

[**Table S4:** Increased (↑), Decreased, (↓), or Unchanged (↔) Left Ventricular Diastolic Function in Obese/Overweight Children and Adolescents – Results of Studies Included in the Qualitative Analysis 42](#_Toc70610403)

[**Table S5**: Association of Left Ventricular Diastolic Function Measures with HOMA-IR, Age and Sex 46](#_Toc70610404)

# Supporting Methods

## Outcome Measures

The primary outcome measures were: early mitral inflow peak velocity (E wave); late mitral inflow peak velocity (A wave); E wave/A wave ratio (E/A); E wave deceleration time (DT); isovolumic relaxation time (IVRT); early diastolic tissue peak velocity (e’); late diastolic tissue peak velocity (a’); E wave/e’ ratio (E/e’); e’/a’ ratio (e’/a’).

Cardiometabolic risk factors included, but were not limited to: systolic and diastolic blood pressure; total triglycerides; total cholesterol; low-density lipoproteins; high-density lipoproteins; blood glucose; blood insulin; homeostatic model assessment of insulin resistance (HOMA-IR); haemoglobin A1C (HbA1c); and C-reactive protein (CRP).

## Combining Septal and Lateral Wall Tissue Doppler Imaging Measures

These formulas have been taken from the Cochrane handbook.^1^

|  | **Septal** | **Lateral** | **Combined Groups** |
| --- | --- | --- | --- |
| Sample Size | *N_1_* | *N_2_* | $N_{1} \times N_{2}$ |
| Mean | *M_1_* | *M_2_* | $\frac{N_{1}M_{1}+ N_{2}M_{2}}{N_{1}+ N_{2}}$ |
| SD | *SD_1_* | *SD_2_* | $\surd\frac{\left( N_{1}-1 \right){{SD}_{1}}^{2}+\left( N_{2}-1 \right){{SD}_{2}}^{2}+ \frac{N_{1}N_{2}}{N_{1}+ N_{2}}\left( {M_{1}}^{2}+ {M_{2}}^{2}-2M_{1}M_{2} \right)}{N_{1}+ N_{2}-1}$ |

## Search Hedges

### **PubMed.gov**

Date Searched: 11/07/2020

Number of results: 1237

Full Search Strategy:

((cardi*[title/abstract] OR heart[title/abstract] OR diastol*[title/abstract] OR ventric*[title/abstract]) AND (function*[title/abstract] OR dysfunct*[title/abstract] OR fail*[title/abstract])) AND (child*[title] OR adolesc*[title] OR teen*[title] OR youth*[title] OR paed*[title] OR pedia*[title]) AND (obes*[title/abstract] OR overweight[title/abstract] OR over-weight[title/abstract] OR adipos*[title/abstract] OR obesity[MeSH Terms])

### **Cumulative Index to Nursing and Allied Health Literature (CINAHL)**

Date Searched: 11/07/2020

Number of results: 489

Full Search Strategy:

S1 – TX cardi* OR heart OR diastol* OR ventric*

S2 – TX function* OR dysfunct* OR fail*

S3 – TI child* OR adolesc* OR teen* OR youth* paed* OR pedia*

S4 – TX obes* OR overweight OR over-weight OR adipos*

S5 – S1 AND S2 AND S3 AND S4

### **Cochrane Central Register of Controlled Trials, and ClinicalTrials.gov**

Date Searched: 11/07/2020

Number of results: 245

Full Search Strategy:

S1 – (child* OR adolesc* OR teen* OR youth* OR paed* OR pedia*):ti

S2 – (cardi* OR heart OR diastol* OR ventric* ):ti,ab,kw

S3 – (function* OR dysfunct* OR fail*):ti,ab,kw

S4 – (obes* OR overweight OR over-weight OR adipos*):ti,ab,kw

S5 – (obes* OR overweight OR over-weight OR adipos*):ti,ab,kw

S6 – S4 or S5

S7 – S1 AND S2 AND S3 AND S6

### **Embase**

Date Searched: 11/07/2020

Number of results: 3267

Full Search Strategy:

((cardi* or heart or diastole* or ventric*) AND (function* or dysfunc* or fail*) AND (obes* or overweight or over-weight or adipos*)).mp AND (child* or adolesc* or teen* or youth* or paed* or pedia*).m_titl

### **Web of Science**

Date Searched: 11/07/2020

Number of results: 2069

Full Search Strategy:

TITLE: (child* OR adolesc* OR teen* OR youth* OR paed* OR pedia*) AND TOPIC: (((cardi* OR heart OR diastol* OR ventric*) AND (function* OR dysfunct* OR fail*) ) AND (obes* OR overweight OR over-weight OR adipos*) )

## Title and Abstract Inclusion/Exclusion

Exclude if there is no mention of;

- **Child, children, adolescent, youth, young people, teenager** *etc*.
- **Cardiovascular system**
  - Include if **echocardiography** (or **echo, strain, Doppler, tissue Doppler** **imaging (TDI), pulsed wave Doppler, speckle tracking, myocardial-tracking, or similar**) mentioned
  - Include if **cardiovascular magnetic resonance** imaging (**CMR, MRI**) mentioned
  - Include if mention “**organ health**”, **“organ damage”** or similar phrases
  - Include if **cardiopulmonary** mentioned
  - Include if **right ventricle** mentioned
  - Include if **left atria** mentioned
  - Exclude if no link to the heart (e.g. “vascular stiffness” or “endothelial function”)
- **Overweight, obesity, BMI, adiposity, waist circumference** *etc*.
  - Include if **diabetes** mentioned
  - Include if **metabolic syndrome (MetS)** mentioned

Exclude if mentioned;

- Congenital heart disease, cancer or any other disease/illness not related to obesity.

## Full-Text Inclusion/Exclusion Criteria

### **Meta-analysis – BMI**

To be included in the meta-analysis studies must include: (1) evaluation of overweight /obese children and adolescents (≤ 24 years of age) that report BMI, age and sex; (2) reported correct methods to assess left ventricle diastolic function (LVDF).

Exclusion criteria were as follows: (1) study of other illness/disease; (2) failure to report either BMI, age and/or sex; (3) incorrect methods for assessing LVDF (4) substantial missing data; (5) no clear description of inclusion/exclusion criteria; and (6) suspected false reporting (e.g. reported standard deviations of 0).

### **Meta-analysis – HOMA-IR**

To be included in the meta-analysis studies must include: (1) evaluation of children/adolescents with overweight/obesity (≤ 24 years of age) that report HOMA-IR, age and sex; (2) reported correct methods to assess left ventricle diastolic function (LVDF).

Exclusion criteria were as follows: (1) study of other illness/disease; (2) failure to report either HOMA-IR, age and/or sex; (3) incorrect methods for assessing LVDF (4) substantial missing data; (5) no clear description of inclusion/exclusion criteria; and (6) suspected false reporting (e.g. reported standard deviations of 0).

### **Systematic Review**

Inclusion criteria were as follows: (1) evaluation of children and adolescents (≤ 24 years of age) with a valid definition of childhood overweight/obesity, (2) report correct measures of LVDF, (3) concurrent evaluation with normal-weight or non-obese controls, and/or conducted analysis that studied the effect of cardiometabolic risk factors (CMRFs).

Exclusion criteria were as follows: (1) study of other illness/disease (e.g. Type 2 diabetes); (2) adult definition of obesity (if studies included subjects < 18 years of age); (3) failure to define obesity; (4) significantly different age or sex between groups; (5) incorrect methods for measuring LVDF (e.g. cursor not placed on the mitral valve tips for the assessment of mitral inflow velocities); (6) biased recruitment of subjects (e.g. recruitment of controls from relatives of study staff); (7) no comparison control group; (8) substantial missing data with no explanation as to why it is missing; (9) inappropriate statistical analysis (e.g. no tests for normality); (10) no clear description of inclusion/exclusion criteria; and (11) suspected false reporting (e.g. reported standard deviations of 0).

### **Full-Text Articles Excluded – Reasons**

Forty-one full-text articles were excluded due to:

- Unsuitable for both quantitative and qualitative analysis (n = 13)^2-14^
- Study of other illness/disease (e.g. Type 2 diabetes) without normal overweight/obese (n = 8)^15-22^
- Apparent duplicate data (n = 4)^23-26^
- Reported results inconsistent/missing data (n = 4)^27-30^
- Suspected false reporting (n = 3)^31-33^
- Too old (>24 years of age) (n = 3)^34-36^
- Incorrect method for assessing LVDF (n = 2)^37,38^
- No inclusion/exclusion criteria (n = 2)^39,40^
- Data in format that cannot be reviewed (n = 1)^41^
- No overweight or obese (n = 1)^42^

## Reporting of BMI, HOMA-IR, Age and Sex

To be included in the meta-analysis, studies had to report measures on BMI/HOMA-IR, age and sex. Where studies did not report age or sex for the control group but included a statement that controls were matched to the obese group, the control group was assumed to have the same age and sex distribution. Where studies did not report sex for either group but included a statement that groups were matched, then groups were assumed to have a 50/50 distribution of male and females.

## Transformation into Standard Units

### **Median and Interquartile Range**

The methods provided by Shi *et al* were used to calculate the mean and SD from studies reporting median and interquartile range (IQR).^43^ As it has been reported that this SD estimator may be sensitive to the skewness of the data (as is commonly the reason for reporting median (IQR)) a sensitivity analysis was completed to understand whether these transformations affected the outcomes. Results were not altered after the sensitivity analysis.

### Mean and Confidence Intervals

The following formula has been taken from the Cochrane handbook.^1^

$$SD= \frac{\sqrt{N} \times\left( upper limit-lower limit \right)}{2 \times t}$$

The *t* value for a 95% confidence interval can easily be obtained in Excel by typing **=tinv(1-0.95,*N*-1)** where *N* is the study sample size.^1^

## Robust z-score Calculation

Robust z-scores were calculated using the median (x̃) and the median absolute deviation (MAD) for each variable:

$$robust z-score =\frac{X_{i}-x̃}{MAD \times1.4826}$$

The MAD is calculated as follows and is defined as the median of the absolute deviation from the median of the data points:

$$MAD={median}_{i}(\left| X_{i}-median X_{1\ldots n} \right|)$$

## Fisher’s z-test

Comparison of the strength of associations between standardized correlation coefficients (r) were calculated with the following formula and the difference (in terms of z-score) was read off from a two-sided z-table to provide a p-value:

$$Difference= \frac{|r|-{|r}_{i}|}{\sqrt{\left( N-3 \right)^{-1}+{(N_{i}-3)}^{-1}}}$$

The largest r coefficient was used for comparison to other r coefficients (r_i_). Statistical significance was set at p-value < 0.05.

# Supporting Results

## Systematic review of pulmonary vein flow velocities and diastolic strain

Similar measures to conventional transmitral flow velocities are used to assess left atrial pressure. These are systolic pulmonary vein peak velocity (S wave), diastolic pulmonary vein peak velocity (D wave), and the S wave/D wave ratio (S/D ratio).

Four studies provided data on S wave.^44-47^ All four studies reported no difference in S wave velocities in the overweight/obesity group. However, three studies reported a trend towards increased values in overweight/obesity group (P < 0.1). Four studies provided data on D wave.^44-47^ Two studies reported reduced D wave velocities in the overweight/obesity group and two studies reported no difference. One study provided data on S/D ratio with no reported difference in the overweight/obesity group.^45^ Further, work is needed to understand the utility of these measures of in children/adolescents with obesity.

Six studies reported data on diastolic strain or strain rate (SR) via speckle-tracking, markers of myocardial motion.^48-53^ These features move with the myocardium and measurement of that motion does not rely on alignment of the motion vector with the ultrasound beam as Doppler techniques do and may, therefore, be less prone to the limitations of other Doppler techniques.^54^ Four studies reported reduced SR in early diastole (SR_E_),^48,49,52,53^ whereas one study reported no difference.^51^ One study reported increased SR in late diastole (SR_A_) in the overweight/obesity group,^52^ whereas three studies reported no difference.^48,49,53^ Three studies reported reduced longitudinal SR.^48-50^ Measures of SR_E_/SR_A_ were reduced in the overweight/obesity group in one study^52^ and unaltered in another.^51^ These measures hold promise and future studies should try to analyse myocardial motion by tissue Doppler imaging and by speckle-tracking.

## Systematic review of the association between CMRFs and LVDF

IR was the most common CMRF reported as associated with LVDF, including TDI measures in five studies and SR_E_ in two studies.^46,48,53,55-58^ IR was assessed via the Homeostatic Model Assessment of Insulin Resistance (HOMA-IR) in all studies. One study found no relationship with HOMA-IR but reported associations between fasting insulin levels and SR_E._^49^ Three studies grouped participants with obesity by the presence (or not) of IR using thresholds of HOMA-IR; two studies reported reduced LVDF in participants with obesity and IR^56,57^ and one reported no difference.^55^ Furthermore, three studies grouped participants with obesity via indirect measures of IR; three using dysglycemia^59,60^ and one by Type 2 diabetes.^61^ LVDF was impaired further in participants with obesity and poor cardiometabolic health compared to normal cardiometabolic obese equivalents.^59,61^ Dysglycemia was also associated with e’ in one study.^62^ Additionally, the inflammatory marker C-reactive protein was associated with LVDF in four of the studies reporting associations with IR.^46,56-58^

Four studies reported associations of blood pressure (BP) with LVDF.^55,61-63^ Increased BP was associated with LVDF in only one study after adjusting for confounders.^61^ Additionally, three studies grouped participants with obesity by the presence (or not) of hypertension;^64-66^ two studies grouped participants with obesity using 24h ambulatory BP monitoring and found no difference in LVDF in the hypertensive obesity group compared to the normotensive obesity group, while one study grouped participants with obesity by office BP and found a significant difference in LVDF between groups.^66^

When participants with obesity were grouped by metabolic health status, defined by the metabolic syndrome (MetS) or other similar clusters of risk factors, two studies associated poor metabolic health with reduced LVDF^55,67^ and one study found no such association.^68^ In another two studies, participants were grouped by the presence of MetS, irrespective of their obesity status. In these, E/A and DT were impaired in one study^69^ and no differences in LVDF were found in the other.^70^ Another study reported that an adverse metabolic risk score was associated with lower e’ velocity and higher E/e’ ratio, indicating reduced LVDF.^58^ In addition, the group with obesity and non-alcoholic fatty liver disease (NAFLD) had reduced LVDF compared to the group with obesity without NAFLD in two-out-of-three studies.^48,71,72^

Future work should examine the mechanisms and consequences of both obesity and cardiometabolic health on LVDF in young people.

# Supporting Tables

**Table S1:** Study Characteristics

| **Study (First author, year)** | **N** | **BMI (kg/m2 - unless specified) and group definitions** | **Age range and mean age (years - unless specified)** | **Sex (male %)** | **LVDF Measures Reported** | **Country** | **Study Type** | **Included meta analysis / systematic review (Yes/No)** | **HOMA-IR analysis (included, fasted state, equation used)** | **Quality score** |
| --- | --- | --- | --- | --- | --- | --- | --- | --- | --- | --- |
| **Ahmed, 2016 ^73^** | Total: 92  OB: 56 Con: 36 | OB: 30.17 ± 5.8 BMI z-score for age greater than 2 SDS (WHO)  Con: 16.37 ± 1.84 Non-OB | 5-15  OB: 9.13 ± 2.8 Con: nr - age matched to OB | OB: 51.2 Con: nr - sex matched to OB | e', a', e'/a' | Egypt | cross-sectional case-control, single centre | Yes/Yes | Yes  Unclear  [fasting insulin (µU/L) × fasting glucose (nmol/L)]/22.5 | fair |
| **Akcaboy, 2016 ^64^** | Total: 83  OB: 27 Con: 21 Other: 35 | OB: 32.6 ± 3.3 BMI ≥ 95th percentile for age and sex (country specific - Turkey), OB normotensive  Con: 20.5 ± 2.6 cd  Other: 33.1 ± 4.7  OB hypertensive | 13-18  OB: 14.7 ± 1.5 Con: 15.4 ± 1.7 Other: 14.9 ± 1.4 | OB: 40.7 Con: 52.4 Other: 45.7 | E/A, E/e' | Turkey | cross-sectional case-control, single centre | Yes/Yes | Yes  Unclear  [fasting glucose (mg/dL) × fasting insulin (U/L)]/405 | fair |
| **Akyol, 2013 ^74^** | Total: 201  OW/OB: 94 Con: 63 Other: 44 | OW/OB: 34.9 ± 9.3 Unclear - BMI > 90th percentile for age and sex (cd), OW/OB without MetS  Con: 20.1 ± 1.3 cd  Other: 38.4 ± 4.0  OW/OB with MetS | 9-18  OW/OB: 13.9 ± 3.7 Con: 13.3 ± 4.3 Other: 13.3 ± 4.1 | OW/OB with and without MetS (grouped as one for sex): 52.2 Con: nr | IVRT, e', a', e'/a' | Turkey | cross-sectional case-control, single centre | Yes/No | Yes  Overnight fast  [fasting insulin (mIU/L) × fasting glucose (mmol/L)]/22.5 | poor |
| **Alkholy, 2016 ^75^** | Total: 122  OB: 82 Con: 40 | OB: 32.8 ± 4.6 BMI ≥ 95th percentile for age and sex (CDC)  Con: 18.7 ± 2.9 cd | 6-14  OB: 10.2 ± 2.8 Con: 10.6 ± 2.7 | OB: 57.3 Con: 55 | E wave, A wave, E/A, e' | Saudi Arabia | cross-sectional case-control, multi-centre | Yes/Yes | Yes  Unclear  [fasting insulin (µU/mL) × fasting glucose (mM)]/22.5 | fair |
| **Alp, 2014 ^76^** | Total: 650  OB: 500 Con: 150 | OB: 29.59 ± 5.14 BMI > 95th percentile for age and sex (country specific - Turkey)  Con: 18.53 ± 2.7 cd | 6-17  OB: 11.92 ± 3.65 Con: 11.97 ± 2.78 | OB: 53.2  Con 54.7 | E wave, A wave, E/A, e', a', E/e', e'/a' | Turkey | cross-sectional case-control, single centre | Yes/Yes | Yes  Unclear  [fasting insulin (IU/mL) × fasting glucose (mmol/L)]/22.5 | fair |
| **Aslan, 2019 ^68^** | Total: 300  OB: 98 Con: 102 Other: 100 | OB: 29.9 ± 3.6 BMI > 95th percentile for age and sex (WHO), OB without MetS  Con: 19.5 ± 2.3 cd  Other: 32.4 ± 3.5 OB with MetS | 12-17  OB: 13.9 ± 1.3 Con: 14.0 ± 1.3 Other: 14.0 ± 1.4 | OB: 40.8 Con: 49.0 Other: 40.0 | E wave, A wave, E/A, e', a' | Turkey | cross-sectional case-control, single centre | Yes/Yes | Yes  ≥12hr fast  [fasting insulin (mU/ml) × fasting glucose (mmol/L)]/22.5 | fair |
| **Battal, 2011 ^77^** | Total: 103  OB: 68 Con: 35 | OB: 31.0 ± 4.7 BMI ≥ 95th percentile for age and sex (cd)  Con: 18.8 ± 2.3 cd | 6-18  OB: 12 ± 1 Con: 12 ± 1 | OB: 35.3 Con: 40.0 | E wave, A wave, E/A, DT | Turkey | cross-sectional case-control, single centre | Yes/Yes | Yes  Unclear  [insulin (mU/L) × glucose (mmol/L)]/22.5 | poor |
| **Bjornstad, 2016 ^78^** | Total: 74  OB: 41 Con: 33 | OB: 2.0 ± 0.4 (BMI SDS) BMI > 95th percentile for age and sex (CDC)  Con: 0.1 ± 0.7 (BMI SDS) BMI > 5th percentile and < 85th percentile for age and sex | 12-19  OB: 14.4 ± 2.0 Con: 14.9 ± 2.1 | OB: 29 Con: 45 | E wave, A wave, E/A, DT, e', a', E/e' | USA | cross-sectional case-control, single centre | No/Yes | No | fair |
| **Boyraz, 2013 ^79^** | Total: 201  OB: 95 Con: 63 Other: 43 | OB: 34.7 ± 8.3 BMI-SDS between 1.65-2.49 (IOTF)  Con: 20.1 ± 1.3 cd  Other: 37.4 ± 4.1 BMI-SDS between 2.50-2.99, severely OB | 9-18  OB: 13.8 ± 3.9 Con: 13.3 ± 4.3 Other: 13.1 ± 4.1 | OB and other: 52.2 Con: nr - sex-matched | DT, IVRT, e', a', e'/a' | Turkey | cross-sectional case-control, single centre | Yes/No | Yes  Overnight fast  [fasting insulin (mIU/L) × fasting glucose (mmol/L)]/22.5 | fair |
| **Brar, 2019 ^59^** | Total: 78  OB: 25 Con: 39 Other: 14 | OB: 34.± 7 BMI ≥ 95th percentile for age and sex (cd)  Con: 19.2 ± 4 BMI < 85% percentile for age and sex  Other: 37.5 ± 8.8 OB with dysglycemia | 12-18  OB: 16 ± 1.9 Con: 15.8 ± 1.9 Other: 16 ± 1.9 | OB: 28 Con: 41 Other: 64.3 | E/A, E/e' | USA | cross-sectional case-control, single centre | Yes/Yes | No | fair |
| **Chinali, 2008 ^69^** | Total: 446  OW/OB: 111 Con: 335 | MetS: 37.0 ± 8.1 92.8% OB  No MetS: 25.9 ± 6.4  37.1% OB | ≤ 20  MetS: 17.6 ± 1.5 No MetS: 17.3 ± 1.4 | MetS: 44.1 No MetS: 48.1 | E/A, DT, IVRT, | USA | cross-sectional case-control, mutlti-centre | No/Yes | No | fair |
| **Corica, 2020 ^55^** | Total: 79  OW and OB: 59 Con: 20 | OW and OB: 2.2 ± 0.5 (BMI SDS) BMI z score > 1 (WHO)  Con: -0.3 ± 0.8 (BMI SDS) BMI z score ≤ 1 | 5-16  OW and OB: 9.8 ± 2.9 Con: 8.6 ± 2.9 | OW and OB: 55.9 Con: 50.0 | E/A, e', E/e' | Italy | cross-sectional case-control, single centre | No/Yes | No | good |
| **Cozzolino, 2015 ^56^** | Total: 98  OB: 33 Con: 30 Other: 35 | OB: 33 ± 9 BMI > 95th percentile for sex and age (cd)  Con: 24 ± 1 cd  Other: 33 ± 10 OB with insulin resistance | 10-16  OB: 12.7 ± 2.1 Con: 12.6 ± 2.0 Other: 12.9 ± 2.1 | OB: 42.9 Con: 50.0 Other: 45.9 | E wave, A wave, E/A, e', a', E/e', e'/a' | Italy | cross-sectional case-control, single centre | Yes/Yes | Yes  Overnight fast  [fasting insulin (pmol/L) x fasting glucose (mmol/L)]/22.5 | fair |
| **Dahiya, 2015 ^57^** | Total: 69  OW/OB: 35 Con: 34 | OW/OB: 2 ± 0.8 [BMI SDS (median ± IQR)] BMI equivalent to ≥ 25 at age 18 (IOTF)  Con: 0.03 ± 0.7 [BMI SDS (median ± IQR)], non-OW and OB | 10-19  OW/OB: 14.9 ± 2.3 Con: 15.3 ± 1.8 | OW/OB: 40.0 Con: 61.8 | A wave, E/A, DT, IVRT, e', E/e' | Australia | cross-sectional case-control, single centre | No/Yes | Yes  Overnight fast (10-12hrs)  [fasting insulin (U/mL x fasting glucose (mmol/L)/22.5] | fair |
| **Dhuper, 2011 ^80^** | Total: 343  OB: 213 Con: 130 | OB: 36.5 ± 0.53 BMI > 95th percentile for age and sex (CDC)  Con: 19.73 ± 0.21 BMI < 85th percentile for age and sex | nr  OB: 13.8 ± 0.2 Con: 13.8 ± 0.2 | OB: 50 Con: 61 | E/A, E/e' | USA | cross-sectional case-control, single centre | Yes/Yes | No | fair |
| **Di Bonito, 2009 ^81^** | Total: 195  OB: 165 Con: 30 | OB: 27 ± 4 BMI ≥ 95th percentile for age and sex (country specific - Italy)  Con: 17 ± 2 BMI < 85th percentile for age and sex | 6-16  OB: 10 ± 3 Con: 10 ± 3 | OB: 50.4 Con: 49.2 | E/A, IVRT, e'/a' | Italy | cross-sectional case-control, single centre | No/Yes | Yes  Unclear  [fasting insulin (U/L) × fasting glucose (mmol/L)]/22.5 | fair |
| **Di Bonito, 2010 ^70^** | Total: 799  OW/OB: 131 Con: 668 | MetS: 29 ± 5 99% OW or OB  No MetS: 24 ± 6 70% OW or OB | 6-16  OW/OB: 10 ± 3 Con: 10 ± 3 | OW/OB: 47 Con: 50 | E/A, IVRT, e'/a' | Italy | cross-sectional case-control, single centre | Yes/Yes | No | fair |
| **Di Salvo, 2008 ^82^** | Total: 320  OB: 160 Con: 160 | OB: 31 ± 4 BMI > 97th percentile for age and sex (IOTF)  Con: 18 ± 3 cd | 6-15  OB: 12 ± 3 Con: 12 ± 3 | OB: 44 Con: 44 | E wave, A wave, E/A, S wave, D wave, DT, IVRT, E/e' | Italy | cross-sectional case-control, single centre | Yes/No | Yes  Overnight fast (>12hrs)  Not reported | fair |
| **Dias, 2017 ^53^** | Total: 20  OB: 9 Con: 11 | OB: 31.7 ± 3.5 BMI > 95th percentile for age and sex (IOTF)  Con: 18.5 ± 2.3 BMI 5th-85th percentile for age and sex | 12-16  OB: 13.1 ± 1.0 Con: 13.5 ± 1.2 | OB: 33 Con: 73 | E/A, DT, IVRT, e', a', E/e', diastolic strain | Australia | cross-sectional case-control, single centre | Yes/Yes | Yes  Unclear  Not reported | fair |
| **Dusan, 2015 ^65^** | Total: 148  OB: 54 Con: 30 Other: 49 | OB: 30.0 ± 3.8 BMI > 95th percentile for age and sex (IOTF)  Con: 20.5 ± 3.0 cd  Other: 29.4 ± 3.2 OB with hypertension | 9-19  OB: 14.1 ± 2.3 Con: 15.0 ± 2.3 Other: 14.1 ± 2.0 | OB: 72.2 Con: 60.0 Other: 67.3 | E wave, A wave, E/A, DT, IVRT | Serbia | cross-sectional case-control, single centre | Yes/Yes | Yes  Overnight fast (12hrs)  [fasting glucose (mmol/L) x fasting insulin (mU/L)]/22.5 | fair |
| **Eklioglu, 2016 ^62^** | Total: 198  OB with pre-Diab: 81  OB without pre-Diab: 117 | OB with pre-Diab: 29.99 ± 9.03  BMI > 95th percentile for age and sex (CDC)  OB without pre-Diab: 28.05 ± 5.19  (see above) | 6-18  OB with pre-Diab: 11.84 ± 2.95  OB without pre-Diab: 11.88 ± 2.97 | OB with pre-Diab: 43.0  OB without pre-Diab: 45.3 | e’, a’, E/e’ | Turkey | cross-sectional case-control, single centre | Yes/No | Yes  Overnight fast  [fasting glucose x fasting insulin]/22.5 | poor |
| **El Saiedi, 2018 ^83^** | Total: 62  OB: 32 Con: 30 | OB: 30.6 ± 4.2 BMI ≥ 95th percentile for age and sex (country specific - Egypt)  Con: 19.1 ± 2.3 BMI 5th-85th percentile for age and sex | 6-19  OB: 10.8 ± 3.1 Con: 10.4 ± 3.0 | OB: 50.0 Con: 46.5 | E wave, A wave, E/A, e', a', E/e' | Egypt | cross-sectional case-control, single centre | Yes/Yes | No | fair |
| **Franssen, 2019 ^58^** | Total: 58  OB: 29 Con: 29 | OB: 31.6 ± 4.2 BMI > 95th percentile for age and sex (IOTF)  Con: 19.5 ± 2.4 cd | 11-17  OB: 13.4 ± 1.1 Con: 14.0 ± 1.5 | OB: 51.7 Con: 55.2 | E wave, A wave, E/A, DT, e', E/e' | Belgium | cross-sectional case-control, single centre | Yes/Yes | Yes  Fast >10hrs  [fasting glucose (mg/dl) × fasting insulin (μU/ml)]/405 | fair |
| **Ghanem, 2010 ^44^** | Total: 80  OB: 50 Con: 30 | OB: 28.4 ± 8.3 BMI > 95th percentile for age and sex (cd)  Con: 17.3 ± 2.8 cd | 6-18  OB: 11.2 ± 2.9 Con: 11.5 ± 3.4 | OB: 48.0  Con: 43.3 | E/A, S wave, D wave, DT, IVRT | Saudi Arabia | pre-post | Yes/Yes | No | poor |
| **Harris, 2012 ^84^** | Total: 116  OB: 61 Con: 55 | OB: 32.6 ± 4.4 BMI ≥ 95th percentile for age and sex (cd)  Con: 21.2 ± 4.7 BMI ≤ 95th percentile for age and sex | ≤ 18  OB: 13.8 ± 2.3 Con: 13.8 ± 4.0 | OB: 35.8 Con: 28.6 | E wave, A wave, E/A, e' | Canada | cross-sectional case-control, single centre | Yes/No | No | fair |
| **Hirschler, 2006 ^85^** | Total: 84  OB: 40 Con: 16 Severely OB: 28 | OB: 27.07 ± 4.19 BMI ≥ 95th percentile for age and sex (CDC)  Con: 15.83 ± 1.39 BMI < 85th percentile for age and sex  OW: 21.04 ± 2.40 BMI ≥ 85th percentile for age and sex | 14-21  OB: 16.9 ± 2.0 Con: 16.6 ± 1.9 OW: 16.9 ± 2.2 | OB: nr Con: nr OW: nr | E/A | Argentina | cross-sectional case-control, single centre | Yes/Yes | Yes  Fast (12-14hrs)  [fasting insulin (µ U/L) x fasting glucose (mmol/L)]/22.5 | fair |
| **Hui, 2019 ^86^** | Total: 88  OB: 44 Con: 44 | OB: 36.0 ± 6.6 BMI > 95th percentile for age and sex (CDC)  Con: 20.0 ± 3.3 BMI z-score ≤ 2 | 8-18  OB: 13.7 ± 2.9 Con: 13.6 ± 2.9 | OB: 47.7 Con: 47.7 | E wave, A wave, E/A, IVRT, e', E/e' | Canada | cross-sectional case-control, single centre | Yes/Yes | No | fair |
| **Ingul, 2010 ^87^** | Total: 20  OB: 10 Con: 10 | OB: 33.5 ± 4.3 BMI z score > 2 for age and sex (WHO)  Con: 20.4 ± 3.0 BMI z score ≤ 2 for age and sex | 13-16  OB: 14.8 ± 1.2 Con: 14.9 ± 1.3 | OB: 60 Con: 60 | E/A, DT, IVRT, e', E/e' | Norway | pre-post | Yes/Yes | No | fair |
| **Ingul, 2018 ^88^** | Total: 199  OB: 99 Con: 100 | OB: nr BMI > 95th percentile for age and sex (CDC)  Con: nr BMI 5th-85th percentile for age and sex | 7-16  OB: 12.0 ± 2.3 Con: 11.5 ± 2.4 | OB: 53.5 Con: 50 | E wave, A wave, E/A, DT, IVRT, e', a', E/e' | Australia and Norway | controlled intervention | No/Yes | No | fair |
| **Ippisch, 2008 ^89^** | Total: 38  OB: 38 | Morbidly OB: 60 ± 9 BMI > 99th percentile for age and sex (CDC) | 13-19  OB: 16 ± 1 | OB: 23.7 | E wave, A wave, E/A, e', a', E/e', e'/a' | USA | pre-post | Yes/Yes | No | good |
| **Kamal, 2012 ^90^** | Total: 120  OB: 70 Con: 50 | OB: 34.0 ± 3.8 BMI > 95th percentile for age and sex (cd)  Con: 21.6 ± 1.9 BMI < 95th percentile for age and sex | 12-15  OB: 14.0 ± 0.6 Con: 14.0 ± 0.9 | OB: 21.4 Con: 26.0 | e', e'/a' | Egypt | cross-sectional case-control, single centre | Yes/No | No | fair |
| **Karaagac, 2019 ^91^** | Total: 34  OB: 34 | OB: 28.6 ± 4.3 BMI ≥ 95th percentile for age and sex (CDC) | nr  OB: 10.8 ± 2.3 | OB: 47.1 | E wave, A wave, e', a', E/e' | Turkey | pre-post | Yes/Yes | No | poor |
| **Kibar, 2013 ^92^** | Total: 110  OB: 30  Con: 50  OW: 30 | OB: 32.9 ± 2.0  BMI ≥ 30 kg/m2  Con: 19.7 ± 1.6  BMI 17-24.9 kg/m2  OW: 27.2 ± 1.2  BMI 25-30 kg/m2 | 10-16.5  OB: 13.3 ± 2.0  Con: 13.2 ± 1.8  OW: 13.2 ± 2.1 | OB: 46.5  Con: 46.5  OW: 54.0 | IVRT, e’, a’, E/e’, e’/a’ | Turkey | cross-sectional case-control, single centre | Yes/No | Yes  Unclear  [fasting insulin (mIU/mL) x fasting glucose (mmol/L)]/22.5 | poor |
| **Kibar, 2015 ^52^** | Total: 110  OW/OB: 60  Con:50 | OW/OB: 30.1 ± 3.3  BMI > 95th percentile for age and sex (country specific – Turkey)  Con: 19.7 ± 1.6  cd | 10-16  OW/OB: 13.9 ± 2.3  Con: 13.2 ± 1.8 | OW/OB: 46.5  Con: 46.5 | Diastolic strain | Turkey | cross-sectional case-control, single centre | No/Yes | No | fair |
| **Kinik, 2006 ^93^** | Total: 58  OB: 30 Con: 28 | OB: 26.9 ± 4.2 BMI ≥ 95th percentile for age and sex (CDC)  Con: 17.1 ± 1.6 cd | 4-17  OB: 10.8 ± 3.3 Con: 10.2 ± 3.0 | OB: 43.4 Con: 50.0 | E wave, A wave, E/A, e', a', e'/a' | Turkey | cross-sectional case-control, single centre | Yes/No | No | poor |
| **Koopman, 2012 ^94^** | Total: 48  OW/OB: 21 Con: 27 | OW/OB: 32.4 ± 4.9 OW BMI 85th - 94th percentiles, OB BMI ≥ 95th percentile for age and sex (CDC). 20/21 were OB  Con: 18.9 ± 2.3 BMI < 85th percentile for age and sex | 10-18  OW/OB: 14.2 ± 2.0 Con: 13.9 ± 2.3 | OW/OB: 78.6 Con: 81.0 | E wave, A wave, e', a', E/e', diastolic strain | Canada | cross-sectional case-control, single centre | Yes/No | No | poor |
| **Korkmaz, 2016 ^95^** | Total: 158  OB: 79 Con: 79 | OB: 31.23 ± 3.85 (different age groups combined) BMI > 95th percentile for age and sex or > 2 BMI z score (country specific - Turkey)  Con: 20.45 ± 1.76 cd | 10-16  OB: nr Con: nr | OB: 40.5 Con: 51.9 | E/A | Turkey | cross-sectional case-control, single centre | No/Yes | No | fair |
| **Labombarda, 2013 ^96^** | Total: 64  OB: 32 Con: 32 | OB: 30.2 (20.8 - 42.6) [median (IQR)] BMI > 97th percentile for age and sex  Con: 18.06 ± 2.41 cd | 5-17  OB: 12.8 ± 2.1 Con: 12.8 ± 2.1 | OB: 46.9 Con: 46.9 | E wave, A wave, E/A, DT, e', E/e' | France | cross-sectional case-control, single centre | Yes/Yes | Yes  Unclear  [fasting glycaemia (mmol/L) × insulinaemia (mmol/L)]/22.5) | fair |
| **Levent, 2005 ^66^** | Total: 75  OB: 25 Con: 25 Other: 25 | OB: 26.9 ± 2.7 BMI > 95th percentile for age and sex (cd)  Con: 17.4 ± 3.2 cd  Other: 31.9 ± 4.4 OB hypertensive | nr  OB: 11.9 ± 1.5 Con: 12.1 ± 1.8 Other: 13.8 ± 2.4 | OB: 52 Con: 52 Other: 56 | E wave, A wave, E/A, DT, IVRT | Turkey | cross-sectional case-control, single centre | Yes/No | No | poor |
| **Lorch, 2007 ^51^** | Total: 168  OB: 33 Con: 115 OW: 20 | OB: 32.4 ± 8.2 BMI ≥ 95th percentile for age and sex (CDC)  Con: 19.7 ± 2.4 BMI 5th - 84th percentile for age and sex  OW: 24.3 ± 2.4 BMI 85th - 94th percentile for age and sex | 10-18  OB: 13.3 ± 2.2 Con: 13.9 ± 2.3 OW: 13.8 ± 2.4 | OB: 75.8 Con: 49.6 OW: 45.0 | E/A, IVRT, a', e'/a', diastolic strain | USA | cross-sectional case-control, single centre | Yes/Yes | No | fair |
| **Mangner, 2014 ^97^** | Total: 101  OW/OB: 61 Con: 40 | OW/OB: 30.8 ± 5.3 OB > 1.88 SD score (97th percentile), OW > 1.28 SD score (90th percentile) (country specific - German)  Con: 19 ± 2.6 cd | 8-21  OW/OB: 13.5 ± 2.7 Con: 14.1 ± 2.8 | OW/OB: 45.9 Con: 50.0 | E/A, DT, IVRT, e', a', E/e' | Germany | cross-sectional case-control, single centre | Yes/Yes | Yes  Overnight fast  Not reported | fair |
| **Marcovecchio, 2016 ^63^** | Total: 45  OB: 30 Con: 15 | OB: 28.7 ± 5.1 BMI > 95th percentile for age and sex (country specific - Italy)  Con: 19.4 ± 2.4 BMI 5th-85th percentile for age and sex | 6-17  OB: 11.5 ± 2.4 Con: 12.8 ± 3.1 | OB: 40.0 Con: 66.7 | E wave, A wave, E/A, DT, IVRT, e', a', E/e' | Italy | cross-sectional case-control, single centre | Yes/Yes | Yes  Unclear  [fasting insulin (mU/L)×fasting glucose (mmol/L)]/22.5 | good |
| **Mehta, 2004 ^98^** | Total: 116  OW/OB: 25  Con: 91 | OW/OB: 30.4 ± 6.5  BMI ≥ 25 kg/m2  Con: 20.2 ± 2.5  BMI < 25 kg/m2 | 10-18  OW/OB: 14.4 ± 2.1  Con: 13.8 ± 1.9 | OW/OB: 80.0  Con: 54.0 | E, A, E/A, e’, a’, E/e’, e’/a’ | USA | cross-sectional case-control, single centre | Yes/No | No | poor |
| **Mehta, 2009 ^99^** | Total: 49  OB: 17 Con: 32 | OB: 28.9 ± 5.4 WC > 90th percentile for age, sex and race (country specific - USA)  Con: 19.8 ± 4.0 WC ≤ 90th percentile for age, sex and race | 3-19  OB: 13.3 ± 3.2 Con: 13.3 ± 3.9 | OB: 76.5 Con: 59.4 | E wave, A wave, E/A, DT, e', a', E/e', e'/a' | USA | cross-sectional case-control, single centre | Yes/No | No | fair |
| **Metwalley, 2018 ^100^** | Total: 120  OB: 60  Con: 60 | OB: 3.6 ± 0.8 [BMI SDS – Egypt]  BMI > 95th percentile for age and sex (IOTF)  Con: 0.3 ± 1.1  Non-obese | nr  OB: 9.8 ± 2.2  Con: 10.6 ± 1.7 | OB: 56.7  Con:  60.0 | E/A, DT, IVRT | Egypt | cross-sectional case-control, single centre | No/No | Yes  Overnight fast (>12hrs)  [fasting insulin (µU/mL) x fasting glucose (mmol/L)]/22.5 | poor |
| **Naylor, 2008 ^101^** | Total: 23  OB: 13 Con: 10 | OB: 32.5 ± 1.9 BMI > 95th percentile for age and sex (IOTF)  Con: 30.2 ± 2.6 BMI > 95th percentile for age and sex (not undertaking intervention) | nr  OB: 12.2 ± 0.4 Con: 13.6 ± 0.7 | OB: 53.8 Con: 40.0 | E/A, DT, IVRT, e', a', E/e' | Australia | pre-post | Yes/Yes | No | fair |
| **Obert, 2012 ^50^** | Total: 61  OB: 37 Con: 24 | OB: 36.0 ± 5.1 BMI > 97th percentile for age and sex (cd)  Con: 20.8 ± 2.7 cd | nr  OB: 14.0 ± 1.5 Con: 13.6 ± 0.7 | OB: 29.7 Con: 37.5 | E wave, A wave, E/A, DT, IVRT, e', a', E/e', diastolic strain | France | cross-sectional case-control, single centre | Yes/Yes | Yes  Unclear  Not reported | fair |
| **Obert, 2013 ^102^** | Total: 48  OB: 28 Con: 20 | OB: 36.0 ± 4.6 BMI > 97th percentile for age and sex. BMI z score >3 indicated severe obesity (cd)  Con: 21.1 ± 2.6 cd | nr  OB: 14.2 ± 1.5 Con: 14.9 ± 1.6 | OB: 32.1 Con: 40.0 | E wave, A wave, E/A, IVRT, e', E/e', e'/a', diastolic strain | France | pre-post | Yes/Yes | Yes  Unclear  Not reported | good |
| **Ozcetin, 2012 ^45^** | Total: 78  OB: 42 Con: 36 | OB: 27.83 ± 2.53 BMI > 95th percentile for age and sex (cd)  Con: 19.03 ± 2.54 cd | 8-16  OB: 10.12 ± 2.12 Con: 9.78 ± 1.78 | OB: 42.9 Con: 38.9 | E wave, A wave, E/A, S wave, D wave, S/D, e', a', E/e', e'/a' | Turkey | cross-sectional case-control, single centre | Yes/Yes | No | fair |
| **Ozdemir, 2010 ^103^** | Total: 168  OB: 106 Con: 62 | OB: 28.1 ± 3.6 BMI z score > 2 (WHO)  Con: 18.1 ± 1.9 BMI z score ≤ 1 | nr  OB: 11.44 ± 2.30 Con: 11.75 ± 2.20 | OB: 55.7 Con: 58.1 | E/A, IVRT | Turkey | cross-sectional case-control, single centre | Yes/Yes | Yes  Fast >12hrs  Not reported | fair |
| **Pacifico, 2014 ^71^** | Total: 126  OB: 54 Con: 18 Other: 54 | OB: 2.0 (1.95-2.15) [BMI SDS, mean (95% CI)] BMI > 95th percentile for age and sex (IOTF)  Con: 0.47 (-0.13-1.0) cd  Other: 2.1 (2.0-2.21) OB with NAFLD | nr  OB: 12.6 (11.3-13.8) [mean (95% CI) Con: 12.5 (11.3-13.8) Other: 12.6 (11.3-13.8) | OB: 44.4 Con: 55.6 Other: 55.6 | E wave, A wave, E/A, DT, IVRT, e', a', E/e', e'/a' | Italy | cross-sectional case-control, single centre | No/Yes | Yes  Overnight fast  [fasting insulin (µU/mL) x fasting glucose (mmol/L)]/22.5 | fair |
| **Porcar-Almela, 2015 ^46^** | Total: 130  OB: 49 Con: 42 Other: 39 | OB: 3.06 ± 0.58 (BMI SDS) BMI z score > 2 (WHO)  Con: -0.04 ± 0.05 cd  Other: 5.49 ± 1.16 BMI z score > 4 | 7-16  OB: 11.45 ± 2.4 Con: 11.1 ± 2.7 Other: 10.6 ± 3.2 | OB: 59.2 Con: 50.0 Other: 48.7 | E wave, A wave, E/A, S wave, D wave, DT, IVRT, e', E/e' | Spain | cross-sectional case-control, single centre | No/Yes | Yes  Unclear  [fasting insulin (IU/L) x fasting glucose (mmol/L)]/22.5 | fair |
| **Saltijeral, 2011 ^104^** | Total: 72  OB: 30 Con: 42 | OB: 30.93 ± 6.67 BMI z score > 2 (country specific - Spain)  Con: 19.37 ± 3.02 cd | nr  OB: 13.25 ± 2.68 Con: 13.90 ± 2.56 | OB: cd Con: cd | E wave, A wave, E/A, e', a', E/e', e'/a' | Spain | cross-sectional case-control, single centre | No/Yes | No | fair |
| **Sanchez, 2015 ^49^** | Total: 58  OB: 34 Con: 14 Other: 10 | OB: 32 (30-38) [median (IQR)] BMI of ≥ 95th percentile for age and sex (CDC)  Con: 20 (17-23) BMI 5th-85th percentile for age and sex  Other: 41 (30-53) OB with abnormal LVMI and RWT | 12-18  OB: 14 (13-16) [median (IQR)] Con: 15 (13-17) Other: 14(12-15) | OB: 61.8 Con: 57.1 Other: 70.0 | Diastolic strain | USA | cross-sectional case-control, single centre | Yes/Yes | Yes  Overnight fast (>12hrs)  22.4/[glucose (mg/dL) x insulin (µU/mL)] | good |
| **Saritas, 2010 ^105^** | Total: 70  OB: 50 Con: 20 | OB: 26.0 ± 4.5 BMI > 97th percentile for age and sex (IOTF)  Con: 17 ± 2.6 cd | 66-166 months  OB: 125.58 ± 28.66 (months) Con: 121.30 ± 40.33 | OB: nr Con: nr | IVRT, e', a' | Turkey | cross-sectional case-control, single centre | Yes/No | No | poor |
| **Schuster, 2009 ^106^** | Total: 35  OB: 10 Con: 17 Other: 8 | OB: 23.3 ± 1.8 BMI > 97th percentile for age and sex, 1st Degree (country specific - France)  Con: 17.6 ± 0.6 cd  Other: 29.0 ± 2.0 BMI > 97th percentile for age and sex, 2nd Degree | 10-12  OB: 11.7 ± 0.6 Con: 11.6 ± 1.1 Other: 11.4 ± 1.0 | OB: 100 Con: 100 Other: 100 | E wave, A wave, E/A, DT, IVRT, e', a', E/e' | France | cross-sectional case-control, single centre | Yes/Yes | No | fair |
| **Schusterova, 2013 ^107^** | Total: 44  OW/OB: 21  Con: 23 | OW/OB: nr  BMI ≥ 85th percentile for age and sex  Con: nr  BMI < 85th percentile for age and sex | nr  OB: 13.5 ± 1.2  Con: 13.5 ± 1.1 | OB: nr  Con: nr | E wave, A wave, E/A, DT, IVRT | Slovakia | cross-sectional case-control, single centre | No/Yes | No | poor |
| **Sert, 2013 ^72^** | Total: 248  OB: 83 Con: 68 Other: 97 | OB: 29.7 ± 2.8 BMI ≥ 95th percentile for age and sex (country specific - Turkey)  Con: 19.2 ± 2.3 cd  Other: 30.2 ± 2.6 OB with NAFLD | 12-17  OB: 13.3 ± 1.3 Con: 13.5 ± 1.3 Other: 13.2 ± 1.4 | OB: 51.8 Con: 51.5 Other: 52.6 | E wave, A wave, E/A, e', a', e'/a' | Turkey | cross-sectional case-control, single centre | Yes/Yes | Yes  Overnight fast (≥12hrs)  fasting insulin concentration [mU/ml] x fasting glucose concentration [mmol/L]/22.5 | fair |
| **Shah, 2011 ^61^** | Total: 612  OB: 223 Con: 232 Other: 157 | OB: 37.6 ± 6.9 BMI ≥ 95th percentile for age and sex (CDC)  Con: 21.3 ± 2.4 BMI < 85th percentile for age and sex  Other: 39.1 ± 7.1 OB with Type 2 Diabetes | 10-24  OB: 18.1 ± 3.2 Con: 17.8 ± 3.5 Other: 17.9 ± 3.2 | OB: 29.1 Con: 38.4 Other: 34.4 | E wave, A wave, E/A, E/e', e'/a' | USA | cross-sectional case-control, single centre | Yes/Yes | No | good |
| **Shah, 2015 ^108^** | Total: 447  OB: 182  Severely OB: 265 | OB: 32.5 ± 2.9  BMI ≥ 100-119% of the 95th percentile for age and sex (CDC)  Severely OB: 42.7 ± 6.9  BMI ≥ 120% of the 95th percentile for age and sex | 10.2-23.9 | OB: 35.0  Severely OB: 31.0 | E/A, E/e’, e’/a’ | USA | cross-sectional case-control, single centre | Yes/No | No | poor |
| **Sharpe, 2006 ^109^** | Total: 43  OB: 18 Con: 15 | OB: 33.3 ± 1.0 BMI equivalent to ≥ 30 at age 18 (IOTF)  Con: 20.5 ± 0.7 BMI equivalent to < 25 at age 18 | nr  OB: 12.4 ± 0.4 Con: 13.3 ± 0.5 | OB: 50.0 Con: 53.3 | E wave, A wave, E/A, DT, e', a', E/e', e'/a' | Australia | cross-sectional case-control, single centre | Yes/No | No | poor |
| **Singh, 2013 ^48^** | Total: 44  OB: 15 Con: 14 Other: 15 | OB: 34.5 ± 2.9 BMI of ≥ 95th percentile for age and sex (cd)  Con: 19.9 ± 1.6 cd  Other: 37.4 ± 5.9 OB with NAFLD | nr  OB: 15 (14-17) [median (IQR)] Con: 15 (14-17) Other: 15 (13-16) | OB: 40.0 Con: 57.1 Other: 60.0 | Diastolic strain | USA | cross-sectional case-control, single centre | Yes/Yes | Yes  Unclear  Not reported | fair |
| **Van Putte-Katier, 2008 ^47^** | Total: 94  OB: 49 Con: 45 | OB: 28.3 ± 7.1 cd  Con: 17.2 ± 2.9 cd | nr  OB: 11.2 ± 2.8 Con: 11.5 ± 3.2 | OB: 44.9 Con: 48.9 | A wave, E/A, S wave, D wave, IVRT, e', E/e' | Belgium | cross-sectional case-control, single centre | Yes/Yes | No | fair |
| **Whalley, 2009 ^110^** | Total: 39  OW/OB: 11 Con: 9 Type 1 Diab: 11 Type 2 Diab: 8 | OW/OB: 30.9 ± 5.3 BMI ≥ 85th percentile for age and sex (IOTF)  Con: 20.8 ± 2 cd  Type 1 Diab: 24.5 ± 3.6 Type 2 Diab: 38.3 ± 7.4 | 12-18  OW/OB: 15.30 ± 1.51 Con: 14.90 ± 1.182 Type 1 Diab: 15.5 ± 1.1 Type 2 Diab: 14.9 ± 1.1 | OW/OB: 0 Con: 0 Diabetes: 0 | E wave, A wave, E/A, IVRT, e', E/e' | New Zealand | cross-sectional case-control, single centre | Yes/Yes | No | fair |
| **Xie, 2015 ^60^** | Total: 90  OB: 52 Con: 38 | OB: 31.5 ± 3.7 BMI of ≥ 95th percentile for age and sex (cd)  Con: 19.6 ± 2.1 cd | 14-20  OB: 17.8 ± 1.7 Con: 18.1 ± 2.1 | OB: 48.1 Con: 44.7 | E wave, A wave, E/A, DT, e', a', E/e', e'/a', diastolic strain | China | cross-sectional case-control, single centre | Yes/No | Yes  Overnight fast  [fasting glucose (mmol/l) x fasting insulin (mU/l)]/22.5 | fair |
| **Yang, 2019 ^111^** | Total: 181  OB: 40  Con: 102  Other: 39 | OB: nr  BMI ≥ 95th percentile and < 95th percentile x 120% for age and sex  Con: nr  BMI < 95^th^ percentile for age and sex  Other: ≥ 95th percentile x 120% (severe obesity) | 10-20  OB: 13.5 ± 2.7  Con: 13.8 ± 2.5  Other: 13.6 ± 2.5 | OB: 67.5  Con: 64.7  Other: 69.2 | E/A, DT, IVRT, e’, E/e’, e’/a’ | Taiwan | cross-sectional case-control, single centre | No/No | Yes  Unclear  [1.5 + fasting glucose × fasting C-peptide]/2800 | fair |
| **Yildirim, 2018 ^112^** | Total: 95  OB: 33 Con: 33 Other: 29 | OB: nr BMI ≥ 95th percentile for age and sex (cd)  Con: nr BMI 3rd - 85th percentiles for age and sex  Other: nr OB with anaemia | 12-17  OB: 14.18 ± 1.28 Con: 14.40 ± 1.42 Other: 13.89 ± 1.39 | OB: 54.5 Con: 54.5 Other: 44.8 | IVRT, e', a', E/e', e'/a' | Turkey | cross-sectional case-control, single centre | Yes/No | No | poor |
| **Yu, 2006 ^113^** | Total: 40  OW/OB: 22 Con: 18 | OW/OB: 28.4 ± 3.6 BMI ≥ 85th percentile for age and sex (country specific - South Korea)  Con: 17.9 ± 2.5 BMI < 85th percentile for age and sex | >10  OW/OB: 13.4 ± 1.22 Con: 13.4 ± 2.19 | OW/OB: 72.7 Con: 61.1 | E wave, A wave, E/A, S wave, D wave, S/D, e', a', e'/a' | South Korea | cross-sectional case-control, single centre | Yes/Yes | No | fair |
| **Zeybeck, 2010 ^114^** | Total: 58  OB: 34 Con: 24 | OB: 32.55 ± 2.96 BMI ≥ 95th percentile for age and sex (country specific - Turkey)  Con: 17.52 ± 2.58 BMI 5th-85th percentile for age and sex | nr  OB: 11.75 ± 2.23 Con: 11.25 ± 1.75 | OB: 50 Con: 50 | E wave, A wave, E/A, IVRT, e', a', E/e', e'/a' | Turkey | pre-post | Yes/No | Yes  Unclear  [fasting insulin (µU/ml) x fasting glucose (mmol/L)]/22.5 | fair |
| **Zhang, 2018 ^115^** | Total:  OB:  Con: | OB: 25.67 ± 3.78  BMI > 95th percentile for age and sex (country specific – China)  Con: 17.33 ± 2.58  BMI < 85th percentile for age and sex | 4-18  OB: 10.76 ± 2.72  Con: 11.49 ± 3.49 | OB: 57.5  Con: 49.3 | E wave, A wave, E/A, e’, a’, E/e’, e’/a’ | China | cross-sectional case-control, single centre | Yes/No | No | poor |

A wave indicates, peak late Doppler mitral inflow wave; a’, peak late diastolic tissue velocity; BMI, body mass index; cd, cannot determine; CDC, Centre for Disease Control and Prevention; CI, confidence interval; Con, control; D wave, peak pulmonary vein diastolic velocity; Diab, diabetes; DT, E wave deceleration time; E wave, peak early Doppler mitral inflow velocity; e’, peak early diastolic tissue velocity; E/A, E wave/A wave ratio; E/e’, E wave/e’ ratio; e’/a’, e’/a’ ratio; HOMA-IR, homeostatic model assessment for insulin resistance; IOTF, International Obesity Taskforce; IVRT, isovolumic relaxation time; IQR, interquartile range; LVMI, left ventricular mass index; MetS, metabolic syndrome; NAFLD, Non-alcoholic fatty liver disease; nr, not-reported; OB, obese; OW, overweight; RWT, relative wall thickness; S wave, peak pulmonary vein systolic velocity; SDS, standard deviation score; WHO, World Health Organization.

**Table S2:** Study Specific Positive (↑), Negative (↓), or Non-Significant (↔) Associations of Measures Left Ventricular Diastolic Function with Measures of Adiposity

| Study (first author, year) | E wave | A wave | E/A | DT | IVRT | e’ | a’ | E/e’ | e’/a’ | Diastolic Strain or SR | Pulmonary vein flow |
| --- | --- | --- | --- | --- | --- | --- | --- | --- | --- | --- | --- |
| Corica, 2020 ^55^ |  |  | ↓^a^ |  |  | ↓^b^ |  |  |  |  |  |
| Cozzolino, 2015 ^56^ |  |  |  |  |  |  |  |  | ↔^a^ |  |  |
| Dias, 2017 ^53^ |  |  |  |  |  |  |  |  |  | ↓ SR_E_^c^  ↔ SR_A_^c^ |  |
| El Saiedi, 2018 ^83^ |  |  |  |  |  |  |  | ↑^d^ |  |  |  |
| Hui, 2019 ^86^ |  |  |  |  |  |  |  | ↑^d^ |  |  |  |
| Kibar, 2015 ^52^ |  |  |  |  |  |  |  |  |  | ↓ SR_E_/SR_A_^d^ |  |
| Korkmaz, 2016 ^95^ | ↑^a,d^ |  | ↓^d^ |  |  |  |  |  |  |  |  |
| Lorch, 2007 ^51^ |  |  |  |  |  |  |  |  |  | ↓ long and rad SR_A_^d^ |  |
| Mangner, 2014 ^97^ |  |  |  |  |  | ↓^d^ |  |  |  |  |  |
| Marcovecchio, 2016 ^63^ | ↔^a,b^ |  |  |  |  |  |  |  |  |  |  |
| Mehta, 2009 ^99^ |  |  |  |  |  | ↓^b,d,e^ |  | ↑^b,d^ | ↓^b,d,e^ |  |  |
| Obert, 2012 ^50^ |  |  |  |  |  | ↓^a^ |  | ↑^a^ |  |  |  |
| Pacifico, 2014 ^71^ |  |  |  |  |  |  |  | ↔^a,c^ |  |  |  |
| Porcar-Almela, 2015 ^46^ |  |  |  |  |  |  |  | ↑^a,b,c^ |  |  |  |
| Saltijeral, 2011 ^104^ | ↓^d^ | ↔^d^ | ↔^d^ |  |  | ↔^d^ | ↔^d^ | ↔^d^ | ↓^d^ |  |  |
| Sanchez, 2015 ^49^ |  |  |  |  |  |  |  |  |  | ↔ SR_E_^a^ |  |
| Shah, 2011 ^61^ |  |  | ↓^b^ |  |  |  |  | ↑^a,b,c^ | ↓^a,b,c^ |  |  |
| Singh, 2013 ^48^ |  |  |  |  |  |  |  |  |  | ↔ SR_E_^c,d^ |  |
| Van Putte-Katier, 2008 ^47^ |  |  |  |  |  | ↓^a^ |  | ↑^a^ |  |  | ↑ S wave^a^ |

Arrows indicate studies that report either positive associations (↑), negative associations (↓), or no significant associations (↔) between a measure of adiposity (e.g. body mass index) and a measure of left ventricle diastolic dysfunction. A wave, peak late Doppler mitral inflow velocity; a’, peak late diastolic tissue velocity; DT, E wave deceleration time; E wave, peak early Doppler mitral inflow velocity; e’, peak early diastolic tissue velocity; E/A, E wave/A wave ratio; E/e’, E wave/e’ ratio; e’/a’, e’/a’ ratio; IVRT, isovolumic relaxation time; long, longitudinal; rad, radial; S wave, peak pulmonary vein systolic velocity; SR, diastolic strain rate; SR_A_, SR in late diastole; SR_A_, SR in early diastole.

^a^ Body mass index (BMI) standard deviation score (z score); ^b^ Waist circumference; ^c^ Body fat percentage/adipose tissue volume; ^d^ BMI; ^e^ Body surface area.

**Table S3:** Association of Left Ventricular Diastolic Function Measures with BMI, Age and Sex

| **Measure** | **N** | **Ns** | **r^2^** | **b** | **r** | **SE** | **z** | **LCI** | **UCI** | **p** |
| --- | --- | --- | --- | --- | --- | --- | --- | --- | --- | --- |
| **E wave (m/s)** | 66 | 33 | 0.143 |  |  |  |  |  |  |  |
| BMI (kg/m2) |  |  |  | 0.177 | 0.178 | 0.072 | 2.450 | 0.035 | 0.319 | 0.014 |
| Age (years) |  |  |  | -1.311 | -0.177 | 0.563 | -2.329 | -2.414 | -0.208 | 0.020 |
| Sex (male %) |  |  |  | 0.007 | 0.001 | 0.058 | 0.120 | -0.107 | 0.121 | 0.905 |
| **A wave (m/s)** | 68 | 34 | 0.272 |  |  |  |  |  |  |  |
| BMI (kg/m2) |  |  |  | 0.264 | 0.216 | 0.050 | 5.294 | 0.166 | 0.361 | 0.000 |
| Age (years) |  |  |  | -2.031 | -0.224 | 0.544 | -3.736 | -3.097 | -0.966 | 0.000 |
| Sex (male %) |  |  |  | 0.056 | 0.006 | 0.056 | 0.990 | -0.055 | 0.167 | 0.322 |
| **E/A** | 85 | 42 | 0.551 |  |  |  |  |  |  |  |
| BMI (kg/m2) |  |  |  | -0.006 | -0.147 | 0.001 | -4.561 | -0.008 | -0.003 | 0.000 |
| Age (years) |  |  |  | 0.059 | 0.209 | 0.010 | 5.750 | 0.039 | 0.079 | 0.000 |
| Sex (male %) |  |  |  | -0.001 | -0.003 | 0.002 | -0.448 | -0.005 | 0.003 | 0.654 |
| **DT (ms)** **^a^** | 31 | 14 | 0.263 |  |  |  |  |  |  |  |
| BMI (kg/m2) |  |  |  | -0.022 | -0.005 | 0.447 | -0.049 | -0.899 | 0.855 | 0.961 |
| Age (years) |  |  |  | -5.887 | -0.169 | 6.151 | -0.957 | -17.942 | 6.168 | 0.338 |
| Sex (male %) |  |  |  | -1.585 | -0.046 | 0.916 | -1.731 | -3.381 | 0.210 | 0.083 |
| **IVRT (ms)** | 37 | 17 | 0.152 |  |  |  |  |  |  |  |
| BMI (kg/m2) |  |  |  | 0.286 | 0.222 | 0.097 | 2.951 | 0.096 | 0.476 | 0.003 |
| Age (years) |  |  |  | -1.749 | -0.182 | 1.244 | -1.406 | -4.188 | 0.689 | 0.160 |
| Sex (male %) |  |  |  | -0.126 | -0.013 | 0.142 | -0.887 | -0.404 | 0.152 | 0.375 |
| **e' (cm/s)** **^a^** | 39 | 20 | 0.235 |  |  |  |  |  |  |  |
| BMI (kg/m2) |  |  |  | -0.091 | -0.294 | 0.020 | -4.583 | -0.130 | -0.052 | 0.000 |
| Age (years) |  |  |  | 0.012 | 0.005 | 0.297 | 0.040 | -0.571 | 0.595 | 0.968 |
| Sex (male %) |  |  |  | -0.023 | -0.010 | 0.019 | -1.221 | -0.060 | 0.014 | 0.222 |
| **e' sep (cm/s)** | 37 | 19 | 0.257 |  |  |  |  |  |  |  |
| BMI (kg/m2) |  |  |  | -0.075 | -0.413 | 0.016 | -4.726 | -0.106 | -0.044 | 0.000 |
| Age (years) |  |  |  | 0.386 | 0.287 | 0.164 | 2.356 | 0.065 | 0.708 | 0.018 |
| Sex (male %) |  |  |  | -0.019 | -0.014 | 0.017 | -1.083 | -0.052 | 0.015 | 0.279 |
| **e' lat (cm/s)** | 40 | 20 | 0.169 |  |  |  |  |  |  |  |
| BMI (kg/m2) |  |  |  | -0.116 | -0.247 | 0.021 | -5.554 | -0.157 | -0.075 | 0.000 |
| Age (years) |  |  |  | 0.290 | 0.083 | 0.327 | 0.887 | -0.351 | 0.930 | 0.375 |
| Sex (male %) |  |  |  | -0.011 | -0.003 | 0.038 | -0.283 | -0.086 | 0.064 | 0.777 |
| **a' (cm/s)** **^a^** | 27 | 14 | 0.295 |  |  |  |  |  |  |  |
| BMI (kg/m2) |  |  |  | 0.059 | 0.343 | 0.017 | 3.449 | 0.025 | 0.092 | 0.001 |
| Age (years) |  |  |  | -0.609 | -0.476 | 0.177 | -3.433 | -0.957 | -0.261 | 0.001 |
| Sex (male %) |  |  |  | -0.002 | -0.001 | 0.013 | -0.138 | -0.027 | 0.024 | 0.890 |
| **a' sep (cm/s)** | 31 | 16 | 0.421 |  |  |  |  |  |  |  |
| BMI (kg/m2) |  |  |  | 0.074 | 0.621 | 0.011 | 6.579 | 0.052 | 0.096 | 0.000 |
| Age (years) |  |  |  | -0.470 | -0.528 | 0.115 | -4.104 | -0.695 | -0.246 | 0.000 |
| Sex (male %) |  |  |  | 0.017 | 0.019 | 0.012 | 1.427 | -0.006 | 0.040 | 0.154 |
| **a' lat (cm/s)** | 28 | 14 | 0.179 |  |  |  |  |  |  |  |
| BMI (kg/m2) |  |  |  | 0.088 | 0.432 | 0.016 | 5.394 | 0.056 | 0.120 | 0.000 |
| Age (years) |  |  |  | -0.067 | -0.044 | 0.187 | -0.359 | -0.433 | 0.299 | 0.720 |
| Sex (male %) |  |  |  | -0.010 | -0.007 | 0.027 | -0.373 | -0.063 | 0.043 | 0.709 |
| **E/e' (cm/s)** | 29 | 16 | 0.646 |  |  |  |  |  |  |  |
| BMI (kg/m2) |  |  |  | 0.067 | 0.387 | 0.006 | 11.419 | 0.055 | 0.078 | 0.000 |
| Age (years) |  |  |  | 0.166 | 0.130 | 0.068 | 2.460 | 0.034 | 0.299 | 0.014 |
| Sex (male %) |  |  |  | 0.012 | 0.009 | 0.007 | 1.804 | -0.001 | 0.025 | 0.071 |
| **E/e' sep (cm/s)** **^a^** | 30 | 16 | 0.151 |  |  |  |  |  |  |  |
| BMI (kg/m2) |  |  |  | 0.081 | 0.431 | 0.011 | 7.220 | 0.059 | 0.104 | 0.000 |
| Age (years) |  |  |  | -0.064 | -0.045 | 0.131 | -0.484 | -0.321 | 0.194 | 0.628 |
| Sex (male %) |  |  |  | 0.022 | 0.015 | 0.014 | 1.588 | -0.005 | 0.048 | 0.112 |
| **E/e' lat (cm/s)** | 35 | 18 | 0.415 |  |  |  |  |  |  |  |
| BMI (kg/m2) |  |  |  | 0.046 | 0.237 | 0.006 | 7.174 | 0.034 | 0.059 | 0.000 |
| Age (years) |  |  |  | 0.159 | 0.110 | 0.087 | 1.827 | -0.012 | 0.331 | 0.068 |
| Sex (male %) |  |  |  | 0.003 | 0.002 | 0.008 | 0.391 | -0.013 | 0.019 | 0.696 |
| **e'/a’** | 21 | 11 | 0.570 |  |  |  |  |  |  |  |
| BMI (kg/m2) |  |  |  | -0.016 | -0.306 | 0.005 | -2.834 | -0.026 | -0.005 | 0.005 |
| Age (years) |  |  |  | 0.106 | 0.282 | 0.040 | 2.678 | 0.029 | 0.184 | 0.007 |
| Sex (male %) |  |  |  | 0.011 | 0.029 | 0.007 | 1.564 | -0.003 | 0.025 | 0.118 |
| **e'/a' sep** **^a^** | 25 | 13 | 0.544 |  |  |  |  |  |  |  |
| BMI (kg/m2) |  |  |  | -0.024 | -0.689 | 0.003 | -7.909 | -0.030 | -0.018 | 0.000 |
| Age (years) |  |  |  | 0.096 | 0.370 | 0.033 | 2.887 | 0.031 | 0.161 | 0.004 |
| Sex (male %) |  |  |  | -0.007 | -0.028 | 0.004 | -1.941 | -0.014 | 0.000 | 0.052 |
| **e'/a' lat** | 24 | 12 | 0.400 |  |  |  |  |  |  |  |
| BMI (kg/m2) |  |  |  | -0.037 | -0.593 | 0.008 | -4.525 | -0.052 | -0.021 | 0.000 |
| Age (years) |  |  |  | 0.102 | 0.223 | 0.059 | 1.727 | -0.014 | 0.218 | 0.084 |
| Sex (male %) |  |  |  | -0.006 | -0.012 | 0.014 | -0.400 | -0.032 | 0.021 | 0.689 |

A wave indicates peak late Doppler mitral inflow velocity; a’, peak late diastolic tissue velocity; Adj r2, adjusted r2; b, unstandardized regression coefficient; BMI, body mass index; CI, confidence interval (LCI, lower CI; UCI, upper CI); DT, E wave deceleration time; E wave, peak early Doppler mitral inflow velocity; e’, peak early diastolic tissue velocity; E/A, E wave/A wave ratio; E/e’, E wave/e’ ratio; e’/a’, e’/a’ ratio; IVRT, isovolumic relaxation time; N, number of observations; Ns, number of studies; r, standardized correlation coefficient; SE, standard error.

^a^, Non-normal distributions.

**Table S4:** Increased (↑), Decreased, (↓), or Unchanged (↔) Left Ventricular Diastolic Function in Obese/Overweight Children and Adolescents – Results of Studies Included in the Qualitative Analysis

| **Study (First Author, Year)** | **a’**  **(+)** | **E/e’**  **(+)** | **IVRT**  **(+)** | **A wave**  **(+)** | **E wave**  **(+)** | **DT**  **(=)** | **E/A**  **(-)** | **e’**  **(-)** | **e’/a’**  **(-)** |
| --- | --- | --- | --- | --- | --- | --- | --- | --- | --- |
| Ahmed, 2016 ^73^ | ↑^c^ |  |  |  |  |  |  | ↓^c^ | ↓^c^ |
| Akcaboy, 2016 ^64^ |  | ↔^c^ |  |  |  |  | ↔ |  |  |
| Alkholy, 2016 ^75^ |  |  |  | ↔ | ↔ |  | ↔ | ↔^c^ |  |
| Alp, 2014 ^76^ | ↑^b^ ↖^c^ | ↑^c^ | ↑^f^ | ↑ | ↑ |  | ↓ | ↔^b,c^ | ↓^b,c^ |
| Aslan, 2019 ^68^ |  |  |  | ↔ | ↔ |  | ↔ | ↔^b,c^ |  |
| Battal, 2011 ^77^ |  |  |  | ↔ | ↓ | ↑ | ↓ |  |  |
| Bjornstad, 2016 ^78^ | ↑^b,c^ | ↖^c^ ↔^b^ |  | ↑ | ↔ | ↓ | ↓ | ↖^c^ ↔^b^ |  |
| Chinali, 2008 ^69^ |  |  | ↔ |  |  | ↑ | ↓ |  |  |
| Corica, 2020 ^55^ |  |  |  |  |  |  | ↓ |  |  |
| Cozzolino, 2015 ^56^ | ↔^b^ | ↔^b^ |  | ↔ | ↓ |  | ↓ | ↔^b^ | ↓^b^ |
| Dahiya, 2015 ^57^ |  | ↑^b^ |  |  |  |  | ↔ | ↓^b^ |  |
| Dhuper, 2011 ^80^ |  | ↑^b,c^ |  |  |  |  | ↓ |  |  |
| Di Bonito, 2009 ^81^ |  |  | ↔ |  |  |  | ↔ |  | ↔^d^ |
| Dias, 2017 ^53^ | ↓^a^ | ↔^a^ |  | ↓ | ↔ |  | ↑ | ↓^a^ |  |
| Dusan, 2015 ^65^ |  |  | ↔ | ↑ | ↔ | ↔ | ↓ |  |  |
| El Saiedi, 2018 ^83^ | ↑^b,d^ | ↑^b^ |  | ↔ | ↓ |  | ↔ | ↓^b^ ↔^d^ |  |
| Franssen, 2019 ^58^ |  | ↑^a^ |  | ↑ | ↔ | ↔ | ↔ | ↓^a^ |  |
| Ghanem, 2010 ^44^ |  |  |  |  |  |  | ↔ |  |  |
| Hirschler, 2006 ^85^ |  |  |  |  |  |  | ↔ |  |  |
| Hui, 2019 ^86^ |  | ↑^c^ | ↔ | ↑ | ↑ |  | ↔ | ↓^c^ |  |
| Ingul, 2010 ^87^ |  | ↑^a^ | ↑ | ↔ | ↔ | ↑ | ↔ | ↓^a^ |  |
| Ingul, 2018 ^88^ | ↑^a^ | ↑^a^ |  |  |  |  | ↓ | ↓^a^ |  |
| Kibar, 2015 ^52^ | ↔^c^ ↘^b^ |  |  |  |  |  |  | ↔^b,c^ | ↖^b^ ↔^c^ |
| Korkmaz, 2016 ^95^ |  |  |  |  |  |  | ↓^e^ |  |  |
| Labombarda, 2013 ^96^ |  | ↖^c^ |  | ↔ | ↔ | ↔ | ↔ | ↘^c^ |  |
| Lorch, 2007 ^51^ |  |  | ↔ | ↔ | ↔ | ↔ | ↔ |  |  |
| Manger, 2014 ^97^ |  | ↑^b,c^ | ↖ |  |  | ↔ | ↔ | ↓^b,c^ |  |
| Marcovecchio, 2016 ^63^ | ↑^c^ ↔^b^ | ↔^b,c^ |  | ↔ | ↑ | ↑ | ↔ | ↔^b,c^ |  |
| Mehta, 2009 ^99^ | ↔^b,c^ | ↑^b,c^ |  | ↑ | ↔ |  | ↓ | ↓^b,c^ | ↓^b,c^ |
| Obert, 2012 ^50^ | ↔^d^ | ↑^d^ | ↔ | ↔ | ↔ | ↔ | ↔ | ↓^d^ |  |
| Obert, 2013 ^102^ |  |  |  |  |  |  |  |  | ↓^d^ |
| Ozcetin, 2012 ^45^ | ↑^c^ ↔^b^ | ↑^c^ ↖^b^ |  | ↔ | ↔ |  | ↔ | ↘^c^ ↔^b^ | ↓^c^ ↘^b^ |
| Ozdemir, 2010 ^103^ |  |  | ↔ |  |  |  | ↔ |  |  |
| Pacifico, 2014 ^71^ | ↔^a^ | ↔^a^ | ↑ | ↔ | ↔ | ↔ | ↔ |  | ↘^a^ |
| Porcar-Almela, 2015 ^46^ |  | ↑^a^ | ↔ | ↔ | ↖ | ↔ | ↔ |  |  |
| Saltijeral, 2011 ^104^ | ↔^c^ | ↖^c^ |  | ↔ | ↔ |  | ↔ | ↔^c^ | ↔^c^ |
| Schuster, 2009 ^106^ | ↔^a^ | ↔^c^ | ↑ | ↔ | ↔ | ↔ | ↔ |  |  |
| Schusterova, 2013 ^107^ |  |  | ↑ | ↑ | ↔ | ↑ | ↓ |  |  |
| Sert, 2013 ^72^ | ↑^b,c^ |  |  | ↔ | ↓ |  | ↓ | ↓^c^ ↔^b^ | ↓^b,c^ |
| Shah, 2011 ^61^ |  | ↑^a^ |  | ↑ | ↑ |  | ↔ |  | ↓^a^ |
| Van Putte-Katier, 2008 ^47^ |  | ↑^a^ | ↔ | ↔ |  |  | ↔ | ↓^b^ |  |
| Yildirim, 2018 ^112^ | ↔^d^ | ↖^d^ | ↔ |  |  |  |  | ↔^d^ | ↘^d^ |
| Zeybeck, 2010 ^114^ | ↔^b,c^ | ↑^c,f^ | ↔ | ↔ | ↔ |  | ↔ | ↓^c^ ↔^b^ | ↓^c^ ↔^b^ |

Arrows indicate increased (↑), a statistical trend (P < 0.1) towards increased values (↖), unchanged (↔), a statistical trend towards decreased values (↘), or decreased (↓) left ventricular (LV) diastolic function (DF) measures in overweight/obese children and adolescents compared to control subjects. LV DF measures are listed from the left in order of the strongest positive (+), no association (=), to the strongest negative (-) association with body mass index on the right as identified in our quantitative analysis.

^a^ Average of septal and lateral tissue Doppler imaging (TDI) measures; ^b^ Septal TDI measures; ^c^ Lateral TDI measures; ^d^ Cannot determine site of TDI; ^e^ In children aged 12-14 years only; ^f^ IVRT assessed via TDI.

**Table S5**: Association of Left Ventricular Diastolic Function Measures with HOMA-IR, Age and Sex

| **Measure** | **N** | **Ns** | **r^2^** | **b** | **r** | **SE** | **z** | **LCI** | **UCI** | **p** |
| --- | --- | --- | --- | --- | --- | --- | --- | --- | --- | --- |
| **E wave (m/s)** | 30 | 16 | 0.088 |  |  |  |  |  |  |  |
| *HOMA-IR* |  |  |  | 0.091 | 0.012 | 0.558 | 0.162 | -1.004 | 1.185 | 0.871 |
| *Age (years)* |  |  |  | -0.544 | -0.055 | 1.109 | -0.491 | -2.718 | 1.630 | 0.624 |
| *Sex (male %)* |  |  |  | -0.101 | -0.010 | 0.130 | -0.783 | -0.355 | 0.152 | 0.434 |
| **A wave (m/s)** | 30 | 16 | 0.259 |  |  |  |  |  |  |  |
| *HOMA-IR* |  |  |  | 1.169 | 0.157 | 0.288 | 4.064 | 0.605 | 1.733 | 0.000 |
| *Age (years)* |  |  |  | -3.684 | -0.375 | 0.932 | -3.953 | -5.510 | -1.857 | 0.000 |
| *Sex (male %)* |  |  |  | 0.261 | 0.027 | 0.076 | 3.447 | 0.112 | 0.409 | 0.001 |
| **E/A** | 47 | 24 | 0.289 |  |  |  |  |  |  |  |
| *HOMA-IR* |  |  |  | -0.035 | -0.159 | 0.012 | -2.864 | -0.059 | -0.011 | 0.004 |
| *Age (years)* |  |  |  | 0.103 | 0.354 | 0.027 | 3.826 | 0.050 | 0.156 | 0.000 |
| *Sex (male %)* |  |  |  | 0.001 | 0.004 | 0.004 | 0.326 | -0.006 | 0.008 | 0.744 |
| **DT (ms)** | 14 | 27 | 0.321 |  |  |  |  |  |  |  |
| *HOMA-IR* |  |  |  | -2.654 | -0.103 | 2.221 | -1.195 | -7.007 | 1.699 | 0.232 |
| *Age (years)* |  |  |  | -12.606 | -0.374 | 5.741 | -2.196 | -23.858 | -1.354 | 0.028 |
| *Sex (male %)* |  |  |  | -1.979 | -0.059 | 1.028 | -1.925 | -3.995 | 0.036 | 0.054 |
| **IVRT (ms)** | 27 | 13 | 0.443 |  |  |  |  |  |  |  |
| *HOMA-IR* |  |  |  | 3.560 | 0.463 | 1.148 | 3.102 | 1.310 | 5.810 | 0.002 |
| *Age (years)* |  |  |  | -4.219 | -0.418 | 1.257 | -3.356 | -6.683 | -1.755 | 0.001 |
| *Sex (male %)* |  |  |  | -0.456 | -0.045 | 0.256 | -1.780 | -0.959 | 0.046 | 0.075 |
| **e' (cm/s)** | 23 | 12 | 0.403 |  |  |  |  |  |  |  |
| *HOMA-IR* |  |  |  | -0.673 | -0.332 | 0.231 | -2.915 | -1.125 | -0.220 | 0.004 |
| *Age (years)* |  |  |  | -0.023 | -0.009 | 0.368 | -0.063 | -0.744 | 0.698 | 0.949 |
| *Sex (male %)* |  |  |  | -0.043 | -0.016 | 0.054 | -0.792 | -0.148 | 0.063 | 0.428 |
| **e' sep (cm/s)** | 15 | 8 | 0.300 |  |  |  |  |  |  |  |
| *HOMA-IR* |  |  |  | -0.132 | -0.098 | 0.136 | -0.966 | -0.399 | 0.136 | 0.334 |
| *Age (years)* |  |  |  | 0.608 | 0.346 | 0.448 | 1.358 | -0.270 | 1.486 | 0.174 |
| *Sex (male %)* |  |  |  | -0.007 | -0.004 | 0.043 | -0.166 | -0.091 | 0.077 | 0.868 |
| **e' lat (cm/s)** | 23 | 12 | 0.526 |  |  |  |  |  |  |  |
| *HOMA-IR* |  |  |  | -0.730 | -0.247 | 0.212 | -3.436 | -1.146 | -0.313 | 0.001 |
| *Age (years)* |  |  |  | -0.723 | -0.186 | 0.267 | -2.703 | -1.247 | -0.199 | 0.007 |
| *Sex (male %)* |  |  |  | -0.098 | -0.025 | 0.058 | -1.695 | -0.212 | 0.015 | 0.090 |
| **a' (cm/s)** | 14 | 8 | 0.352 |  |  |  |  |  |  |  |
| *HOMA-IR* |  |  |  | 0.295 | 0.247 | 0.055 | 5.341 | 0.187 | 0.404 | 0.000 |
| *Age (years)* |  |  |  | -1.092 | -0.695 | 0.381 | -2.866 | -1.839 | -0.345 | 0.004 |
| *Sex (male %)* |  |  |  | 0.037 | 0.024 | 0.012 | 2.989 | 0.013 | 0.062 | 0.003 |
| **a' sep (cm/s)** | 11 | 6 | 0.663 |  |  |  |  |  |  |  |
| *HOMA-IR* |  |  |  | 0.387 | 0.402 | 0.075 | 5.180 | 0.241 | 0.534 | 0.000 |
| *Age (years)* |  |  |  | -0.202 | -0.159 | 0.340 | -0.594 | -0.867 | 0.464 | 0.553 |
| *Sex (male %)* |  |  |  | -0.011 | -0.009 | 0.032 | -0.339 | -0.074 | 0.052 | 0.734 |
| **a' lat (cm/s)** | 13 | 7 | 0.801 |  |  |  |  |  |  |  |
| *HOMA-IR* |  |  |  | 0.190 | 0.137 | 0.119 | 1.589 | -0.044 | 0.423 | 0.112 |
| *Age (years)* |  |  |  | -0.708 | -0.390 | 0.121 | -5.843 | -0.945 | -0.471 | 0.000 |
| *Sex (male %)* |  |  |  | -0.081 | -0.045 | 0.064 | -1.268 | -0.206 | 0.044 | 0.205 |
| **E/e' (cm/s)** | 14 | 7 | 0.282 |  |  |  |  |  |  |  |
| *HOMA-IR* |  |  |  | 0.509 | 0.600 | 0.109 | 4.678 | 0.296 | 0.723 | 0.000 |
| *Age (years)* |  |  |  | -0.207 | -0.185 | 0.261 | -0.791 | -0.718 | 0.305 | 0.429 |
| *Sex (male %)* |  |  |  | 0.018 | 0.016 | 0.013 | 1.363 | -0.008 | 0.043 | 0.173 |
| **E/e' sep (cm/s)** **^a^** |  |  |  |  |  |  |  |  |  |  |
| *HOMA-IR* |  |  |  |  |  |  |  |  |  |  |
| *Age (years)* |  |  |  |  |  |  |  |  |  |  |
| *Sex (male %)* |  |  |  |  |  |  |  |  |  |  |
| **E/e' lat (cm/s)** | 16 | 9 | 0.283 |  |  |  |  |  |  |  |
| *HOMA-IR* |  |  |  | 0.161 | 0.156 | 0.074 | 2.195 | 0.017 | 0.306 | 0.028 |
| *Age (years)* |  |  |  | 0.184 | 0.135 | 0.229 | 0.805 | -0.264 | 0.632 | 0.421 |
| *Sex (male %)* |  |  |  | 0.008 | 0.006 | 0.023 | 0.343 | -0.038 | 0.054 | 0.731 |
| **e'/a’** | 9 | 5 | 0.529 |  |  |  |  |  |  |  |
| *HOMA-IR* |  |  |  | -0.056 | -0.174 | 0.024 | -2.314 | -0.103 | -0.009 | 0.021 |
| *Age (years)* |  |  |  | 0.093 | 0.221 | 0.116 | 0.805 | -0.134 | 0.321 | 0.421 |
| *Sex (male %)* |  |  |  | 0.016 | 0.038 | 0.031 | 0.526 | -0.044 | 0.076 | 0.599 |
| **e'/a' sep** | 9 | 5 | 0.000 |  |  |  |  |  |  |  |
| *HOMA-IR* |  |  |  | -0.098 | -0.412 | 0.033 | -2.953 | -0.163 | -0.033 | 0.003 |
| *Age (years)* |  |  |  | 0.002 | 0.006 | 0.077 | 0.026 | -0.148 | 0.152 | 0.980 |
| *Sex (male %)* |  |  |  | -0.020 | -0.065 | 0.018 | -1.146 | -0.055 | 0.014 | 0.252 |
| **e'/a' lat** | 14 | 7 | 0.147 |  |  |  |  |  |  |  |
| *HOMA-IR* |  |  |  | -0.094 | -0.291 | 0.019 | -4.839 | -0.132 | -0.056 | 0.000 |
| *Age (years)* |  |  |  | -0.073 | -0.173 | 0.129 | -0.568 | -0.325 | 0.179 | 0.570 |
| *Sex (male %)* |  |  |  | -0.113 | -0.267 | 0.027 | -4.197 | -0.166 | -0.060 | 0.000 |

A wave indicates peak late Doppler mitral inflow velocity; a’, peak late diastolic tissue velocity; Adj r2, adjusted r2; b, unstandardized regression coefficient; CI, confidence interval (LCI, lower CI; UCI, upper CI); DT, E wave deceleration time; E wave, peak early Doppler mitral inflow velocity; e’, peak early diastolic tissue velocity; E/A, E wave/A wave ratio; E/e’, E wave/e’ ratio; e’/a’, e’/a’ ratio; HOMA-IR, homeostatic model assessment of insulin resistance; IVRT, isovolumic relaxation time; N, number of observations; Ns, number of studies; r, standardized correlation coefficient; SE, standard error.

^a^, Insufficient number of studies to be included.

# Study Quality Assessment Tools

These tools and guidance are modified from the Study Quality Assessment Tools by the National Heart, Lung, and Blood Institute (NHLBI) to assess study quality and risk of bias.^116^ These tools score papers as *good*, *fair* or *poor* based on questions that focus on important key concepts for critical appraisal without providing a numeric score which have been described as misleading and unhelpful.

### Case Control and Cross-Sectional Studies

| **Reference** | 1. Was the research question or objective in this paper clearly stated and appropriate? | 2. Was the study population clearly specified and defined? | 3. Did the authors include a sample size justification? | 4. Were controls selected or recruited from the same or similar population that gave rise to the cases (including the same timeframe)? | 5. Were the definitions, inclusion and exclusion criteria, algorithms or processes used to identify or select cases and controls valid, reliable, and implemented consistently across all study participants? | 6. Were the cases clearly defined and differentiated from controls? | 7. If less than 100 percent of eligible cases and/or controls were selected for the study, were the cases and/or controls randomly selected from those eligible? | 8. Was there use of concurrent controls? | 9. Were the investigators able to confirm that the exposure/risk occurred prior to the development of the condition or event that defined a participant as a case? | 10. For exposures that can vary in amount or level (obesity), did the study examine different levels of the exposure as related to the outcome [e.g., categories of obesity, or correlation statistics (r, r2 and/or β]? | 11. Were the measures of diastolic function defined, valid, reliable, and implemented consistently (including the same time period) across all study participants? | 12. Were the assessors of diastolic function blinded to the case or control status of participants? | 13. Was the statistical analysis clear and appropriate? | 14. Were key potential confounding variables measured and adjusted statistically in the analyses? If matching was used, did the investigators account for matching during study analysis? | 15. Were the results internally consistent? | **Quality Rating (Good, Fair or Poor)** (see guidance) | Raters Initials | Comments |
| --- | --- | --- | --- | --- | --- | --- | --- | --- | --- | --- | --- | --- | --- | --- | --- | --- | --- | --- |
|  |  |  |  |  |  |  |  |  |  |  |  |  |  |  |  |  |  |  |
|  |  |  |  |  |  |  |  |  |  |  |  |  |  |  |  |  |  |  |

#### Guidance

1. Was the research question or objective in this paper clearly stated and appropriate?

Did the authors describe their goal in conducting this research? Is it easy to understand what they were looking to find? This issue is important for any scientific paper of any type. High quality scientific research explicitly defines a research question.

2. Was the study population clearly specified and defined?

Did the authors describe the group of individuals from which the cases and controls were selected or recruited, while using demographics, location, and time period? If the investigators conducted this study again, would they know exactly who to recruit, from where, and from what time period?

Investigators identify case-control study populations by location, time period, and inclusion criteria for cases (individuals with the disease, condition, or problem) and controls (individuals without the disease, condition, or problem). For example, the population for a study of lung cancer and chemical exposure would be all incident cases of lung cancer diagnosed in patients ages 35 to 79, from January 1, 2003 to December 31, 2008, living in Texas during that entire time period, as well as controls without lung cancer recruited from the same population during the same time period. The population is clearly described as: (1) who (men and women ages 35 to 79 with (cases) and without (controls) incident lung cancer); (2) where (living in Texas); and (3) when (between January 1, 2003 and December 31, 2008).

Other studies may use disease registries or data from cohort studies to identify cases. In these cases, the populations are individuals who live in the area covered by the disease registry or included in a cohort study (i.e., nested case-control or case-cohort). For example, a study of the relationship between vitamin D intake and myocardial infarction might use patients identified via the GRACE registry, a database of heart attack patients.

NHLBI staff encouraged reviewers to examine prior papers on methods (listed in the reference list) to make this assessment, if necessary

3. Did the authors include a sample size justification?

Did the authors discuss their reasons for selecting or recruiting the number of individuals included? Did they discuss the statistical power of the study and provide a sample size calculation to ensure that the study is adequately powered to detect an association (if one exists)? This question does not refer to a description of the manner in which different groups were included or excluded using the inclusion/exclusion criteria (e.g., "Final study size was 1,378 participants after exclusion of 461 patients with missing data" is not considered a sample size justification for the purposes of this question).

An article's methods section usually contains information on sample size and the size needed to detect differences in exposures and on statistical power.

4. Were controls selected or recruited from the same or similar population that gave rise to the cases (including the same timeframe)?

To determine whether cases and controls were recruited from the same population, one can ask hypothetically, "If a control was to develop the outcome of interest (the condition that was used to select cases), would that person have been eligible to become a case?" Case-control studies begin with the selection of the cases (those with the outcome of interest, e.g., lung cancer) and controls (those in whom the outcome is absent). Cases and controls are then evaluated and categorized by their exposure status. For the lung cancer example, cases and controls were recruited from hospitals in a given region. One may reasonably assume that controls in the catchment area for the hospitals, or those already in the hospitals for a different reason, would attend those hospitals if they became a case; therefore, the controls are drawn from the same population as the cases. If the controls were recruited or selected from a different region (e.g., a State other than Texas) or time period (e.g., 1991-2000), then the cases and controls were recruited from different populations, and the answer to this question would be "no."

5. Were the definitions, inclusion and exclusion criteria, algorithms or processes used to identify or select cases and controls valid, reliable, and implemented consistently across all study participants?

Were the inclusion and exclusion criteria developed prior to recruitment or selection of the study population? Were the same underlying criteria used for all of the groups involved? To answer this question, reviewers determined if the investigators developed I/E criteria prior to recruitment or selection of the study population and if they used the same underlying criteria for all groups. The investigators should have used the same selection criteria, except for study participants who had the disease or condition, which would be different for cases and controls by definition. Therefore, the investigators use the same age (or age range), gender, race, and other characteristics to select cases and controls. Information on this topic is usually found in a paper's section on the description of the study population.

Please note that self-reported height, weight, BMI etc. are not valid or reliable measures.

6. Were the cases clearly defined and differentiated from controls?

For this question, reviewers looked for descriptions of the validity of case and control definitions and processes or tools used to identify study participants as such. Was a specific description of "case" and "control" provided? Is there a discussion of the validity of the case and control definitions and the processes or tools used to identify study participants as such? They determined if the tools or methods were accurate, reliable, and objective. For example, cases might be identified as "adult patients admitted to a VA hospital from January 1, 2000 to December 31, 2009, with an ICD-9 discharge diagnosis code of acute myocardial infarction and at least one of the two confirmatory findings in their medical records: at least 2mm of ST elevation changes in two or more ECG leads and an elevated troponin level. Investigators might also use ICD-9 or CPT codes to identify patients. All cases should be identified using the same methods. Unless the distinction between cases and controls is accurate and reliable, investigators cannot use study results to draw valid conclusions.

7. If less than 100 percent of eligible cases and/or controls were selected for the study, were the cases and/or controls randomly selected from those eligible?

If a case-control study did not use 100 percent of eligible cases and/or controls (e.g., not all disease-free participants were included as controls), did the authors indicate that random sampling was used to select controls? When it is possible to identify the source population fairly explicitly (e.g., in a nested case-control study, or in a registry-based study), then random sampling of controls is preferred. When investigators used consecutive sampling (recruiting until you meet the desired sample size), which is frequently done for cases in prospective studies, then study participants are not considered randomly selected. In this case, the reviewers would answer "no" to Question 7. However, this would not be considered a fatal flaw.

If investigators included all eligible cases and controls as study participants, then reviewers marked "NA" in the tool. If 100 percent of cases were included (e.g., NA for cases) but only 50 percent of eligible controls, then the response would be "yes" if the controls were randomly selected, and "no" if they were not. If this cannot be determined, the appropriate response is "CD."

8. Was there use of concurrent controls?

A concurrent control is a control selected at the time another person became a case, usually on the same day. This means that one or more controls are recruited or selected from the population without the outcome of interest at the time a case is diagnosed. Investigators can use this method in both prospective case-control studies and retrospective case-control studies. For example, in a retrospective study of adenocarcinoma of the colon using data from hospital records, if hospital records indicate that Person A was diagnosed with adenocarcinoma of the colon on June 22, 2002, then investigators would select one or more controls from the population of patients without adenocarcinoma of the colon on that same day. This assumes they conducted the study retrospectively, using data from hospital records. The investigators could have also conducted this study using patient records from a cohort study, in which case it would be a nested case-control study.

Investigators can use concurrent controls in the presence or absence of matching and vice versa. A study that uses matching does not necessarily mean that concurrent controls were used.

9. Were the investigators able to confirm that the exposure/risk occurred prior to the development of the condition or event that defined a participant as a case?

For an association between an exposure and an outcome to be considered causal, the exposure must have occurred prior to the outcome. Therefore, were the investigators able to provide data showing that obesity occurred prior to the development of impaired left ventricular diastolic function (diastolic dysfunction)? Most studies will most likely be “no” for this question.

10. For exposures that can vary in amount or level, did the study examine different levels of the exposure as related to the outcome [e.g., categories of obesity, or correlation statistics (r, r2 and/or β]?

If the exposure can be defined as a range (examples: level of overweight/obesity, drug dosage, amount of physical activity, amount of sodium consumed), were multiple categories of that exposure assessed? (for example, for drugs: not on the medication, on a low dose, medium dose, high dose; for dietary sodium, higher than average U.S. consumption, lower than recommended consumption, between the two). Sometimes discrete categories of exposure are not used, but instead exposures are measured as continuous variables (for example, mg/day of dietary sodium or BP values or BMI).

In any case, studying different levels of exposure (where possible) enables investigators to assess trends or dose-response relationships between exposures and outcomes–e.g., the higher the exposure, the greater the rate of the health outcome. The presence of trends or dose-response relationships lends credibility to the hypothesis of causality between exposure and outcome.

For some exposures, however, this question may not be applicable (e.g., the exposure may be a dichotomous variable like living in a rural setting versus an urban setting, or vaccinated/not vaccinated with a one-time vaccine). If there are only two possible exposures (yes/no), then this question should be given an "NA," and it should not count negatively towards the quality rating.

11. Were the measures of diastolic function defined, valid, reliable, and implemented consistently (including the same time period) across all study participants?

Were the exposure measures defined in detail? Were the tools or methods used to measure exposure accurate and reliable–for example, have they been validated or are they objective? This is important, as it influences confidence in the reported exposures. Equally important is whether the exposures were assessed in the same manner within groups and between groups. This question pertains to bias resulting from exposure misclassification (i.e., exposure ascertainment).

For example, a retrospective self-report of dietary salt intake is not as valid and reliable as prospectively using a standardized dietary log plus testing participants' urine for sodium content because participants' retrospective recall of dietary salt intake may be inaccurate and result in misclassification of exposure status. Similarly, BP results from practices that use an established protocol for measuring BP would be considered more valid and reliable than results from practices that did not use standard protocols. A protocol may include using trained BP assessors, standardized equipment (e.g., the same BP device which has been tested and calibrated), and a standardized procedure (e.g., patient is seated for 5 minutes with feet flat on the floor, BP is taken twice in each arm, and all four measurements are averaged).

If the study has reported individuals with Diastolic Dysfunction or Metabolic Syndrome then have the authors provided a reliable reason as to why they have classified individuals into these groups? This needs to be considered carefully as there are no standardised definitions for these in children.

12. Were the assessors of diastolic function blinded to the case or control status of participants?

Blinding or masking means that outcome assessors did not know whether participants were exposed or unexposed. To answer this question, reviewers examined articles for evidence that the outcome assessor(s) was masked to the exposure status of the research participants. An outcome assessor, for example, may examine medical records to determine the outcomes that occurred in the exposed and comparison groups. Sometimes the person measuring the exposure is the same person conducting the outcome assessment. In this case, the outcome assessor would most likely not be blinded to exposure status. A reviewer would note such a finding in the comments section of the assessment tool.

One way to ensure good blinding of exposure assessment is to have a separate committee, whose members have no information about the study participants' status as cases or controls, review research participants' records. To help answer the question above, reviewers determined if it was likely that the outcome assessor knew whether the study participant was a case or control. If it was unlikely, then the reviewers marked "no" to Question 12. Outcome assessors who used medical records to assess exposure should not have been directly involved in the study participants' care, since they probably would have known about their patients' conditions. If the medical records contained information on the patient's condition that identified him/her as a case (which is likely), that information would have had to be removed before the exposure assessors reviewed the records.

If blinding was not possible, which sometimes happens, the reviewers marked "NA" in the assessment tool and explained the potential for bias.

13. Was the statistical analysis clear and appropriate?

While interpretation of statistics can be quite difficult, a basic understanding of statistics can help you to assess the quality of the paper. Often many different methods can be used correctly to test the same data, but as there is such a wide range available, knowing what tests are most appropriate in particular situations can be hard to decipher. There is an expectation that the researcher has this understanding or has at least sought statistical assistance to ensure that the correct methods are used. Therefore for question 10 the emphasis for the reader is that the statistical methods, software packages used and the statistical significance levels are clearly stated even if the paper is just presenting descriptive statistics. The statistical significance level is usually described as a p-value. In most cases the p-value, at which the null hypothesis is rejected, is set at 0.05. The higher the p-value is set the greater the possibility of introducing a type I error. Confidence intervals should also be declared with p-values or instead of p-values as an indication of the precision of the estimates. It is usual to present a confidence interval of 95% which means that the researchers were 95 per cent confident that the true population value of the outcome lies between these intervals. This can be used to compare groups where an overlap would suggest no difference and a gap between confidence intervals would suggest a difference.

To fully establish the difference between groups, means ± SDs, p values, confidence intervals and effect sizes [e.g. Cohen’s d, Correlation Coefficient or Pearson’s r correlation (r) or Coefficient of determination (r2)] should be reported.

If the study is establishing the link or association between “x” and “y” (e.g. the effect of obesity on left ventricular diastolic function measured using E/e’) then the authors have to report effect sizes [r, r2 and/or beta coefficient (β)] with p values.

14. Were key potential confounding variables measured and adjusted statistically in the analyses? If matching was used, did the investigators account for matching during study analysis?

Were key potential confounding variables measured and adjusted for, such as by statistical adjustment for baseline differences? Investigators often use logistic regression or other regression methods to account for the influence of variables not of interest.

This is a key issue in case-controlled studies; statistical analyses need to control for potential confounders, in contrast to RCTs in which the randomization process controls for potential confounders. In the analysis, investigators need to control for all key factors that may be associated with both the exposure of interest and the outcome and are not of interest to the research question.

A study of the relationship between smoking and CVD events illustrates this point. Such a study needs to control for age, gender, and body weight; all are associated with smoking and CVD events. Well-done case-control studies control for multiple potential confounders.

Matching is a technique used to improve study efficiency and control for known confounders. For example, in the study of smoking and CVD events, an investigator might identify cases that have had a heart attack or stroke and then select controls of similar age, gender, and body weight to the cases. For case-control studies, it is important that if matching was performed during the selection or recruitment process, the variables used as matching criteria (e.g., age, gender, race) should be controlled for in the analysis.

15. Were the results internally consistent?

Question 14 is an exploration of the basic data and asks that the reader spends some time exploring the numbers given in the results; in the text, figures and tables. Information about the level of missing data should also be declared in the results. It is important to check that the numbers add up in the tables and the text. If the study has recruited 100 participants, the tables and the text should include data about 100 participants. If not, the missing data should be clearly declared and the reason for its non-appearance explained.

**General Guidance for Determining the Overall Quality Rating of Case-Controlled Studies**

NHLBI designed the questions in the assessment tool to help reviewers focus on the key concepts for evaluating a study's internal validity, not to use as a list from which to add up items to judge a study's quality.

Internal validity for case-control studies is the extent to which the associations between disease and exposure reported in the study can truly be attributed to the exposure being evaluated rather than to flaws in the design or conduct of the study. In other words, what is ability of the study to draw associative conclusions about the effects of the exposures on outcomes? Any such flaws can increase the risk of bias.

In critical appraising a study, the following factors need to be considered: risk of potential for selection bias, information bias, measurement bias, or confounding (the mixture of exposures that one cannot tease out from each other). Examples of confounding include co-interventions, differences at baseline in patient characteristics, and other issues addressed in the questions above. High risk of bias translates to a poor quality rating; low risk of bias translates to a good quality rating. Again, the greater the risk of bias, the lower the quality rating of the study.

In addition, the more attention in the study design to issues that can help determine whether there is a causal relationship between the outcome and the exposure, the higher the quality of the study. These include exposures occurring prior to outcomes, evaluation of a dose-response gradient, accuracy of measurement of both exposure and outcome, sufficient timeframe to see an effect, and appropriate control for confounding–all concepts reflected in the tool.

If a study has a "fatal flaw," then risk of bias is significant; therefore, the study is deemed to be of poor quality. An example of a fatal flaw in case-control studies is a lack of a consistent standard process used to identify cases and controls.

Generally, when reviewers evaluated a study, they did not see a "fatal flaw," but instead found some risk of bias. By focusing on the concepts underlying the questions in the quality assessment tool, reviewers examined the potential for bias in the study. For any box checked "no," reviewers asked, "What is the potential risk of bias resulting from this flaw in study design or execution?" That is, did this factor lead to doubt about the results reported in the study or the ability of the study to accurately assess an association between exposure and outcome?

By examining questions in the assessment tool, reviewers were best able to assess the potential for bias in a study. Specific rules were not useful, as each study had specific nuances. In addition, being familiar with the key concepts helped reviewers assess the studies. Examples of studies rated good, fair, and poor were useful, yet each study had to be assessed on its own.

### Pre-Post Studies

| **Reference** | 1. Was the study question or objective clearly stated? | 2. Were eligibilitYes/selection criteria for the study population prespecified and clearly described? | 3. Were the participants in the study representative of those who would be eligible for the test/service/intervention in the general or clinical population of interest? | 4. Were all eligible participants that met the prespecified entry criteria enrolled? | 5. Was the sample size sufficiently large to provide confidence in the findings? | 6. Was the test/service/intervention clearly described and delivered consistently across the study population? | 7. Were the outcome measures prespecified, clearly defined, valid, reliable, and assessed consistently across all study participants? | 8. Were the people assessing the outcomes blinded to the participants' exposures/interventions? | 9. Was the loss to follow-up after baseline 20% or less? Were those lost to follow-up accounted for in the analysis? | 10. Did the statistical methods clear and appropriate and examine changes in outcome measures from before to after the intervention? Were statistical tests done that provided p values for the pre-to-post changes? | 11. Were outcome measures of interest taken multiple times before the intervention and multiple times after the intervention (i.e., did they use an interrupted time-series design)? | 12. If the intervention was conducted at a group level (e.g., a whole hospital, a community, etc.) did the statistical analysis take into account the use of individual-level data to determine effects at the group level? | **Quality Rating (Good, Fair or Poor) (see guidance)** | **Raters Initials** | **Comments** |
| --- | --- | --- | --- | --- | --- | --- | --- | --- | --- | --- | --- | --- | --- | --- | --- |
|  |  |  |  |  |  |  |  |  |  |  |  |  |  |  |  |
|  |  |  |  |  |  |  |  |  |  |  |  |  |  |  |  |

#### Guidance

1. Study question

Did the authors describe their goal in conducting this research? Is it easy to understand what they were looking to find? This issue is important for any scientific paper of any type. Higher quality scientific research explicitly defines a research question.

1. Eligibility criteria and study population

Did the authors describe the eligibility criteria applied to the individuals from whom the study participants were selected or recruited? In other words, if the investigators were to conduct this study again, would they know whom to recruit, from where, and from what time period?

Here is a sample description of a study population: men over age 40 with type 2 diabetes, who began seeking medical care at Phoenix Good Samaritan Hospital, between January 1, 2005 and December 31, 2007. The population is clearly described as: (1) who (men over age 40 with type 2 diabetes); (2) where (Phoenix Good Samaritan Hospital); and (3) when (between January 1, 2005 and December 31, 2007). Another sample description is women who were in the nursing profession, who were ages 34 to 59 in 1995, had no known CHD, stroke, cancer, hypercholesterolemia, or diabetes, and were recruited from the 11 most populous States, with contact information obtained from State nursing boards.

To assess this question, reviewers examined prior papers on study methods (listed in reference list) when necessary.

1. Study participants representative of clinical populations of interest

The participants in the study should be generally representative of the population in which the intervention will be broadly applied. Studies on small demographic subgroups may raise concerns about how the intervention will affect broader populations of interest. For example, interventions that focus on very young or very old individuals may affect middle-aged adults differently. Similarly, researchers may not be able to extrapolate study results from patients with severe chronic diseases to healthy populations.

1. All eligible participants enrolled

To further explore this question, reviewers may need to ask: Did the investigators develop the I/E criteria prior to recruiting or selecting study participants? Were the same underlying I/E criteria used for all research participants? Were all subjects who met the I/E criteria enrolled in the study?

1. Sample size

Did the authors present their reasons for selecting or recruiting the number of individuals included or analysed? Did they note or discuss the statistical power of the study? This question addresses whether there was a sufficient sample size to detect an association, if one did exist.

An article's methods section may provide information on the sample size needed to detect a hypothesized difference in outcomes and a discussion on statistical power (such as, the study had 85 percent power to detect a 20 percent increase in the rate of an outcome of interest, with a 2-sided alpha of 0.05). Sometimes estimates of variance and/or estimates of effect size are given, instead of sample size calculations. In any case, if the reviewers determined that the power was sufficient to detect the effects of interest, then they would answer "yes" to Question 5.

1. Intervention clearly described

Another pertinent question regarding interventions is: Was the intervention clearly defined in detail in the study? Did the authors indicate that the intervention was consistently applied to the subjects? Did the research participants have a high level of adherence to the requirements of the intervention? For example, if the investigators assigned a group to 10 mg/day of Drug A, did most participants in this group take the specific dosage of Drug A? Or did a large percentage of participants end up not taking the specific dose of Drug A indicated in the study protocol?

Reviewers ascertained that changes in study outcomes could be attributed to study interventions. If participants received interventions that were not part of the study protocol and could affect the outcomes being assessed, the results could be biased.

1. Outcome measures clearly described, valid, and reliable

Were the outcomes defined in detail? Were the tools or methods for measuring outcomes accurate and reliable–for example, have they been validated or are they objective? This question is important because the answer influences confidence in the validity of study results.

An example of an outcome measure that is objective, accurate, and reliable is death–the outcome measured with more accuracy than any other. But even with a measure as objective as death, differences can exist in the accuracy and reliability of how investigators assessed death. For example, did they base it on an autopsy report, death certificate, death registry, or report from a family member? Another example of a valid study is one whose objective is to determine if dietary fat intake affects blood cholesterol level (cholesterol level being the outcome) and in which the cholesterol level is measured from fasting blood samples that are all sent to the same laboratory. These examples would get a "yes."

An example of a "no" would be self-report by subjects that they had a heart attack, or self-report of how much they weight (if body weight is the outcome of interest)

1. Blinding of outcome assessors

Blinding or masking means that the outcome assessors did not know whether the participants received the intervention or were exposed to the factor under study. To answer the question above, the reviewers examined articles for evidence that the person(s) assessing the outcome(s) was masked to the participants' intervention or exposure status. An outcome assessor, for example, may examine medical records to determine the outcomes that occurred in the exposed and comparison groups. Sometimes the person applying the intervention or measuring the exposure is the same person conducting the outcome assessment. In this case, the outcome assessor would not likely be blinded to the intervention or exposure status. A reviewer would note such a finding in the comments section of the assessment tool.

In assessing this criterion, the reviewers determined whether it was likely that the person(s) conducting the outcome assessment knew the exposure status of the study participants. If not, then blinding was adequate. An example of adequate blinding of the outcome assessors is to create a separate committee whose members were not involved in the care of the patient and had no information about the study participants' exposure status. Using a study protocol, committee members would review copies of participants' medical records, which would be stripped of any potential exposure information or personally identifiable information, for prespecified outcomes.

1. Follow up rate

Higher overall follow-up rates are always desirable to lower follow-up rates, although higher rates are expected in shorter studies, and lower overall follow-up rates are often seen in longer studies. Usually an acceptable overall follow-up rate is considered 80 percent or more of participants whose interventions or exposures were measured at baseline. However, this is a general guideline.

In accounting for those lost to follow-up, in the analysis, investigators may have imputed values of the outcome for those lost to follow-up or used other methods. For example, they may carry forward the baseline value or the last observed value of the outcome measure and use these as imputed values for the final outcome measure for research participants lost to follow-up.

1. Statistical analysis

Were formal statistical tests used to assess the significance of the changes in the outcome measures between the before and after time periods? The reported study results should present values for statistical tests, such as p values, to document the statistical significance (or lack thereof) for the changes in the outcome measures found in the study.

1. Multiple outcome measures

Were the outcome measures for each person measured more than once during the course of the before and after study periods? Multiple measurements with the same result increase confidence that the outcomes were accurately measured.

1. Group-level interventions and individual-level outcome efforts

Group-level interventions are usually not relevant for clinical interventions such as bariatric surgery, in which the interventions are applied at the individual patient level. In those cases, the questions were coded as "NA" in the assessment tool.

**General Guidance for Determining the Overall Quality Rating of Pre-Post Studies**

The questions in the quality assessment tool were designed to help reviewers focus on the key concepts for evaluating the internal validity of a study. They are not intended to create a list from which to add up items to judge a study's quality.

Internal validity is the extent to which the outcome results reported in the study can truly be attributed to the intervention or exposure being evaluated, and not to biases, measurement errors, or other confounding factors that may result from flaws in the design or conduct of the study. In other words, what is the ability of the study to draw associative conclusions about the effects of the interventions or exposures on outcomes?

Critical appraisal of a study involves considering the risk of potential for selection bias, information bias, measurement bias, or confounding (the mixture of exposures that one cannot tease out from each other). Examples of confounding include co-interventions, differences at baseline in patient characteristics, and other issues throughout the questions above. High risk of bias translates to a rating of poor quality; low risk of bias translates to a rating of good quality. Again, the greater the risk of bias, the lower the quality rating of the study.

In addition, the more attention in the study design to issues that can help determine if there is a causal relationship between the exposure and outcome, the higher quality the study. These issues include exposures occurring prior to outcomes, evaluation of a dose-response gradient, accuracy of measurement of both exposure and outcome, and sufficient timeframe to see an effect.

Generally, when reviewers evaluate a study, they will not see a "fatal flaw," but instead will find some risk of bias. By focusing on the concepts underlying the questions in the quality assessment tool, reviewers should ask themselves about the potential for bias in the study they are critically appraising. For any box checked "no" reviewers should ask, "What is the potential risk of bias resulting from this flaw in study design or execution?" That is, does this factor lead to doubt about the results reported in the study or doubt about the ability of the study to accurately assess an association between the intervention or exposure and the outcome?

The best approach is to think about the questions in the assessment tool and how each one reveals something about the potential for bias in a study. Specific rules are not useful, as each study has specific nuances. In addition, being familiar with the key concepts will help reviewers be more comfortable with critical appraisal. Examples of studies rated good, fair, and poor are useful, but each study must be assessed on its own.

### Controlled Intervention Studies

| **Reference** | 1. Was the study described as randomized, a randomized trial, a randomized clinical trial, or an RCT? | 2. Was the method of randomization adequate (i.e., use of randomly generated assignment)? | 3. Was the treatment allocation concealed (so that assignments could not be predicted)? | 4. Were study participants and providers blinded to treatment group assignment? | 5. Were the people assessing the outcomes blinded to the participants' group assignments? | 6. Were the groups similar at baseline on important characteristics that could affect outcomes (e.g., demographics, risk factors, co-morbid conditions)? | 7. Was the overall drop-out rate from the study at endpoint 20% or lower of the number allocated to treatment? | 8. Was the differential drop-out rate (between treatment groups) at endpoint 15 percentage points or lower? | 9. Was there high adherence to the intervention protocols for each treatment group? | Were other interventions avoided or similar in the groups (e.g., similar background treatments)? | Were outcomes assessed using valid and reliable measures, implemented consistently across all study participants? | Did the authors report that the sample size was sufficiently large to be able to detect a difference in the main outcome between groups with at least 80% power? | Were outcomes reported or subgroups analysed prespecified (i.e., identified before analyses were conducted)? | Were all randomized participants analysed in the group to which they were originally assigned, i.e., did they use an intention-to-treat analysis? | **Quality Rating (Good, Fair or Poor) (see guidance)** | **Raters Initials** | **Comments** |
| --- | --- | --- | --- | --- | --- | --- | --- | --- | --- | --- | --- | --- | --- | --- | --- | --- | --- |
|  |  |  |  |  |  |  |  |  |  |  |  |  |  |  |  |  |  |
|  |  |  |  |  |  |  |  |  |  |  |  |  |  |  |  |  |  |

#### Guidance

1. Described as randomized

Was the study described as randomized? A study does not satisfy quality criteria as randomized simply because the authors call it randomized; however, it is a first step in determining if a study is randomized

2 and 3. Treatment allocation–two interrelated pieces

Adequate randomization: Randomization is adequate if it occurred according to the play of chance (e.g., computer generated sequence in more recent studies, or random number table in older studies).

Inadequate randomization: Randomization is inadequate if there is a preset plan (e.g., alternation where every other subject is assigned to treatment arm or another method of allocation is used, such as time or day of hospital admission or clinic visit, ZIP Code, phone number, etc.). In fact, this is not randomization at all–it is another method of assignment to groups. If assignment is not by the play of chance, then the answer to this question is no.

There may be some tricky scenarios that will need to be read carefully and considered for the role of chance in assignment. For example, randomization may occur at the site level, where all individuals at a particular site are assigned to receive treatment or no treatment. This scenario is used for group-randomized trials, which can be truly randomized, but often are "quasi-experimental" studies with comparison groups rather than true control groups. (Few, if any, group-randomized trials are anticipated for this evidence review.)

Allocation concealment: This means that one does not know in advance, or cannot guess accurately, to what group the next person eligible for randomization will be assigned. Methods include sequentially numbered opaque sealed envelopes, numbered or coded containers, central randomization by a coordinating centre, computer-generated randomization that is not revealed ahead of time, etc.

4 and 5. Blinding

Blinding means that one does not know to which group–intervention or control–the participant is assigned. It is also sometimes called "masking." The reviewer assessed whether each of the following was blinded to knowledge of treatment assignment: (1) the person assessing the primary outcome(s) for the study (e.g., taking the measurements such as blood pressure, examining health records for events such as myocardial infarction, reviewing and interpreting test results such as x ray or cardiac catheterization findings); (2) the person receiving the intervention (e.g., the patient or other study participant); and (3) the person providing the intervention (e.g., the physician, nurse, pharmacist, dietitian, or behavioural interventionist).

Generally placebo-controlled medication studies are blinded to patient, provider, and outcome assessors; behavioural, lifestyle, and surgical studies are examples of studies that are frequently blinded only to the outcome assessors because blinding of the persons providing and receiving the interventions is difficult in these situations. Sometimes the individual providing the intervention is the same person performing the outcome assessment. This was noted when it occurred.

6. Similarity of groups at baseline

This question relates to whether the intervention and control groups have similar baseline characteristics on average especially those characteristics that may affect the intervention or outcomes. The point of randomized trials is to create groups that are as similar as possible except for the intervention(s) being studied in order to compare the effects of the interventions between groups. When reviewers abstracted baseline characteristics, they noted when there was a significant difference between groups. Baseline characteristics for intervention groups are usually presented in a table in the article (often Table 1).

Groups can differ at baseline without raising red flags if: (1) the differences would not be expected to have any bearing on the interventions and outcomes; or (2) the differences are not statistically significant. When concerned about baseline difference in groups, reviewers recorded them in the comments section and considered them in their overall determination of the study quality.

7 and 8. Dropout

"Dropouts" in a clinical trial are individuals for whom there are no end point measurements, often because they dropped out of the study and were lost to follow-up.

Generally, an acceptable overall dropout rate is considered 20 percent or less of participants who were randomized or allocated into each group. An acceptable differential dropout rate is an absolute difference between groups of 15 percentage points at most (calculated by subtracting the dropout rate of one group minus the dropout rate of the other group). However, these are general rates. Lower overall dropout rates are expected in shorter studies, whereas higher overall dropout rates may be acceptable for studies of longer duration. For example, a 6-month study of weight loss interventions should be expected to have nearly 100 percent follow-up (almost no dropouts–nearly everybody gets their weight measured regardless of whether or not they actually received the intervention), whereas a 10-year study testing the effects of intensive blood pressure lowering on heart attacks may be acceptable if there is a 20-25 percent dropout rate, especially if the dropout rate between groups was similar. The panels for the NHLBI systematic reviews may set different levels of dropout caps.

Conversely, differential dropout rates are not flexible; there should be a 15 percent cap. If there is a differential dropout rate of 15 percent or higher between arms, then there is a serious potential for bias. This constitutes a fatal flaw, resulting in a poor quality rating for the study.

9. Adherence

Did participants in each treatment group adhere to the protocols for assigned interventions? For example, if Group 1 was assigned to 10 mg/day of Drug A, did most of them take 10 mg/day of Drug A? Another example is a study evaluating the difference between a 30-pound weight loss and a 10-pound weight loss on specific clinical outcomes (e.g., heart attacks), but the 30-pound weight loss group did not achieve its intended weight loss target (e.g., the group only lost 14 pounds on average). A third example is whether a large percentage of participants assigned to one group "crossed over" and got the intervention provided to the other group. A final example is when one group that was assigned to receive a particular drug at a particular dose had a large percentage of participants who did not end up taking the drug or the dose as designed in the protocol.

10. Avoid other intervention

Changes that occur in the study outcomes being assessed should be attributable to the interventions being compared in the study. If study participants receive interventions that are not part of the study protocol and could affect the outcomes being assessed, and they receive these interventions differentially, then there is cause for concern because these interventions could bias results. The following scenario is another example of how bias can occur. In a study comparing two different dietary interventions on serum cholesterol, one group had a significantly higher percentage of participants taking statin drugs than the other group. In this situation, it would be impossible to know if a difference in outcome was due to the dietary intervention or the drugs.

11. Outcome measures assessment

What tools or methods were used to measure the outcomes in the study? Were the tools and methods accurate and reliable–for example, have they been validated, or are they objective? This is important as it indicates the confidence you can have in the reported outcomes. Perhaps even more important is ascertaining that outcomes were assessed in the same manner within and between groups. One example of differing methods is self-report of dietary salt intake versus urine testing for sodium content (a more reliable and valid assessment method). Another example is using BP measurements taken by practitioners who use their usual methods versus using BP measurements done by individuals trained in a standard approach. Such an approach may include using the same instrument each time and taking an individual's BP multiple times. In each of these cases, the answer to this assessment question would be "no" for the former scenario and "yes" for the latter. In addition, a study in which an intervention group was seen more frequently than the control group, enabling more opportunities to report clinical events, would not be considered reliable and valid.

12. Power calculation

Generally, a study's methods section will address the sample size needed to detect differences in primary outcomes. The current standard is at least 80 percent power to detect a clinically relevant difference in an outcome using a two-sided alpha of 0.05. Often, however, older studies will not report on power.

13. Prespecified outcomes

Investigators should prespecify outcomes reported in a study for hypothesis testing–which is the reason for conducting an RCT. Without prespecified outcomes, the study may be reporting ad hoc analyses, simply looking for differences supporting desired findings. Investigators also should prespecify subgroups being examined. Most RCTs conduct numerous post hoc analyses as a way of exploring findings and generating additional hypotheses. The intent of this question is to give more weight to reports that are not simply exploratory in nature.

14. Intention-to-treat analysis

Intention-to-treat (ITT) means everybody who was randomized is analysed according to the original group to which they are assigned. This is an extremely important concept because conducting an ITT analysis preserves the whole reason for doing a randomized trial; that is, to compare groups that differ only in the intervention being tested. When the ITT philosophy is not followed, groups being compared may no longer be the same. In this situation, the study would likely be rated poor. However, if an investigator used another type of analysis that could be viewed as valid, this would be explained in the "other" box on the quality assessment form. Some researchers use a completers analysis (an analysis of only the participants who completed the intervention and the study), which introduces significant potential for bias. Characteristics of participants who do not complete the study are unlikely to be the same as those who do. The likely impact of participants withdrawing from a study treatment must be considered carefully. ITT analysis provides a more conservative (potentially less biased) estimate of effectiveness.

**General Guidance for Determining the Overall Quality Rating of Controlled Intervention Studies**

The questions on the assessment tool were designed to help reviewers focus on the key concepts for evaluating a study's internal validity. They are not intended to create a list that is simply tallied up to arrive at a summary judgment of quality.

Internal validity is the extent to which the results (effects) reported in a study can truly be attributed to the intervention being evaluated and not to flaws in the design or conduct of the study–in other words, the ability for the study to make causal conclusions about the effects of the intervention being tested. Such flaws can increase the risk of bias. Critical appraisal involves considering the risk of potential for allocation bias, measurement bias, or confounding (the mixture of exposures that one cannot tease out from each other). Examples of confounding include co-interventions, differences at baseline in patient characteristics, and other issues addressed in the questions above. High risk of bias translates to a rating of poor quality. Low risk of bias translates to a rating of good quality.

Fatal flaws: If a study has a "fatal flaw," then risk of bias is significant, and the study is of poor quality. Examples of fatal flaws in RCTs include high dropout rates, high differential dropout rates, no ITT analysis or other unsuitable statistical analysis (e.g., completers-only analysis).

Generally, when evaluating a study, one will not see a "fatal flaw;" however, one will find some risk of bias. During training, reviewers were instructed to look for the potential for bias in studies by focusing on the concepts underlying the questions in the tool. For any box checked "no," reviewers were told to ask: "What is the potential risk of bias that may be introduced by this flaw?" That is, does this factor cause one to doubt the results that were reported in the study?

NHLBI staff provided reviewers with background reading on critical appraisal, while emphasizing that the best approach to use is to think about the questions in the tool in determining the potential for bias in a study. The staff also emphasized that each study has specific nuances; therefore, reviewers should familiarize themselves with the key concept.

# References

1. Higgins JPT, Thomas J, Chandler J, et al. Cochrane Handbook for Systematic Reviews of Interventions. Version 6.0. <www.training.cochrane.org/handbook>. Updated.

2. Batalli-Këpuska A, Bajraktari G, Zejnullahu M, et al. Abnormal systolic and diastolic myocardial function in obese asymptomatic adolescents. *Int J Cardiol.* 2013;168(3):2347-2351.

3. Chinali M, de Simone G, Roman MJ, et al. Impact of obesity on cardiac geometry and function in a population of adolescents: the Strong Heart Study. *J Am Coll Cardiol.* 2006;47(11):2267-2273.

4. Imerbtham T, Thitiwuthikiat P, Jongjitwimol J, Nuamchit T, Yingchoncharoen T, Siriwittayawan D. Leptin Levels are Associated with Subclinical Cardiac Dysfunction in Obese Adolescents. *Diabetes Metab Syndr Obes.* 2020;13:925-933.

5. Guillemette L, Dart A, Wicklow B, et al. Cardiac structure and function in youth with type 2 diabetes in the iCARE cohort study: Cross-sectional associations with prenatal exposure to diabetes and metabolomic profiles. *Pediatr Diabetes.* 2020;21(2).

6. Harada K, Orino T, Takada G. Body mass index can predict left ventricular diastolic filling in asymptomatic obese children. *Pediatr Cardiol.* 2001;22(4):273-278.

7. Binnetoglu FK, Yildirim S, Topaloglu N, et al. Early detection of myocardial deformation by 2D speckle tracking echocardiography in normotensive obese children and adolescents. *Anatol J Cardiol.* 2015;15(2):151-157.

8. Bae HK, Choi HS, Sohn S, Shin H-J, Nam J-H, Hong YM. Cardiovascular screening in asymptomatic adolescents with metabolic syndrome. *J Cardiovasc Ultrasound.* 2015;23(1):10-19.

9. Ghandi Y, Sharifi M, Habibi D, Dorreh F, Hashemi M. Evaluation of left ventricular function in obese children without hypertension by a tissue Doppler imaging study. *Ann Pediatr Cardiol.* 2018;11(1):28-33.

10. Hansen PR, Andersen LJ, Rebelo AN, et al. Cardiovascular effects of 3 months of football training in overweight children examined by comprehensive echocardiography: a pilot study. *J Sports Sci.* 2013;31(13):1432-1440.

11. Jing L, Binkley CM, Suever JD, et al. Cardiac remodeling and dysfunction in childhood obesity: a cardiovascular magnetic resonance study. *J Cardiovasc Magn Reson.* 2016;18(1):28-28.

12. Mahfouz RA, Gomma A, Goda M, Safwat M. Relation of left atrial stiffness to insulin resistance in obese children: Doppler strain imaging study. *Echocardiography.* 2015;32(7):1157-1163.

13. Özkan EA, Khosroshahi HE, Kılıç M, Geçit UA, Domur E. Obesity-related cardiovascular behavior in children. *Eur Rev Med Pharmacol Sci.* 2016;20(8):1559-1565.

14. Vitarelli A, Martino F, Capotosto L, et al. Early myocardial deformation changes in hypercholesterolemic and obese children and adolescents: a 2D and 3D speckle tracking echocardiography study. *Medicine (Baltimore).* 2014;93(12):e71-e71.

15. Bacha F, Gidding SS, Pyle L, et al. Relationship of Cardiac Structure and Function to Cardiorespiratory Fitness and Lean Body Mass in Adolescents and Young Adults with Type 2 Diabetes. *Journal of Pediatrics.* 2016;177:159-+.

16. Levitt Katz L, Gidding SS, Bacha F, et al. Alterations in left ventricular, left atrial, and right ventricular structure and function to cardiovascular risk factors in adolescents with type 2 diabetes participating in the TODAY clinical trial. *Pediatr Diabetes.* 2015;16(1):39-47.

17. Luo X-X, Zhu Y, Sun Y, et al. Does Masked Hypertension Cause Early Left Ventricular Impairment in Youth? *Front Pediatr.* 2018;6:167-167.

18. Morka A, Szydlowski L, Moric-Janiszewska E, Mazurek B, Markiewicz-Loskot G, Stec S. Left Ventricular Diastolic Dysfunction Assessed by Conventional Echocardiography and Spectral Tissue Doppler Imaging in Adolescents With Arterial Hypertension. *Medicine (Baltimore).* 2016;95(8).

19. Navarini S, Bellsham-Revell H, Chubb H, Gu HT, Sinha MD, Simpson JM. Myocardial Deformation Measured by 3-Dimensional Speckle Tracking in Children and Adolescents With Systemic Arterial Hypertension. *Hypertension.* 2017;70(6):1142-1147.

20. Tran AH, Flynn JT, Becker RC, et al. Subclinical Systolic and Diastolic Dysfunction Is Evident in Youth With Elevated Blood Pressure. *Hypertension.* 2020;75(6):1551-1556.

21. Urbina EM, Khoury PR, McCoy C, Daniels SR, Kimball TR, Dolan LM. Cardiac and vascular consequences of pre-hypertension in youth. *J Clin Hypertens (Greenwich).* 2011;13(5):332-342.

22. Di Bonito P, Moio N, Scilla C, et al. Usefulness of the high triglyceride-to-HDL cholesterol ratio to identify cardiometabolic risk factors and preclinical signs of organ damage in outpatient children. *Diabetes Care.* 2012;35(1):158-162.

23. Alp H, Karaarslan S, Eklioğlu BS, Atabek ME, Baysal T. The effect of hypertension and obesity on left ventricular geometry and cardiac functions in children and adolescents. *J Hypertens.* 2014;32(6):1283-1292.

24. Alp H, Karaarslan S, Selver Eklioğlu B, Atabek ME, Altın H, Baysal T. Association between nonalcoholic fatty liver disease and cardiovascular risk in obese children and adolescents. *Can J Cardiol.* 2013;29(9):1118-1125.

25. Di Salvo G, Pacileo G, Del Giudice EM, et al. Abnormal myocardial deformation properties in obese, non-hypertensive children: an ambulatory blood pressure monitoring, standard echocardiographic, and strain rate imaging study. *Eur Heart J.* 2006;27(22):2689-2695.

26. Di Bonito P, Moio N, Sibilio G, et al. Cardiometabolic phenotype in children with obesity. *J Pediatr.* 2014;165(6):1184-1189.

27. Unnithan VB, Baynard T, Potter CR, et al. An exploratory study of cardiac function and oxygen uptake during cycle ergometry in overweight children. *Obesity (Silver Spring).* 2007;15(11):2673-2682.

28. Brienza C, Grandone A, Di Salvo G, et al. Subclinical hypothyroidism and myocardial function in obese children. *Nutr Metab Cardiovasc Dis.* 2013;23(9):898-902.

29. Akinci A, Karakurt C, Gurbuz S, et al. Association of cardiac changes with serum adiponectin and resistin levels in obese and overweight children. *Journal of Cardiovascular Medicine.* 2013;14(3):228-234.

30. Pieruzzi F, Antolini L, Salerno FR, et al. The role of blood pressure, body weight and fat distribution on left ventricular mass, diastolic function and cardiac geometry in children. *J Hypertens.* 2015;33(6):1182-1192.

31. Barbosa JAA, Mota CCC, Simões E Silva AC, Nunes MdCP, Barbosa MM. Assessing pre-clinical ventricular dysfunction in obese children and adolescents: the value of speckle tracking imaging. *Eur Heart J Cardiovasc Imaging.* 2013;14(9):882-889.

32. Radgoudarzi M, Pazouki A, Fahmfam Z, Soheilipour F. Evaluation of changes in cardiac dimensions and functional parameters and their association with anthropometric parameters and laboratory indices in obese adolescents. *Med Sci.* 2020;24(103):1520-1527.

33. Hirschler V, Acebo HLP, Fernandez GB, Ferradas S, Oestreicher K. Association between left atrial size and measures of adiposity among normal adolescent boys. *Pediatr Cardiol.* 2012;33(2):245-251.

34. De Marco M, de Simone G, Roman MJ, et al. Cardiac geometry and function in diabetic or prediabetic adolescents and young adults: the Strong Heart Study. *Diabetes Care.* 2011;34(10):2300-2305.

35. Gidding SS, Braffett BH, Shah RD, et al. Longitudinal Changes in Cardiac Structure and Function From Adolescence to Young Adulthood in Participants With Type 2 Diabetes Mellitus: The TODAY Follow-Up Study. *Circ Heart Fail.* 2020;13(6):e006685.

36. Haley JE, Zhiqian G, Philip KR, et al. Reduction in myocardial strain is evident in adolescents and young adults with obesity and type 2 diabetes. *Pediatr Diabetes.* 2020;21(2):243-250.

37. Azza MA, Ragab SH, Ismail NA, Awad MAM, Kandil ME. Echocardiographic assessment of epicardial adipose tissue in obese children and its relation to clinical parameters of the metabolic syndrome. *Journal of Clinical and Basic Cardiology.* 2011;14(1-4):7-11.

38. Floriańczyk T, Gołąbek-Dylewska M, Kucińska B, Werner B. Evaluation of left ventricular function in overweight children and teenagers with arterial hypertension and white coat hypertension. *Cardiol J.* 2019;26(4):343-349.

39. Di Bonito P, Capaldo B, Forziato C, et al. Central adiposity and left ventricular mass in obese children. *Nutr Metab Cardiovasc Dis.* 2008;18(9):613-617.

40. Kozakova M, Morizzo C, Bianchi V, Marchetti S, Federico G, Palombo C. Hemodynamic overload and intra-abdominal adiposity in obese children: Relationships with cardiovascular structure and function. *Nutr Metab Cardiovasc Dis.* 2016;26(1):60-66.

41. Gidding SS, Palermo RA, DeLoach SS, Keith SW, Falkner B. Associations of Cardiac Structure with Obesity, Blood Pressure, Inflammation, and Insulin Resistance in African-American Adolescents. *Pediatr Cardiol.* 2014;35(2):307-314.

42. Krustrup P, Hansen PR, Nielsen CM, et al. Structural and functional cardiac adaptations to a 10-week school-based football intervention for 9-10-year-old children. *Scand J Med Sci Sports.* 2014;24:4-9.

43. Shi J, Luo D, Weng H, et al. Optimally estimating the sample standard deviation from the five-number summary. *Res Synth Methods.* 2020.

44. Ghanem S, Mostafa M, Ayad S. Early echocardiography abnormalities in obese children and adolescent and reversibility of these abnormalities after significant weight reduction. *J Saudi Heart Assoc.* 2010;22(1):13-18.

45. Ozcetin M, Celikyay ZR, Celik A, Yilmaz R, Yerli Y, Erkorkmaz U. The importance of carotid artery stiffness and increased intima-media thickness in obese children. *S Afr Med J.* 2012;102(5):295-299.

46. Porcar-Almela M, Codoñer-Franch P, Tuzón M, Navarro-Solera M, Carrasco-Luna J, Ferrando J. Left ventricular diastolic function and cardiometabolic factors in obese normotensive children. *Nutr Metab Cardiovasc Dis.* 2015;25(1):108-115.

47. Van Putte-Katier N, Rooman RP, Haas L, et al. Early cardiac abnormalities in obese children: importance of obesity per se versus associated cardiovascular risk factors. *Pediatr Res.* 2008;64(2):205-209.

48. Singh GK, Vitola BE, Holland MR, et al. Alterations in ventricular structure and function in obese adolescents with nonalcoholic fatty liver disease. *J Pediatr.* 2013;162(6):1160-1168.e1161.

49. Sanchez AA, Levy PT, Sekarski TJ, et al. Markers of cardiovascular risk, insulin resistance, and ventricular dysfunction and remodeling in obese adolescents. *J Pediatr.* 2015;166(3):660-665.

50. Obert P, Gueugnon C, Nottin S, et al. Two-dimensional strain and twist by vector velocity imaging in adolescents with severe obesity. *Obesity (Silver Spring).* 2012;20(12):2397-2405.

51. Lorch SM, Sharkey A. Myocardial velocity, strain, and strain rate abnormalities in healthy obese children. *J Cardiometab Syndr.* 2007;2(1):30-34.

52. Kibar AE, Pac FA, Ece İ, et al. Effect of obesity on left ventricular longitudinal myocardial strain by speckle tracking echocardiography in children and adolescents. *Balkan Med J.* 2015;32(1):56-63.

53. Dias KA, Spence AL, Sarma S, et al. Left ventricular morphology and function in adolescents: Relations to fitness and fatness. *Int J Cardiol.* 2017;240:313-319.

54. Abraham TP, Dimaano VL, Liang HY. Role of tissue Doppler and strain echocardiography in current clinical practice. *Circulation.* 2007;116(22):2597-2609.

55. Corica D, Oreto L, Pepe G, et al. Precocious Preclinical Cardiovascular Sonographic Markers in Metabolically Healthy and Unhealthy Childhood Obesity. *Front Endocrinol (Lausanne).* 2020;11:56.

56. Cozzolino D, Grandone A, Cittadini A, et al. Subclinical myocardial dysfunction and cardiac autonomic dysregulation are closely associated in obese children and adolescents: the potential role of insulin resistance. *PloS one.* 2015;10(4):e0123916-e0123916.

57. Dahiya R, Shultz SP, Dahiya A, et al. Relation of reduced preclinical left ventricular diastolic function and cardiac remodeling in overweight youth to insulin resistance and inflammation. *Am J Cardiol.* 2015;115(9):1222-1228.

58. Franssen WMA, Beyens M, Hatawe TA, et al. Cardiac function in adolescents with obesity: cardiometabolic risk factors and impact on physical fitness. *Int J Obes.* 2019;43(7):1400-1410.

59. Brar PC, Chun A, Fan X, et al. Impaired myocardial deformation and ventricular vascular coupling in obese adolescents with dysglycemia. *Cardiovasc Diabetol.* 2019;18(1):172-172.

60. Xie L, Man E, Cheung P-T, Cheung Y-F. Myocardial Integrated Backscatter in Obese Adolescents: Associations with Measures of Adiposity and Left Ventricular Deformation. *PloS one.* 2015;10(10):e0141149-e0141149.

61. Shah AS, Khoury PR, Dolan LM, et al. The effects of obesity and type 2 diabetes mellitus on cardiac structure and function in adolescents and young adults. *Diabetologia.* 2011;54(4):722-730.

62. Eklioğlu BS, Atabek ME, Akyürek N, Alp H. Prediabetes and Cardiovascular Parameters in Obese Children and Adolescents. *J Clin Res Pediatr Endocrinol.* 2016;8(1):80-85.

63. Marcovecchio ML, Gravina M, Gallina S, et al. Increased left atrial size in obese children and its association with insulin resistance: a pilot study. *Eur J Pediatr.* 2016;175(1):121-130.

64. Akcaboy M, Kula S, Göktas T, et al. Effect of plasma NOx values on cardiac function in obese hypertensive and normotensive pediatric patients. *Pediatr Nephrol.* 2016;31(3):473-483.

65. Dušan P, Tamara I, Goran V, Gordana M-L, Amira P-A. Left ventricular mass and diastolic function in obese children and adolescents. *Pediatr Nephrol.* 2015;30(4):645-652.

66. Levent E, Gökşen D, Ozyürek AR, Darcan S, Coker M. Usefulness of the myocardial performance index (MPI) for assessing ventricular function in obese pediatric patients. *Turk J Pediatr.* 2005;47(1):34-38.

67. Akyol B, Boyraz M, Aysoy C. Relationship of epicardial adipose tissue thickness with early indicators of atherosclerosis and cardiac functional changes in obese adolescents with metabolic syndrome. *J Clin Res Pediatr Endocrinol.* 2013;5(3):156-163.

68. Aslan E, Sert A, Buyukinan M, et al. Left and right ventricular function by echocardiography, tissue Doppler imaging, carotid intima-media thickness, and asymmetric dimethyl arginine levels in obese adolescents with metabolic syndrome. *Cardiol Young.* 2019;29(3):310-318.

69. Chinali M, de Simone G, Roman MJ, et al. Cardiac markers of pre-clinical disease in adolescents with the metabolic syndrome: the strong heart study. *J Am Coll Cardiol.* 2008;52(11):932-938.

70. Di Bonito P, Moio N, Scilla C, et al. Preclinical manifestations of organ damage associated with the metabolic syndrome and its factors in outpatient children. *Atherosclerosis.* 2010;213(2):611-615.

71. Pacifico L, Di Martino M, De Merulis A, et al. Left ventricular dysfunction in obese children and adolescents with nonalcoholic fatty liver disease. *Hepatology.* 2014;59(2):461-470.

72. Sert A, Aypar E, Pirgon O, Yilmaz H, Odabas D, Tolu I. Left ventricular function by echocardiography, tissue Doppler imaging, and carotid intima-media thickness in obese adolescents with nonalcoholic fatty liver disease. *Am J Cardiol.* 2013;112(3):436-443.

73. Ahmed AY, Youssef OI, Farweez BA. Endothelial progenitor cells cut-off and relations to cardiovascular risk factors in obese children and adolescents. *Current Pediatric Research.* 2016;20(1-2):74-81.

74. Akyol B, Boyraz M, Aysoy C. Relationship of epicardial adipose tissue thickness with early indicators of atherosclerosis and cardiac functional changes in obese adolescents with metabolic syndrome. *J Clin Res Pediatr Endocrinol.* 2013;5(3):156-163.

75. Alkholy UM, Ahmed IA, Karam NA, Ali YF, Yosry A. Assessment of left ventricular mass index could predict metabolic syndrome in obese children. *J Saudi Heart Assoc.* 2016;28(3):159-166.

76. Alp H, Eklioğlu BS, Atabek ME, et al. Evaluation of epicardial adipose tissue, carotid intima-media thickness and ventricular functions in obese children and adolescents. *J Pediatr Endocrinol Metab.* 2014;27(9-10):827-835.

77. Battal F, Ermis B, Aktop Z, Can M, Demirel F. Early cardiac abnormalities and serum N-terminal pro B-type natriuretic peptide levels in obese children. *J Pediatr Endocrinol Metab.* 2011;24(9-10):723-726.

78. Bjornstad P, Truong U, Dorosz JL, et al. Cardiopulmonary Dysfunction and Adiponectin in Adolescents With Type 2 Diabetes. *J Am Heart Assoc.* 2016;5(3):e002804-e002804.

79. Boyraz M, Pirgon O, Akyol B, Dundar B, Cekmez F, Eren N. Importance of epicardial adipose tissue thickness measurement in obese adolescents, its relationship with carotid intima-media thickness, and echocardiographic findings. *Eur Rev Med Pharmacol Sci.* 2013;17(24):3309-3317.

80. Dhuper S, Abdullah RA, Weichbrod L, Mahdi E, Cohen HW. Association of obesity and hypertension with left ventricular geometry and function in children and adolescents. *Obesity (Silver Spring, Md).* 2011;19(1):128-133.

81. Di Bonito P, Forziato C, Sanguigno E, et al. Prehypertension in outpatient obese children. *Am J Hypertens.* 2009;22(12):1309-1313.

82. Di Salvo G, Pacileo G, Del Giudice EM, et al. Atrial myocardial deformation properties in obese nonhypertensive children. *J Am Soc Echocardiogr.* 2008;21(2):151-156.

83. El Saiedi SA, Mira MF, Sharaf SA, et al. Left ventricular diastolic dysfunction without left ventricular hypertrophy in obese children and adolescents: a Tissue Doppler Imaging and Cardiac Troponin I Study. *Cardiol Young.* 2018;28(1):76-84.

84. Harris KC, Al Saloos HA, De Souza AM, et al. Biophysical properties of the aorta and left ventricle and exercise capacity in obese children. *Am J Cardiol.* 2012;110(6):897-901.

85. Hirschler V, Acebo HLP, Fernandez GB, de Luján Calcagno M, Gonzalez C, Jadzinsky M. Influence of obesity and insulin resistance on left atrial size in children. *Pediatr Diabetes.* 2006;7(1):39-44.

86. Hui W, Slorach C, Guerra V, et al. Effect of Obstructive Sleep Apnea on Cardiovascular Function in Obese Youth. *Am J Cardiol.* 2019;123(2):341-347.

87. Ingul CB, Tjonna AE, Stolen TO, Stoylen A, Wisloff U. Impaired cardiac function among obese adolescents: effect of aerobic interval training. *Arch Pediatr Adolesc Med.* 2010;164(9):852-859.

88. Ingul CB, Dias KA, Tjonna AE, et al. Effect of High Intensity Interval Training on Cardiac Function in Children with Obesity: A Randomised Controlled Trial. *Prog Cardiovasc Dis.* 2018;61(2):214-221.

89. Ippisch HM, Inge TH, Daniels SR, et al. Reversibility of cardiac abnormalities in morbidly obese adolescents. *J Am Coll Cardiol.* 2008;51(14):1342-1348.

90. Kamal HM, Atwa HA, Saleh OM, Mohamed FA. Echocardiographic evaluation of cardiac structure and function in obese Egyptian adolescents. *Cardiol Young.* 2012;22(4):410-416.

91. Karaağaç AT, Yıldırım Aİ. How do diet and exercise programmes affect the cardiovascular risk profiles of obese children? *Cardiol Young.* 2019;29(2):200-205.

92. Kibar AE, Pac FA, Ballı S, et al. Early subclinical left-ventricular dysfunction in obese nonhypertensive children: a tissue Doppler imaging study. *Pediatr Cardiol.* 2013;34(6):1482-1490.

93. Kinik ST, Varan B, Yildirim SV, Tokel K. The effect of obesity on echocardiographic and metabolic parameters in childhood. *J Pediatr Endocrinol Metab.* 2006;19(8):1007-1014.

94. Koopman LP, McCrindle BW, Slorach C, et al. Interaction between myocardial and vascular changes in obese children: a pilot study. *J Am Soc Echocardiogr.* 2012;25(4):401-410.e401.

95. Korkmaz O, Gursu HA, Karagun BS. Comparison of echocardiographic findings with laboratory parameters in obese children. *Cardiol Young.* 2016;26(6):1060-1065.

96. Labombarda F, Zangl E, Dugue AE, et al. Alterations of left ventricular myocardial strain in obese children. *Eur Heart J Cardiovasc Imaging.* 2013;14(7):668-676.

97. Mangner N, Scheuermann K, Winzer E, et al. Childhood obesity: impact on cardiac geometry and function. *JACC Cardiovasc Imaging.* 2014;7(12):1198-1205.

98. Mehta SK, Holliday C, Hayduk L, Wiersma L, Richards N, Younoszai A. Comparison of myocardial function in children with body mass indexes >/=25 versus those <25 kg/m2. *Am J Cardiol.* 2004;93(12):1567-1569.

99. Mehta SK, Richards N, Lorber R, Rosenthal GL. Abdominal obesity, waist circumference, body mass index, and echocardiographic measures in children and adolescents. *Congenit Heart Dis.* 2009;4(5):338-347.

100. Metwalley KA, Farghaly HS, Sherief T. Plasma adrenomedullin level in children with obesity: relationship to left ventricular function. *World J Pediatr.* 2018;14(1):84-91.

101. Naylor LH, Watts K, Sharpe JA, et al. Resistance Training and Diastolic Myocardial Tissue Velocities in Obese Children. *Med Sci Sports Exerc.* 2008;40(12):2027-2032.

102. Obert P, Gueugnon C, Nottin S, et al. Impact of diet and exercise training-induced weight loss on myocardial mechanics in severely obese adolescents. *Obesity (Silver Spring).* 2013;21(10):2091-2098.

103. Ozdemir O, Hizli S, Abaci A, Agladioglu K, Aksoy S. Echocardiographic measurement of epicardial adipose tissue in obese children. *Pediatr Cardiol.* 2010;31(6):853-860.

104. Saltijeral A, Isla LP, Perez-Rodriguez O, et al. Early myocardial deformation changes associated to isolated obesity: a study based on 3D-wall motion tracking analysis. *Obesity (Silver Spring).* 2011;19(11):2268-2273.

105. Saritas T, Tascilar E, Abaci A, et al. Importance of plasma N-terminal pro B-type natriuretic peptide, epicardial adipose tissue, and carotid intima-media thicknesses in asymptomatic obese children. *Pediatr Cardiol.* 2010;31(6):792-799.

106. Schuster I, Karpoff L, Perez-Martin A, et al. Cardiac function during exercise in obese prepubertal boys: effect of degree of obesity. *Obesity (Silver Spring).* 2009;17(10):1878-1883.

107. Schusterova I, Jurko A, Minarik M. Left ventricular systolic and diastolic function in children with overweight and obesity. *Bratisl Lek Listy.* 2013;114(9):526-530.

108. Shah AS, Dolan LM, Khoury PR, Gao Z, Kimball TR, Urbina EM. Severe Obesity in Adolescents and Young Adults Is Associated With Subclinical Cardiac and Vascular Changes. *J Clin Endocrinol Metab.* 2015;100(7):2751-2757.

109. Sharpe JA, Naylor LH, Jones TW, et al. Impact of obesity on diastolic function in subjects < or = 16 years of age. *Am J Cardiol.* 2006;98(5):691-693.

110. Whalley GA, Gusso S, Hofman P, et al. Structural and functional cardiac abnormalities in adolescent girls with poorly controlled type 2 diabetes. *Diabetes care.* 2009;32(5):883-888.

111. Yang M-C, Liu H-K, Su Y-T, Tsai C-C, Wu J-R. Serum apoptotic marker M30 is positively correlated with early diastolic dysfunction in adolescent obesity. *PloS one.* 2019;14(5):e0217429-e0217429.

112. Yıldırım Ö, Demircan T, Tüfekçi Ö, et al. Anemia and Its Effect on Cardiovascular Findings in Obese Adolescents. *Turk J Haematol.* 2018;35(3):192-196.

113. Yu JJ, Yeom HH, Chung S, Park Y, Lee DH. Left atrial diameters in overweight children with normal blood pressure. *J Pediatr.* 2006;148(3):321-325.

114. Zeybek C, Celebi A, Aktuglu-Zeybek C, et al. The effect of low-carbohydrate diet on left ventricular diastolic function in obese children. *Pediatr Int.* 2010;52(2):218-223.

115. Zhang CQ, Deng YW, Liu YN, et al. Preclinical cardiovascular changes in children with obesity: A real-time 3-dimensional speckle tracking imaging study. *Plos One.* 2018;13(10):e0205177.

116. NIH National Heart Lung and Blood Institute. Study Quality Assessment Tools. National Heart, Lung, and Blood Institute (NHLBI). <https://www.nhlbi.nih.gov/health-topics/study-quality-assessment-tools>. Accessed 11th July, 2020.
